# Significantly fewer protein functional changing variants for lipid metabolism in Africans than in Europeans

**DOI:** 10.1186/1479-5876-11-67

**Published:** 2013-03-20

**Authors:** Cheng Xue, Xiaoming Liu, Yun Gong, Yuhai Zhao, Yun-Xin Fu

**Affiliations:** 1 Human Genetics Center, University of Texas Health Science Center at Houston, Houston, TX 77030, USA; 2 GuangDong Institute for Monitoring Laboratory Animals, Guangzhou 510260, China; 3 Medical School, University of Texas Health Science Center at Houston, Houston, TX 77030, USA; 4 Laboratory for Conservation and Utilization of Bio-resources, Yunnan University, Yunnan 650223, China

## Abstract

**Background:**

The disorders in metabolism of energy substances are usually related to some diseases, such as obesity, diabetes and cancer, *etc*. However, the genetic background for these disorders has not been well understood. In this study, we explored the genetic risk differences among human populations in metabolism (catabolism and biosynthesis) of energy substances, including lipids, carbohydrates and amino acids.

**Results:**

Two genotype datasets (Hapmap and 1000 Genome) were used for this study. The genetic risks of protein functional changing variants (PFCVs) on genes involved in lipid, carbohydrate and amino acid metabolism were calculated using two genetic risk indices: the total number of PFCVs (Num) and the total possibly harmful score of PFCVs (R). Observations in these two genotype datasets consistently showed that Africans had lower genetic risk in lipid metabolism (both catabolic and biosynthetic processes) compared to Europeans. However this relationship was not observed in carbohydrate and amino acid metabolism.

**Conclusions:**

Our results suggested that Africans had higher efficiency of utilizing lipids as energy substances than Europeans. In other words, lipids might be more preferred as energy substances in Africans than in Europeans.

## Introduction

Many complex diseases are closely related to the disorders in energy substances metabolism. Among three main energy substances (carbohydrates, lipids, and amino acids), lipids are often majorly studied because observed abnormalities in their metabolism can induce complex diseases, such as obesity, diabetes, and cancer, *etc.*[1-3]. The genetic background of lipid metabolism might be at the core of understanding these complex diseases.

It is usually easier to understand genetic differences at population level than at individual level. It has been well reported that the prevalence of many complex diseases, such as obesity, coronary heart diseases, and hypertension, *etc*. are race related and the genetic risks associated with these diseases might be different among different populations [4-8]. For example, the prevalence of hypertension among African-Americans is 1.5–2-fold higher than European-Americans [9].

Many kinds of genetic robustness, such as duplicate genes and biologically complicated networks, exist in human genomes [5,10-13], which explain why loss of functions of one or more genes have little phenotypic effect. However, the accumulation of protein functional changing variants (PFCVs) might reduce or even destroy this robustness [5,10,13]. The vast majority of complex diseases have many genes involved, to which each gene only contributes a very small amount of effect [14]. Therefore, more PFCVs on genes involved in energy metabolisms might lead to higher genetic risk developing complex diseases at population level [5].

In our previous study with Hapmap data [5], we reported that Africans had significantly fewer PFCVs in whole catabolic process than non-Africans. In this study, with the use of two genotype datasets (Hapmap and 1000 Genome), we further investigated the genetic risks (R and Num) in metabolic processes of three main energy substances (carbohydrates, lipids and amino acids) among human populations. Results showed that R and Num for lipids in both catabolic and biosynthetic processes were significantly smaller in Africans than in Europeans. However, for catabolic or biosynthetic process of carbohydrates and amino acids, R and Num were either larger or not smaller in Africans compared to Europeans. Based on these observations, we hypothesized that Africans had higher efficiency of utilizing lipids as energy substances than Europeans and proposed a study design for testing this hypothesis.

## Materials and methods

### Genotype data

Hapmap genotype, downloaded from HapMap project, was sampled from 11 human groups (Hapmap Public Release #3 on May 28, 2010, http://hapmap.ncbi.nlm.nih.gov/). The detailed information about these human groups are listed in Table 1. In brief, Human population = {African, East Asian, European, GIH, MEX}, in which African = {ASW, LWK, MKK, YRI}, East Asian = {CHB, CHD, JPT}, European = {CEU, TSI}, and GIH and MEX are two independent groups. The genotype data in some human groups (ASW, CEU, MXL, MKK and YRI) contained family trios data which could bias the results. Therefore the offspring for each trio of these groups were excluded from further analyses. Table 1 shows the final data set used for this study.

**Table 1:** Overview of Genotype datasets used in this study

**Dataset**	**Subpopulation**	**Group name**	**Group symbol**	**Total sample number (n)**	**Trio-excluded sample number (n)**	**Total number of SNPs**	**The number of SNPs with missense mutation**	**The number of SNPs with harmful (ri>0.2) missense mutation**
Hapmap released in May, 2010	European	Utah residents with Northern and Western European ancestry from the CEPH collection	CEU	165	117	1397814	11185	3788
		Toscans in Italy	TSI	102	102	1419970	11160	3778
		Han Chinese in Beijing, China	CHB	137	137	1341772	12205	4441
	Asian	Chinese in Metropolitan Denver, Colorado	CHD	109	109	1311767	9907	3263
		Japanese in Tokyo, Japan	JPT	113	113	1294406	11008	3887
	African	African ancestry in Southwest USA	ASW	87	75	1543115	12190	4090
		Luhya in Webuye, Kenya	LWK	110	110	1526783	13518	4861
		Maasai in Kinyawa, Kenya	MKK	184	156	1532002	11694	3836
		Yoruba in Ibadan, Nigeria	YRI	203	149	1493761	11583	3880
	Independent groups	Gujarati Indians in Houston, Texas	GIH	101	101	1408904	10491	3471
		Mexican ancestry in Los Angeles, California	MEX	86	58	1453054	12770	4562
1000 Genomes, pilot, low-coverage, released in July, 2010	European	Utah residents with Northern and Western European ancestry from the CEPH collection	CEU	60	60	7724854	86040	14807
	Asian	Han Chinese in Beijing, China and Japanese in Tokyo, Japan	CHB+JPT	60	60	6107825	60584	9912
	African	Yoruba in Ibadan, Nigeria	YRI	59	59	10556156	119798	21055

1000 Genome low-coverage genotype data (released in July, 2010), downloaded from 1000 Genome project (ftp://ftp-trace.ncbi.nih.gov/1000genomes/ftp/pilot_data/release/2010_07/low_coverage/snps/), was sampled from CEU (European-American), CHB and JPT(East Asian), and YRI (African) (Table 1).

### Genotype data preparation and genetic risk estimation

The preparation for the studied genotype data, the methods used for PFCVs selection, and the estimation of the genetic risks (R and Num) were described in details in our previous study [5]. Here we only focus briefly on the points specifically for this study.

The missense mutations of genes that were involved in metabolism of carbohydrates, lipids and amino acids were mainly considered in this study. The harmful impacts of many missense mutations over the genes were estimated using Polyphen-2 [15] collected in dbNSFP [16]. For simplicity, the alleles with minor allele frequency (MAF) were called mutations throughout this article.

The genes involved in the metabolic processes of carbohydrates, lipids, and amino acids were downloaded from Gene Ontology (GO) (http://www.geneontology.org). The name, symbol, and chromosome location of these selected genes are shown in details in Additional file 1: Table S1-S6 of supporting information. The total number of genes for each GO term is shown in Table 2. PFCVs with missense mutations on these genes were downloaded from NCBI dbSNP database (ftp://ftp.ncbi.nih.gov/snp/). The harmful probability for each PFCV estimated by Polyphen-2 was downloaded from http://genetics.bwh.harvard.edu/pph2/dbsearch.shtml. For a false positive rate of 20%, the true positive prediction rate in PolyPhen-2 trained on HumDiv dataset is 92%, so the HumDiv-trained score for each mutation is referenced [15].

**Table 2:** The number of genes in energy expenditure and storage processes in Gene ontology

	**Energy materials**	**GO id**	**Gene number**
Energy expenditure (Catabolic process)	Carbohydrates	GO:0016052	144
	Lipids	GO:0016042	218
	Amino acids	GO:0009310	96
Energy storage (Biosynthetic process)	Carbohydrates	GO:0016051	185
	Lipids	GO:0008610	424
	Amino acids	GO:0006412	426
	(translation)		

Two indices, R and Num of PFCVs, were used to assess the genetic risk in metabolic processes of carbohydrates, lipids and amino acids. The methods used to calculate R and Num were described in details in our previous study [5].

### Permutation test

In this study, we used permutation to reduce the background risk level when assessing the actual genetic risks. Total 18161 genes in human genome were used for the assessment and they were downloaded from http://www.geneontology.org. Two types of permutation tests were conducted in this study. 1. Gene-based permutation. Of these 18161 genes, the given number of genes (the number of genes involved in carbohydrate, lipid or amino acid metabolism (catabolism and biosynthesis), Table 2) were re-sampled randomly up to 2000 times, followed by the calculations of R and Num for these genes. 2. PFCV-based permutation. The number of harmful PFCVs on a given set of genes (the set of genes involved in the carbohydrate, lipid or amino acid metabolism, Table 2) were counted and recorded. The same number of PFCVs was re-sampled randomly from total PFCVs of 18161 human genes up to 2000 times, followed by the calculations of R and Num on these re-sampled PFCVs. Because the results from these two tests were very similar [5], in this article we only showed and discussed the results obtained by using the gene-based permutation test. The results of these permutation tests were used as an estimation to the background risk when analyzing data.

For example, the total number of genes involved in lipid catabolic process for Africans and Europeans was 218 (Table 2), therefore 218 genes were re-sampled randomly each time from 18161 human genes and R and Num were calculated on these genes as background risk level of R and Num for Africans and Europeans. At each round of re-sampling, we calculated the mean of R (or Num) and the mean difference R’ (R’ = mean R_African_ – mean R_Europan_). Total 2000 of R’ were obtained for each population for 2000 re-sampling processes and the distribution of R’ was close to normal (Additional file 2: Figure S1-S12). The actual observed mean difference (R’_lipid_catabolism_) for lipid catabolic process was also calculated. *P-Value* was approximately equal to the number of re-sampled with R’< R’_lipid_catabolism_ divided by 2000.

### Statistical methods

The unpaired two-tailed Student’s test, the F test (ANOVA, Analysis of Variance) and permutation tests were performed to assess the genetic risks among three subpopulations (Africans, Europeans and Asians) and 11 human groups (ASW, LWK, MKK, YRI, CHB, CHD, JPT, CEU, TSI, GIH and MEX).

## Results

### Overview of the studied data

In this study, two genotype datasets, Hapmap and 1000 Genome, were used for the analyses. A few characteristics existed between these two datasets. First, Hapmap had larger number of population groups (three subpopulations with 11 human groups) while 1000 Genome only had three subpopulations with four human groups. Second, sample sizes were similar between Hapmap (58 ≤ size ≤ 156) and 1000 Genome (59 for YRI and 60 for CEU and CHB and JPT). Third, 1000 Genome had the most PFCVs, including total number of PFCVs and total number of PFCVs with missense mutation (Table 1). These features indicated that some differences existed between Hapmap and 1000 Genome, regarding the number of human groups, the density of PFCVs, and the sample size. As a result, we explained the outcomes for each dataset separately although we used the same methods to analyze these data.

### Hapmap

**Figure 1:**
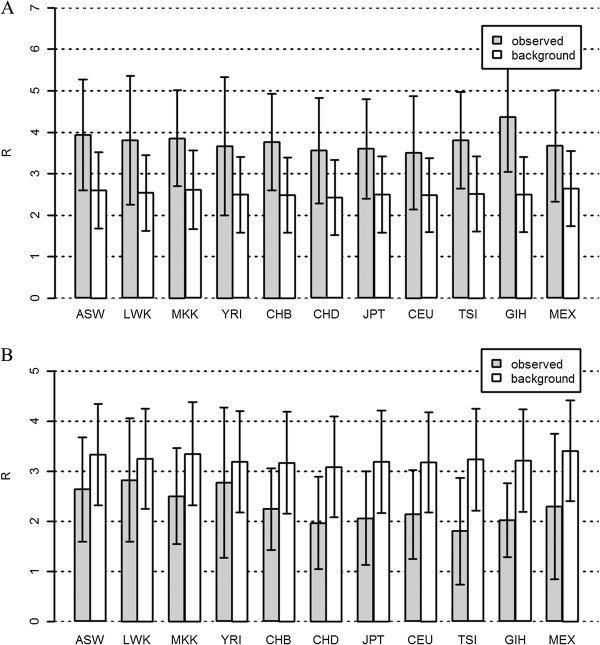
**The observed R (grey bar) and the background (white bar) R on protein functional changing variants (PFCVs) for Hapmap dataset in carbohydrate catabolic and biosynthetic processes (mean±SD).** The background Rs were obtained using permutation test. **A**: Results for carbohydrate catabolic process. **B**: Results for carbohydrate biosynthetic process.

**Figure 2:**
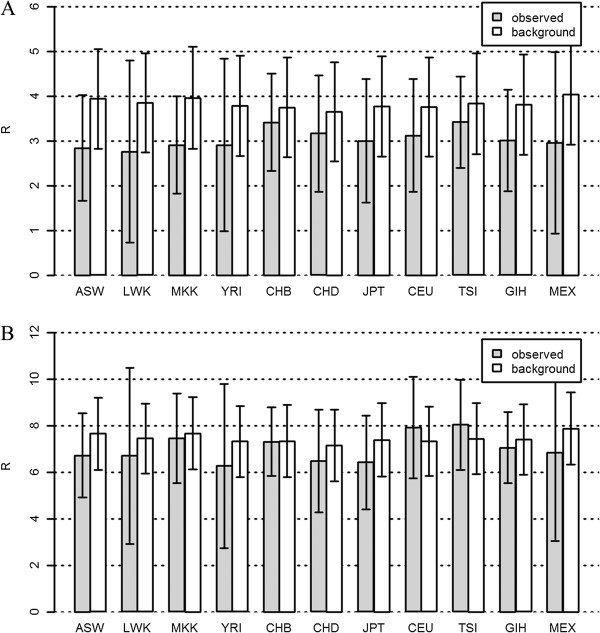
**The observed R (grey bar) and the background R (white bar) on protein functional changing variants (PFCVs) for Hapmap dataset in lipid catabolic and biosynthetic processes (mean±SD).** The background Rs were obtained using permutation test. **A**: Results for lipid catabolic process. **B**: Results for lipid biosynthetic process.

**Figure 3:**
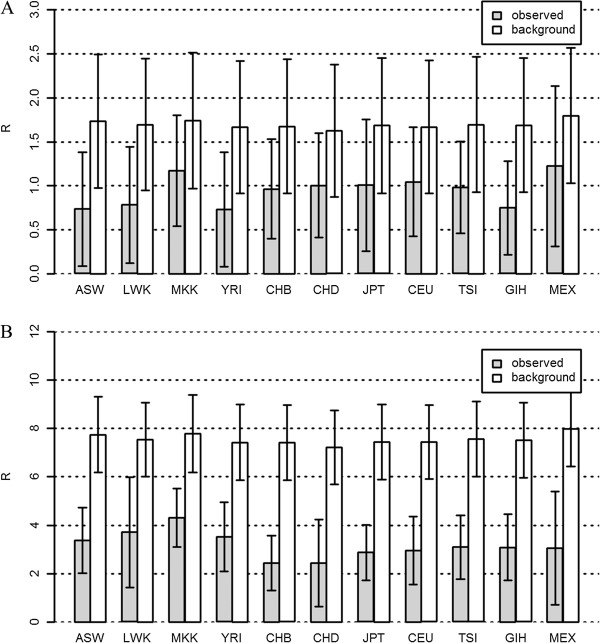
**The observed R (grey bar) and the background R (white bar) on protein functional changing variants (PFCVs) for Hapmap dataset in amino acid catabolic and biosynthetic processes (mean±SD).** The background Rs were obtained using permutation test. **A**: Results for amino acid catabolic process. **B**: Results for amino acid biosynthetic process.

Our results showed that in carbohydrate metabolism, most of the background R (on genes randomly sampled from human genomes using permutation test) was bigger than the observed R (on genes involved in metabolism of energy substances) (Figures 1, 2 and 3). It was intriguing that the observed R in carbohydrate catabolic process was significantly bigger than the background R (*P* << 0.01, Figure 1A). This result suggested that carbohydrate catabolic process might specially harbor more genetic mutations than expected (average on background). We also implemented the comparison of the observed R in catabolic and biosynthetic processes of carbohydrate among subpopulations (African, European and Asian) and among 11 human groups. The results showed no significant differences among subpopulations (*P* = 0.1903, F test, Table 3) in carbohydrate catabolic process, suggesting that the genetic risk R in carbohydrate catabolic process might be very similar among Africans, Europeans and Asians. However, among 11 human groups in carbohydrate catabolic process, the observed R in GIH was significantly bigger than all other human groups (*P* < 0.01, t-test, Figure 1A). In carbohydrate biosynthetic process, the observed R was significantly different among subpopulations (*P* < 2.2 × 10^-16^, F test, Table 3), and the R for all African groups was significantly bigger than non-African groups. (*P* < 0.01, t-test, all pairs, Figure 1B), suggesting that the genetic risk R in carbohydrate biosynthetic process in Africans might be the largest among human groups.

**Table 3:** **Analysis of Variance for Observations (ANOVA) of R estimated on genes in carbohydrate catabolic and biosynthetic process based on Hapmap data **^**a**^

**Metabolic Process**	**Item**	**Df.**	**Sum Sq.**	**Mean Sq.**	**F value**	**Pr(>F)**
Catabolic process	gender	1	0	0.00333	0.0019	0.9654
	subpopulation	2	5.87	2.93506	1.6619	0.1903
	group	6	12.49	2.08155	1.1786	0.3152
	gender x subpopulation	2	1.24	0.62057	0.3514	0.7038
	gender x group	6	8.39	1.39836	0.7918	0.5764
	Residuals	1050	1854.45	1.76614		
Biosynthetic process	gender	1	0.01	0.007	0.0061	0.93769
	subpopulation	2	103.55	51.777	45.0708	< 2.2 x 10^-16^^***^
	group	6	19.33	3.221	2.8038	0.01037 ^*^
	gender x subpopulation	2	1.25	0.624	0.5432	0.58106
	gender x group	6	4.19	0.698	0.6075	0.72451
	Residuals	1050	1206.24	1.149		

* The difference is greatly significant (P<0.05).

*** The difference is greatly significant (P<<0.01).

^a^ The data in African (ASW, LWK, MKK and YRI), East Asian (CHB, CHD and JPT) and European (CEU and TSI) are used for this analysis.

The observed R in lipid catabolic process was significantly smaller than the background R for each human group (*P* << 0.01, t-test, all pairs, Figure 2A), while the observed R in lipid biosynthetic process was also significantly smaller than the background R among most human groups with two exceptions (CEU and TSI) whose observed R were significantly bigger ( *P* <0.05, t-test, Figure 2B). In lipid catabolic process, the observed R in Africans was significantly smaller than all non-African groups (*P* < 0.05, t-test, all pairs, Figure 2A) with the fact that LWK of Africans held the smallest R (Figure 2A). In lipid biosynthetic process, the observed R in Africans was significantly smaller than Europeans (*P* < 0.01, Figure 2B). As far as the relationship of the observed R between Africans (ASW, LWK, MKK, YRI) and Asians (CHB, CHD, JPT), several different observations were obtained. First, the observed R in ASW and LWK was significantly smaller than CHB or smaller than CHD and JPT with no significance. Second, the observed R in YRI was significantly smaller than all Asian groups (CHB, CHD, and JPT). Third, MKK was the only group whose observed R was not significantly different from all Asian groups (Figure 2B). These observations suggested that the genetic risk R for lipid biosynthetic process in Africans might be smaller than all European groups and most of Asian groups.

The observed R in amino acid metabolic processes was consistently smaller than the background R. Especially, the ratio of R (the observed to the background) in the amino acid biosynthetic process was the smallest among all studied metabolic processes (Figures 1, 2 and 3), which suggested that, compared to the metabolism of carbohydrates and lipids, the biosynthesis of amino acids were more conservative and might harbor fewer mutations. In amino acid catabolic process, the observed R in most African groups were significantly smaller than Europeans and Asians (*P* < 0.01) with the exception of MKK whose R was significantly bigger than others (*P* < 0.01, Figure 3A). In amino acid biosynthetic process, the observed R in Africans was significantly bigger than non-Africans (*P* < 0.05), especially R in LWK, MKK and YRI were much bigger than other human groups (*P* < 0.01, Figure 3B). The observed R of Asians in amino acid biosynthetic process was the smallest among all studied human groups.

Additionally, we also implemented the comparison of the genetic risks (R and Num) between males and females for all human groups and results showed that there were no significant difference between males and females (*P* > 0.05, t- test, Table 3).

The above results for observed R were obtained without using the adjustment for background risk level. However, even with the consideration of background risk level, we still got the similar results (Additional file 2: Figure S13-S15).

### 1000 Genome (low-coverage, pilot data)

1000 Genome provides more variants than Hapmap (Table 1). Therefore here we used 1000 Genome data to replicate the results obtained by using Hapmap data. Without considering background risk level, mean R’ or mean Num’ observed between Africans-Europeans or Africans-Asians (mean R_African_ – mean R_European_ or mean Num_African_ – mean Num_European_) was observed to be bigger than 0 in 1000 Genome (Additional file 2: Figure S4-S15). This observation was different from the results observed using Hapmap data (see above) which might be resulted from the difference of total PFCVs between Hapmap and 1000 Genome (Table 1). After adjusting the background risk level using permutation test, we observed that R and Num in both lipid catabolic and lipid biosynthetic processes were significantly smaller in Africans than in Europeans (*P* < 0.05, permutation test, Table 4, Additional file 2: Figure S5 and S8). However, for carbohydrate and amino acid metabolisms, the significant difference of R or Num between Africans and Europeans was not observed in either catabolic or biosynthetic process (*P* > 0.05, Table 4, Additional file 2: Figure S4-S15). We also observed that R and Num in lipid catabolic process were smaller in Africans than in Asians with no significance (*P* = 0.099 for R; *P* = 0.0125 for Num). but this relationship was not observed in lipid biosynthetic process between Africans and Asians (*P* = 0.797 for R; *P* = 0.865 for Num) (Table 4, Additional file 2: Figure S11 and S14). The above observations suggested that the genetic risks (R or Num) in lipid catabolic and lipid biosynthetic processes were smaller in Africans than in Europeans, while in metabolisms of carbohydrates and amino acids, this relationship was not held.

**Table 4:** **Statistic results of permutation test for the difference of mean R in carbohydrate, lipid and amino acid metabolism between African and European (or Asian) with the consideration of background level in 1000 Genome data **^**a**^

**Index**	**Metabolism**	**Energy materials**	**1000 Genome data**			
			**African *Vs. *European**		**African *Vs. *Asian**	
			**R'**_**African-European**_	***P-value***	**R'**_**African-Asian**_	***P-value***
R	Energy expenditure (Catabolic process)	Carbohydrates	-0.2307	0.0535	2.947	0.4925
		Lipids	0.5753	0.072	1.93	0.099
		Amino acids	0.8928	0.3275	0.545	0.111
	Energy storage (Biosynthetic process)	Carbohydrates	3.642	0.718	3.066	0.324
		Lipids	1.798	0.0385*	11.25	0.797
		Amino acids	4.236	0.213	9.857	0.6485
Num	Energy expenditure (Catabolic process)	Carbohydrates	0.6644	0.12	8.45	0.5425
		Lipids	-0.865	0.03*	1.24	0.0115*
		Amino acids	3.516	0.483	1.34	0.0975
	Energy storage (Biosynthetic process)	Carbohydrates	9.85	0.735	7.43	0.2535
		Lipids	4.857	0.048*	30.45	0.865
		Amino acids	6.0	0.0725	21.93	0.3775

^a^ R’ is the difference of mean genetic risk in carbohydrate, lipid and amino acid metabolism between African and European (or Asian); *P-value* is P_(R’<R’African-European)_ for the test for the difference between African and European, which means the probability of the difference (R’) between African and European in permutation test being smaller than the difference observed in a given metabolic process.

* means *P-Value*<0.05.

We hypothesized that in order to respond to high-energy food environment, the smaller genetic risks among Africans in lipid metabolism might be translated to higher efficience of utilizing lipids as energy substances metabolism compared to Europeans.

This hypothesis could be tested with a simple experimental design. Triglycerides (TGs), the main energy substances in lipids, are usually biosynthesized in liver and are transported in blood as part of lipoprotein particles to the end of body for energy expenditure or energy storage [3,17]. With smaller genetic risks (R and Num) among Africans in lipid biosynthetic process, the efficiency of TGs biosynthesis in liver cells might be higher compared to Europeans [5,10]. Thus based on our hypothesis, the first clinical prediction should be that TGs level in arm arterial serum among Africans should be higher compared to Europeans . It was reported clinically that TGs level in arm venous serum was lower in Africans than in Europeans [18]. The difference of TGs between arterial and venous serum (TG_arterial-venous_) was usually larger than 0 [19], which suggested that it might not be appropriate to infer TGs in arterial serum using TGs in venous serum. TG_arterial-venous_ represented the net consumption of TGs in lipids expenditure and storage. Our second clinical prediction should be that TG_arterial-venous_ would be higher in Africans than in Europeans. Therefore, the data of TGs in arterial and venous serum obtained at the same time can be used to examine the predictions of our hypothesis.

## Discussion

It has been well known that background genetic risks are usually different among different human populations such that one population could have more genetic variants than others [5,13,20]. Our previous [5] and current study observed that Africans had more background genetic risk than Europeans. Some studyies reported that Africans had smaller proportion of homozygous mutations but bigger proportion of heterozygous compared to Europeans [20], suggesting that the excessive mutations in background for Africans might be shelved under the recessive model. Generally speaking, the difference of background genetic risk among populations might not result in possible racial difference for fitness or some other phenotypes. But the difference of genetic variant distribution in some body systems (for example lipid metabolism) might result in racial difference in some phenotypes (for example obesity, diabetes and cancer). To access racial difference of genetic variants in these systems among human populations, we had to remove the background noise through permutation test to make sure whether the racial differences of R or Num we observed in lipid metabolism were attributed to the background level or not.

If our hypothesis (Africans on average having higher efficiency of utilizing lipids during energy expenditure and storage) hold true, then lipids might be the more preferred energy substances for Africans compared to Europeans. As lipids are higher energy biomolecules compared to carbohydrates and proteins (the energy level for carbohydrates or proteins is ~4 calories per gram, while for lipids it is ~9 calories per gram), the preference of lipids as energy substances during energy expenditure might result in more economic energy generation in Africans compared to non-Africans. Clinically, Hunter *et al.* (2000) reported that when doing the same activity, African-descendant women consumed lower volumes of metabolically active masses [21]. This observation corresponded with our hypothesis of the preference of lipids as energy substances in Africans. Higher efficiency in energy expenditure along with lower consumption of body masses might contribute jointly to higher prevalence of obesity in Africans in high-energy food environment [4]. Meanwhile, more preference of lipids in energy storage might provoke the accumulation of fats in body.

In this study, we observed significantly fewer PFCVs in lipid metabolism among Africans than Europeans. Because most of PFCVs (or missense mutations) are detrimental, fewer PFCVs on genes in lipid metabolism might increase the efficiency of utilizing lipids as energy substances in Africans. This observation might extensively explain the differences of blood lipid level and its related phenotypes (or diseases) among human groups [8,9]. Of course, a lot of further studies are needed to elucidate the relationship between the PFCVs on the genes and the phenotypes (or diseases) associated with lipid metabolism. Of these studies, the study design we proposed in this paper to test the hypothesis could be the first step towards that goal.

## Competing interests

The authors declare no conflict of interest.

## Authors’ contributions

Designed research: CX, XL and Y-XF; Performed research: CX; Contributed new reagents or analytic tools: XL and Y-XF; Analyzed data: CX, XL, YZ and YG; Wrote the paper: CX and YG. All authors read and approved the final manuscript.

## Supplementary Material

Additional file 1 The list of genes in carbohydrate, lipid and amino acid metabolic (catabolic and biosynthetic) process. Click here for file

Additional file 2 Distribution of R’ [the difference of mean R between two populations] in permutation test and the observed and background genetic risk (Num) for Hapmap dataset in carbohydrate, lipid and amino acid metabolic (catabolic and biosynthetic) processes. Click here for file
