# Supplementary material for: Significantly fewer protein functional changing variants for lipid metabolism in Africans than in Europeans
Source: J Transl Med. 2013 Mar 20;11:67. doi: 10.1186/1479-5876-11-67 (PMC3610238; doi:10.1186/1479-5876-11-67)
Supplement: Additional file 1 — The list of genes in carbohydrate, lipid and amino acid metabolic (catabolic and biosynthetic) process. [file 1479-5876-11-67-S1.doc]

Significantly fewer protein changing variants for lipid metabolism in Africans than in Europeans

Cheng Xue^*, **^, Xiaoming Liu^*^, Yun Gong^*^, Yuhai Zhao^§^ and Yun-Xin Fu^*,§§^

^*^ Human Genetics Center, University of Texas Health Science Center at Houston, Houston, Texas, USA

^**^ GuangDong Institute for Monitoring Laboratory Animals, China

^§^ Medical School, University of Texas Health Science Center at Houston, Houston, Texas, USA

^§§^ Laboratory for Conservation and Utilization of Bio-resources, Yunnan University, China

Supplementary information – Tables S1-S6

Supplementary information – Tables S1-S6

Table S1. The list of genes in carbohydrate catabolic process (totally 144 genes).

| **ID** | **Symbol** | **Gene full name** | **Location** |
| --- | --- | --- | --- |
| 1 | ADPGK | ADP-dependent glucokinase | 15q24.1 |
| 2 | ADRA1B | adrenergic, alpha-1B-, receptor | 5q33.3 |
| 3 | AGL | amylo-alpha-1, 6-glucosidase, 4-alpha-glucanotransferase | 1p21 |
| 4 | AKR1A1 | aldo-keto reductase family 1, member A1 (aldehyde reductase) | 1p33-p32 |
| 5 | ALDOA | aldolase A, fructose-bisphosphate | 16p11.2 |
| 6 | ALDOB | aldolase B, fructose-bisphosphate | 9q21.3-q22.2 |
| 7 | ALDOC | aldolase C, fructose-bisphosphate | 17cen-q12 |
| 8 | AMY2A | amylase, alpha 2A (pancreatic) | 1p21 |
| 9 | ARNT | aryl hydrocarbon receptor nuclear translocator | 1q21 |
| 10 | BAD | BCL2-associated agonist of cell death | 11q13.1 |
| 11 | BPGM | 2,3-bisphosphoglycerate mutase | 7q31-q34 |
| 12 | CALM3 | calmodulin 3 (phosphorylase kinase, delta) | 19q13.2-q13.3 |
| 13 | CHI3L1 | chitinase 3-like 1 (cartilage glycoprotein-39) | 1q32.1 |
| 14 | CHI3L2 | chitinase 3-like 2 | 1p13.3 |
| 15 | CHIA | chitinase, acidic | 1p13.2 |
| 16 | CHID1 | chitinase domain containing 1 | 11p15.5 |
| 17 | CHIT1 | chitinase 1 (chitotriosidase) | 1q31-q32 |
| 18 | CPS1 | carbamoyl-phosphate synthase 1, mitochondrial | 2q35 |
| 19 | CTBS | chitobiase, di-N-acetyl- | 1p22 |
| 20 | DHDH | dihydrodiol dehydrogenase (dimeric) | 19q13.3 |
| 21 | DHTKD1 | dehydrogenase E1 and transketolase domain containing 1 | 10p14 |
| 22 | DLAT | dihydrolipoamide S-acetyltransferase | 11q23.1 |
| 23 | ECD | ecdysoneless homolog (Drosophila) | 10q22.3 |
| 24 | ENO1 | enolase 1, (alpha) | 1p36.2 |
| 25 | ENO2 | enolase 2 (gamma, neuronal) | 12p13 |
| 26 | ENO3 | enolase 3 (beta, muscle) | 17pter-p11 |
| 27 | ENO4 | enolase family member 4 | 10q25.3 |
| 28 | ENTPD5 | ectonucleoside triphosphate diphosphohydrolase 5 | 14q24 |
| 29 | FUCA1 | fucosidase, alpha-L- 1, tissue | 1p34 |
| 30 | FUT1 | fucosyltransferase 1 (galactoside 2-alpha-L-fucosyltransferase, H blood group) | 19q13.3 |
| 31 | FUT10 | fucosyltransferase 10 (alpha (1,3) fucosyltransferase) | 8p12 |
| 32 | FUT2 | fucosyltransferase 2 (secretor status included) | 19q13.3 |
| 33 | FUT4 | fucosyltransferase 4 (alpha (1,3) fucosyltransferase, myeloid-specific) | 11q21 |
| 34 | FUT5 | fucosyltransferase 5 (alpha (1,3) fucosyltransferase) | 19p13.3 |
| 35 | FUT6 | fucosyltransferase 6 (alpha (1,3) fucosyltransferase) | 19p13.3 |
| 36 | FUT7 | fucosyltransferase 7 (alpha (1,3) fucosyltransferase) | 9q34.3 |
| 37 | FUT8 | fucosyltransferase 8 (alpha (1,6) fucosyltransferase) | 14q24.3 |
| 38 | FUT9 | fucosyltransferase 9 (alpha (1,3) fucosyltransferase) | 6q16 |
| 39 | G6PC | glucose-6-phosphatase, catalytic subunit | 17q21 |
| 40 | G6PD | glucose-6-phosphate dehydrogenase | Xq28 |
| 41 | GAA | glucosidase, alpha; acid | 17q25.2-q25.3 |
| 42 | GALE | UDP-galactose-4-epimerase | 1p36-p35 |
| 43 | GALK1 | galactokinase 1 | 17q24 |
| 44 | GALT | galactose-1-phosphate uridylyltransferase | 9p13 |
| 45 | GAPDH | glyceraldehyde-3-phosphate dehydrogenase | 12p13 |
| 46 | GAPDHS | glyceraldehyde-3-phosphate dehydrogenase, spermatogenic | 19q13.12 |
| 47 | GBA2 | glucosidase, beta (bile acid) 2 | 9p13.3 |
| 48 | GBA3 | glucosidase, beta, acid 3 (cytosolic) | 4p15.2 |
| 49 | GCK | glucokinase (hexokinase 4) | 7p15.3-p15.1 |
| 50 | GLYR1 | glyoxylate reductase 1 homolog (Arabidopsis) | 16p13.3 |
| 51 | GM2A | GM2 ganglioside activator | 5q33.1 |
| 52 | GNPDA1 | glucosamine-6-phosphate deaminase 1 | 5q21 |
| 53 | GNS | glucosamine (N-acetyl)-6-sulfatase | 12q14 |
| 54 | GPD1 | glycerol-3-phosphate dehydrogenase 1 (soluble) | 12q12-q13 |
| 55 | GPD1L | glycerol-3-phosphate dehydrogenase 1-like | 3p22.3 |
| 56 | GPI | glucose-6-phosphate isomerase | 19q13.1 |
| 57 | GSK3A | glycogen synthase kinase 3 alpha | 19q13.2 |
| 58 | GUSB | glucuronidase, beta | 7q21.11 |
| 59 | GYG1 | glycogenin 1 | 3q24-q25.1 |
| 60 | GYG2 | glycogenin 2 | Xp22.3 |
| 61 | H6PD | hexose-6-phosphate dehydrogenase (glucose 1-dehydrogenase) | 1p36 |
| 62 | HDAC4 | histone deacetylase 4 | 2q37.3 |
| 63 | HEXB | hexosaminidase B (beta polypeptide) | 5q13 |
| 64 | HIBADH | 3-hydroxyisobutyrate dehydrogenase | 7p15.2 |
| 65 | HIF1A | hypoxia inducible factor 1, alpha subunit (basic helix-loop-helix transcription factor) | 14q23.2 |
| 66 | HK1 | hexokinase 1 | 10q22 |
| 67 | HK2 | hexokinase 2 | 2p13 |
| 68 | HK3 | hexokinase 3 (white cell) | 5q35.2 |
| 69 | HKDC1 | hexokinase domain containing 1 | 10q22.1 |
| 70 | HYAL1 | hyaluronoglucosaminidase 1 | 3p21.3-p21.2 |
| 71 | HYAL2 | hyaluronoglucosaminidase 2 | 3p21.3 |
| 72 | HYAL4 | hyaluronoglucosaminidase 4 | 7q31.3 |
| 73 | IGF1 | insulin-like growth factor 1 (somatomedin C) | 12q23.2 |
| 74 | INS | insulin | 11p15.5 |
| 75 | INSR | insulin receptor | 19p13.3-p13.2 |
| 76 | KHK | ketohexokinase (fructokinase) | 2p23.3 |
| 77 | KIF1B | kinesin family member 1B | 1p36.2 |
| 78 | LDHA | lactate dehydrogenase A | 11p15.4 |
| 79 | LDHAL6A | lactate dehydrogenase A-like 6A | 11p15.1 |
| 80 | LDHAL6B | lactate dehydrogenase A-like 6B | 15q22.2 |
| 81 | LDHB | lactate dehydrogenase B | 12p12.2-p12.1 |
| 82 | LDHC | lactate dehydrogenase C | 11p15.1 |
| 83 | LRP5 | low density lipoprotein receptor-related protein 5 | 11q13.4 |
| 84 | LYG1 | lysozyme G-like 1 | 2q11.2 |
| 85 | LYG2 | lysozyme G-like 2 | 2q11.2 |
| 86 | LYVE1 | lymphatic vessel endothelial hyaluronan receptor 1 | 11p15 |
| 87 | MGAM | maltase-glucoamylase (alpha-glucosidase) | 7q34 |
| 88 | MIOX | myo-inositol oxygenase | 22q13.3 |
| 89 | MLXIPL | MLX interacting protein-like | 7q11.23 |
| 90 | NAGA | N-acetylgalactosaminidase, alpha- | 22q13-qter|22q11 |
| 91 | NCOR1 | nuclear receptor corepressor 1 | 17p11.2 |
| 92 | NUDT5 | nudix (nucleoside diphosphate linked moiety X)-type motif 5 | 10p14 |
| 93 | OGDH | oxoglutarate (alpha-ketoglutarate) dehydrogenase (lipoamide) | 7p14-p13 |
| 94 | OGDHL | oxoglutarate dehydrogenase-like | 10q11.23 |
| 95 | OVGP1 | oviductal glycoprotein 1, 120kDa | 1p13 |
| 96 | PDHA1 | pyruvate dehydrogenase (lipoamide) alpha 1 | Xp22.1 |
| 97 | PDHA2 | pyruvate dehydrogenase (lipoamide) alpha 2 | 4q22-q23 |
| 98 | PDHB | pyruvate dehydrogenase (lipoamide) beta | 3p21.1-p14.2 |
| 99 | PFKFB1 | 6-phosphofructo-2-kinase/fructose-2,6-biphosphatase 1 | Xp11.21 |
| 100 | PFKFB2 | 6-phosphofructo-2-kinase/fructose-2,6-biphosphatase 2 | 1q31 |
| 101 | PFKFB3 | 6-phosphofructo-2-kinase/fructose-2,6-biphosphatase 3 | 10p15.1 |
| 102 | PFKFB4 | 6-phosphofructo-2-kinase/fructose-2,6-biphosphatase 4 | 3p22-p21 |
| 103 | PFKL | phosphofructokinase, liver | 21q22.3 |
| 104 | PFKM | phosphofructokinase, muscle | 12q13.3 |
| 105 | PFKP | phosphofructokinase, platelet | 10p15.3-p15.2 |
| 106 | PGAM1 | phosphoglycerate mutase 1 (brain) | 10q25.3 |
| 107 | PGAM2 | phosphoglycerate mutase 2 (muscle) | 7p13-p12 |
| 108 | PGAM4 | phosphoglycerate mutase family member 4 | Xq13 |
| 109 | PGD | phosphogluconate dehydrogenase | 1p36.3-p36.13 |
| 110 | PGK1 | phosphoglycerate kinase 1 | Xq13 |
| 111 | PGK2 | phosphoglycerate kinase 2 | 6p12.3 |
| 112 | PGLS | 6-phosphogluconolactonase | 19p13.2 |
| 113 | PGLYRP1 | peptidoglycan recognition protein 1 | 19q13.2-q13.3 |
| 114 | PGLYRP2 | peptidoglycan recognition protein 2 | 19p13.12 |
| 115 | PGLYRP3 | peptidoglycan recognition protein 3 | 1q21 |
| 116 | PGLYRP4 | peptidoglycan recognition protein 4 | 1q21 |
| 117 | PGM1 | phosphoglucomutase 1 | 1p31 |
| 118 | PGM2 | phosphoglucomutase 2 | 4p14 |
| 119 | PHKA1 | phosphorylase kinase, alpha 1 (muscle) | Xq12-q13 |
| 120 | PHKA2 | phosphorylase kinase, alpha 2 (liver) | Xp22.2-p22.1 |
| 121 | PHKB | phosphorylase kinase, beta | 16q12-q13 |
| 122 | PHKG1 | phosphorylase kinase, gamma 1 (muscle) | 7p11.2 |
| 123 | PHKG2 | phosphorylase kinase, gamma 2 (testis) | 16p11.2 |
| 124 | PKLR | pyruvate kinase, liver and RBC | 1q21 |
| 125 | PKM2 | pyruvate kinase, muscle | 15q22 |
| 126 | PPARA | peroxisome proliferator-activated receptor alpha | 22q12-q13.1|22q13.31 |
| 127 | PPP1CB | protein phosphatase 1, catalytic subunit, beta isozyme | 2p23 |
| 128 | PPP1R3C | protein phosphatase 1, regulatory (inhibitor) subunit 3C | 10q23-q24 |
| 129 | PPP1R3E | protein phosphatase 1, regulatory (inhibitor) subunit 3E | 14q11.2 |
| 130 | PRKAG1 | protein kinase, AMP-activated, gamma 1 non-catalytic subunit | 12q12-q14 |
| 131 | PRKAG2 | protein kinase, AMP-activated, gamma 2 non-catalytic subunit | 7q36.1 |
| 132 | PYGB | phosphorylase, glycogen; brain | 20p11.2-p11.1 |
| 133 | PYGL | phosphorylase, glycogen, liver | 14q21-q22 |
| 134 | PYGM | phosphorylase, glycogen, muscle | 11q12-q13.2 |
| 135 | RPE | ribulose-5-phosphate-3-epimerase | 2q32-q33.3 |
| 136 | RPIA | ribose 5-phosphate isomerase A | 2p11.2 |
| 137 | SORD | sorbitol dehydrogenase | 15q15.3 |
| 138 | SPACA3 | sperm acrosome associated 3 | 17q11.2 |
| 139 | TALDO1 | transaldolase 1 | 11p15.5-p15.4 |
| 140 | TKT | transketolase | 3p14.3 |
| 141 | TKTL1 | transketolase-like 1 | Xq28 |
| 142 | TPI1 | triosephosphate isomerase 1 | 12p13 |
| 143 | TREH | trehalase (brush-border membrane glycoprotein) | 11q23.3 |
| 144 | XYLB | xylulokinase homolog (H. influenzae) | 3p22-p21.3 |

Table S2. The list of genes in carbohydrate biosynthetic process (totally 185 genes).

| **ID** | **Symbol** | **Gene full name** | **Location** |
| --- | --- | --- | --- |
| 1 | ACN9 | ACN9 homolog | 7q21.3 |
| 2 | ADCYAP1R1 | adenylate cyclase activating polypeptide 1 (pituitary) receptor type I | 7p14 |
| 3 | ADIPOQ | adiponectin, C1Q and collagen domain containing | 3q27 |
| 4 | AGL | amylo-alpha-1, 6-glucosidase, 4-alpha-glucanotransferase | 1p21 |
| 5 | AKT1 | v-akt murine thymoma viral oncogene homolog 1 | 14q32.32|14q32.32 |
| 6 | AKT2 | v-akt murine thymoma viral oncogene homolog 2 | 19q13.1-q13.2 |
| 7 | ALDOA | aldolase A, fructose-bisphosphate | 16p11.2 |
| 8 | ALDOB | aldolase B, fructose-bisphosphate | 9q21.3-q22.2 |
| 9 | ALDOC | aldolase C, fructose-bisphosphate | 17cen-q12 |
| 10 | ALG1 | asparagine-linked glycosylation 1, beta-1,4-mannosyltransferase homolog (S. cerevisiae) | 16p13.3 |
| 11 | ALG10 | asparagine-linked glycosylation 10, alpha-1,2-glucosyltransferase homolog (S. pombe) | 12p11.1 |
| 12 | ALG10B | asparagine-linked glycosylation 10, alpha-1,2-glucosyltransferase homolog B (yeast) | 12q12 |
| 13 | ALG11 | asparagine-linked glycosylation 11, alpha-1,2-mannosyltransferase homolog (yeast) | 13q14.2 |
| 14 | ALG12 | asparagine-linked glycosylation 12, alpha-1,6-mannosyltransferase homolog (S. cerevisiae) | 22q13.33 |
| 15 | ALG13 | asparagine-linked glycosylation 13 homolog (S. cerevisiae) | Xq23 |
| 16 | ALG14 | asparagine-linked glycosylation 14 homolog (S. cerevisiae) | 1p21.3 |
| 17 | ALG2 | asparagine-linked glycosylation 2, alpha-1,3-mannosyltransferase homolog (S. cerevisiae) | 9q22.33 |
| 18 | ALG3 | asparagine-linked glycosylation 3, alpha-1,3- mannosyltransferase homolog (S. cerevisiae) | 3q27.1 |
| 19 | ALG5 | asparagine-linked glycosylation 5, dolichyl-phosphate beta-glucosyltransferase homolog (S. cerevisiae) | 13q13.3 |
| 20 | ALG6 | asparagine-linked glycosylation 6, alpha-1,3-glucosyltransferase homolog (S. cerevisiae) | 1p31.3 |
| 21 | ALG8 | asparagine-linked glycosylation 8, alpha-1,3-glucosyltransferase homolog (S. cerevisiae) | 11q14.1 |
| 22 | ALG9 | asparagine-linked glycosylation 9, alpha-1,2-mannosyltransferase homolog (S. cerevisiae) | 11q23 |
| 23 | ANGPT1 | angiopoietin 1 | 8q23.1 |
| 24 | ARPP19 | cAMP-regulated phosphoprotein, 19kDa | 15q21.2 |
| 25 | ATF3 | activating transcription factor 3 | 1q32.3 |
| 26 | ATF4 | activating transcription factor 4 (tax-responsive enhancer element B67) | 22q13.1 |
| 27 | B3GALNT1 | beta-1,3-N-acetylgalactosaminyltransferase 1 (globoside blood group) | 3q25 |
| 28 | B3GALT1 | UDP-Gal:betaGlcNAc beta 1,3-galactosyltransferase, polypeptide 1 | 2q24.3 |
| 29 | B3GALT2 | UDP-Gal:betaGlcNAc beta 1,3-galactosyltransferase, polypeptide 2 | 1q31 |
| 30 | B3GALT6 | UDP-Gal:betaGal beta 1,3-galactosyltransferase polypeptide 6 | 1p36.33 |
| 31 | B3GAT2 | beta-1,3-glucuronyltransferase 2 (glucuronosyltransferase S) | 6q13 |
| 32 | B3GAT3 | beta-1,3-glucuronyltransferase 3 (glucuronosyltransferase I) | 11q12.3 |
| 33 | B3GNT1 | UDP-GlcNAc:betaGal beta-1,3-N-acetylglucosaminyltransferase 1 | 11q13.2 |
| 34 | B3GNT8 | UDP-GlcNAc:betaGal beta-1,3-N-acetylglucosaminyltransferase 8 | 19q13.2 |
| 35 | B4GALT1 | UDP-Gal:betaGlcNAc beta 1,4- galactosyltransferase, polypeptide 1 | 9p13 |
| 36 | B4GALT7 | xylosylprotein beta 1,4-galactosyltransferase, polypeptide 7 (galactosyltransferase I) | 5q35.2-q35.3 |
| 37 | C1QTNF2 | C1q and tumor necrosis factor related protein 2 | 5q33.3 |
| 38 | CERCAM | cerebral endothelial cell adhesion molecule | 9q34.11 |
| 39 | CHST10 | carbohydrate sulfotransferase 10 | 2q11.2 |
| 40 | CHST11 | carbohydrate (chondroitin 4) sulfotransferase 11 | 12q |
| 41 | CHST12 | carbohydrate (chondroitin 4) sulfotransferase 12 | 7p22 |
| 42 | CHST13 | carbohydrate (chondroitin 4) sulfotransferase 13 | 3q21.3 |
| 43 | CHST14 | carbohydrate (N-acetylgalactosamine 4-0) sulfotransferase 14 | 15q15.1 |
| 44 | CHST15 | carbohydrate (N-acetylgalactosamine 4-sulfate 6-O) sulfotransferase 15 | 10q26 |
| 45 | CHST3 | carbohydrate (chondroitin 6) sulfotransferase 3 | 10q22.1 |
| 46 | CHST6 | carbohydrate (N-acetylglucosamine 6-O) sulfotransferase 6 | 16q22 |
| 47 | CHST7 | carbohydrate (N-acetylglucosamine 6-O) sulfotransferase 7 | Xp11.23 |
| 48 | CHST8 | carbohydrate (N-acetylgalactosamine 4-0) sulfotransferase 8 | 19q13.1 |
| 49 | CHST9 | carbohydrate (N-acetylgalactosamine 4-0) sulfotransferase 9 | 18q11.2 |
| 50 | CHSY1 | chondroitin sulfate synthase 1 | 15q26.3 |
| 51 | CMAS | cytidine monophosphate N-acetylneuraminic acid synthetase | 12p12.1 |
| 52 | COG2 | component of oligomeric golgi complex 2 | 1q42.2 |
| 53 | CSGALNACT1 | chondroitin sulfate N-acetylgalactosaminyltransferase 1 | 8p21.3 |
| 54 | CSGALNACT2 | chondroitin sulfate N-acetylgalactosaminyltransferase 2 | 10q11.21 |
| 55 | DOLK | dolichol kinase | 9q34.11 |
| 56 | DOLPP1 | dolichyl pyrophosphate phosphatase 1 | 9q34.1 |
| 57 | DPAGT1 | dolichyl-phosphate (UDP-N-acetylglucosamine) N-acetylglucosaminephosphotransferase 1 (GlcNAc-1-P transferase) | 11q23.3 |
| 58 | DPM1 | dolichyl-phosphate mannosyltransferase polypeptide 1, catalytic subunit | 20q13.13 |
| 59 | DPM2 | dolichyl-phosphate mannosyltransferase polypeptide 2, regulatory subunit | 9q34.13 |
| 60 | DPM3 | dolichyl-phosphate mannosyltransferase polypeptide 3 | 1q22 |
| 61 | DSE | dermatan sulfate epimerase | 6q22 |
| 62 | DYRK2 | dual-specificity tyrosine-(Y)-phosphorylation regulated kinase 2 | 12q15 |
| 63 | ENO1 | enolase 1, (alpha) | 1p36.2 |
| 64 | ENO2 | enolase 2 (gamma, neuronal) | 12p13 |
| 65 | ENO3 | enolase 3 (beta, muscle) | 17pter-p11 |
| 66 | ENPP1 | ectonucleotide pyrophosphatase/phosphodiesterase 1 | 6q22-q23 |
| 67 | EXT1 | exostosin 1 | 8q24.11 |
| 68 | EXT2 | exostosin 2 | 11p12-p11 |
| 69 | FBP1 | fructose-1,6-bisphosphatase 1 | 9q22.3 |
| 70 | FBP2 | fructose-1,6-bisphosphatase 2 | 9q22.3 |
| 71 | FOXO1 | forkhead box O1 | 13q14.1 |
| 72 | FUT8 | fucosyltransferase 8 (alpha (1,6) fucosyltransferase) | 14q24.3 |
| 73 | G6PC | glucose-6-phosphatase, catalytic subunit | 17q21 |
| 74 | G6PC2 | glucose-6-phosphatase, catalytic, 2 | 2q24.3 |
| 75 | G6PC3 | glucose 6 phosphatase, catalytic, 3 | 17q21.31 |
| 76 | G6PD | glucose-6-phosphate dehydrogenase | Xq28 |
| 77 | GALNT5 | UDP-N-acetyl-alpha-D-galactosamine:polypeptide N-acetylgalactosaminyltransferase 5 (GalNAc-T5) | 2q24.1 |
| 78 | GAPDH | glyceraldehyde-3-phosphate dehydrogenase | 12p13 |
| 79 | GAPDHS | glyceraldehyde-3-phosphate dehydrogenase, spermatogenic | 19q13.12 |
| 80 | GBE1 | glucan (1,4-alpha-), branching enzyme 1 | 3p12.3 |
| 81 | GCK | glucokinase (hexokinase 4) | 7p15.3-p15.1 |
| 82 | GCNT2 | glucosaminyl (N-acetyl) transferase 2, I-branching enzyme (I blood group) | 6p24.2 |
| 83 | GFPT1 | glutamine--fructose-6-phosphate transaminase 1 | 2p13 |
| 84 | GFPT2 | glutamine-fructose-6-phosphate transaminase 2 | 5q34-q35 |
| 85 | GLCE | glucuronic acid epimerase | 15q23 |
| 86 | GLT25D1 | glycosyltransferase 25 domain containing 1 | 19p13.11 |
| 87 | GLT25D2 | glycosyltransferase 25 domain containing 2 | 1q25 |
| 88 | GMDS | GDP-mannose 4,6-dehydratase | 6p25 |
| 89 | GMPPA | GDP-mannose pyrophosphorylase A | 2q35 |
| 90 | GMPPB | GDP-mannose pyrophosphorylase B | 3p21.31 |
| 91 | GNE | glucosamine (UDP-N-acetyl)-2-epimerase/N-acetylmannosamine kinase | 9p13.3 |
| 92 | GNMT | glycine N-methyltransferase | 6p12 |
| 93 | GNPNAT1 | glucosamine-phosphate N-acetyltransferase 1 | 14q22.1 |
| 94 | GOT1 | glutamic-oxaloacetic transaminase 1, soluble (aspartate aminotransferase 1) | 10q24.1-q25.1 |
| 95 | GOT2 | glutamic-oxaloacetic transaminase 2, mitochondrial (aspartate aminotransferase 2) | 16q21 |
| 96 | GPD1 | glycerol-3-phosphate dehydrogenase 1 (soluble) | 12q12-q13 |
| 97 | GPD2 | glycerol-3-phosphate dehydrogenase 2 (mitochondrial) | 2q24.1 |
| 98 | GPI | glucose-6-phosphate isomerase | 19q13.1 |
| 99 | GPT | glutamic-pyruvate transaminase (alanine aminotransferase) | 8q24.3 |
| 100 | GRB10 | growth factor receptor-bound protein 10 | 7p12.2 |
| 101 | GYG1 | glycogenin 1 | 3q24-q25.1 |
| 102 | GYG2 | glycogenin 2 | Xp22.3 |
| 103 | GYS1 | glycogen synthase 1 (muscle) | 19q13.3 |
| 104 | GYS2 | glycogen synthase 2 (liver) | 12p12.2 |
| 105 | HAS1 | hyaluronan synthase 1 | 19q13.4 |
| 106 | HS2ST1 | heparan sulfate 2-O-sulfotransferase 1 | 1p22.3 |
| 107 | HS6ST1 | heparan sulfate 6-O-sulfotransferase 1 | 2q21 |
| 108 | HS6ST2 | heparan sulfate 6-O-sulfotransferase 2 | Xq26.2 |
| 109 | HS6ST3 | heparan sulfate 6-O-sulfotransferase 3 | 13q32.1 |
| 110 | IGF1 | insulin-like growth factor 1 (somatomedin C) | 12q23.2 |
| 111 | IL6 | interleukin 6 (interferon, beta 2) | 7p21 |
| 112 | INS | insulin | 11p15.5 |
| 113 | INSR | insulin receptor | 19p13.3-p13.2 |
| 114 | IRS1 | insulin receptor substrate 1 | 2q36 |
| 115 | IRS2 | insulin receptor substrate 2 | 13q34 |
| 116 | ISYNA1 | inositol-3-phosphate synthase 1 | 19p13.11 |
| 117 | LALBA | lactalbumin, alpha- | 12q13 |
| 118 | LEP | leptin | 7q31.3 |
| 119 | MAS1 | MAS1 oncogene | 6q25.3-q26 |
| 120 | MAT2B | methionine adenosyltransferase II, beta | 5q34-q35 |
| 121 | MDH1 | malate dehydrogenase 1, NAD (soluble) | 2p13.3 |
| 122 | MDH2 | malate dehydrogenase 2, NAD (mitochondrial) | 7cen-q22 |
| 123 | MGAT2 | mannosyl (alpha-1,6-)-glycoprotein beta-1,2-N-acetylglucosaminyltransferase | 14q21 |
| 124 | MPDU1 | mannose-P-dolichol utilization defect 1 | 17p13.1-p12 |
| 125 | MPI | mannose phosphate isomerase | 15q22-qter |
| 126 | MTOR | mechanistic target of rapamycin (serine/threonine kinase) | 1p36.2 |
| 127 | NANP | N-acetylneuraminic acid phosphatase | 20p11.1 |
| 128 | NANS | N-acetylneuraminic acid synthase | 9p24.1-p23 |
| 129 | NDST1 | N-deacetylase/N-sulfotransferase (heparan glucosaminyl) 1 | 5q33.1 |
| 130 | NR3C1 | nuclear receptor subfamily 3, group C, member 1 (glucocorticoid receptor) | 5q31.3 |
| 131 | P2RY1 | purinergic receptor P2Y, G-protein coupled, 1 | 3q25.2 |
| 132 | PC | pyruvate carboxylase | 11q13.4-q13.5 |
| 133 | PCK1 | phosphoenolpyruvate carboxykinase 1 (soluble) | 20q13.31 |
| 134 | PCK2 | phosphoenolpyruvate carboxykinase 2 (mitochondrial) | 14q11.2 |
| 135 | PFKFB1 | 6-phosphofructo-2-kinase/fructose-2,6-biphosphatase 1 | Xp11.21 |
| 136 | PGAM1 | phosphoglycerate mutase 1 (brain) | 10q25.3 |
| 137 | PGAM2 | phosphoglycerate mutase 2 (muscle) | 7p13-p12 |
| 138 | PGD | phosphogluconate dehydrogenase | 1p36.3-p36.13 |
| 139 | PGK1 | phosphoglycerate kinase 1 | Xq13 |
| 140 | PGM1 | phosphoglucomutase 1 | 1p31 |
| 141 | PGM2 | phosphoglucomutase 2 | 4p14 |
| 142 | PGM3 | phosphoglucomutase 3 | 6q14.1-q15 |
| 143 | PHKG1 | phosphorylase kinase, gamma 1 (muscle) | 7p11.2 |
| 144 | PHKG2 | phosphorylase kinase, gamma 2 (testis) | 16p11.2 |
| 145 | PLEK | pleckstrin | 2p13.3 |
| 146 | PMM1 | phosphomannomutase 1 | 22q13.2 |
| 147 | PMM2 | phosphomannomutase 2 | 16p13 |
| 148 | PPARGC1A | peroxisome proliferator-activated receptor gamma, coactivator 1 alpha | 4p15.1 |
| 149 | PPP1CB | protein phosphatase 1, catalytic subunit, beta isozyme | 2p23 |
| 150 | PPP1R3C | protein phosphatase 1, regulatory (inhibitor) subunit 3C | 10q23-q24 |
| 151 | PPP1R3E | protein phosphatase 1, regulatory (inhibitor) subunit 3E | 14q11.2 |
| 152 | PRKACA | protein kinase, cAMP-dependent, catalytic, alpha | 19p13.1 |
| 153 | PRKACB | protein kinase, cAMP-dependent, catalytic, beta | 1p36.1 |
| 154 | PRKACG | protein kinase, cAMP-dependent, catalytic, gamma | 9q13 |
| 155 | PRPS1 | phosphoribosyl pyrophosphate synthetase 1 | Xq21.32-q24 |
| 156 | PTH | parathyroid hormone | 11p15.3-p15.1 |
| 157 | PYGL | phosphorylase, glycogen, liver | 14q21-q22 |
| 158 | RBP4 | retinol binding protein 4, plasma | 10q23-q24 |
| 159 | RFT1 | RFT1 homolog (S. cerevisiae) | 3p21.1 |
| 160 | SDS | serine dehydratase | 12q24.13 |
| 161 | SELS | - | 15q26.3 |
| 162 | SLC25A1 | solute carrier family 25 (mitochondrial carrier; citrate transporter), member 1 | 22q11.21 |
| 163 | SLC25A10 | solute carrier family 25 (mitochondrial carrier; dicarboxylate transporter), member 10 | 17q25.3 |
| 164 | SLC25A11 | solute carrier family 25 (mitochondrial carrier; oxoglutarate carrier), member 11 | 17p13.3 |
| 165 | SLC25A12 | solute carrier family 25 (mitochondrial carrier, Aralar), member 12 | 2q24 |
| 166 | SLC25A13 | solute carrier family 25, member 13 (citrin) | 7q21.3 |
| 167 | SLC35D1 | solute carrier family 35 (UDP-glucuronic acid/UDP-N-acetylgalactosamine dual transporter), member D1 | 1p32-p31 |
| 168 | SNCA | synuclein, alpha (non A4 component of amyloid precursor) | 4q21 |
| 169 | SORBS1 | sorbin and SH3 domain containing 1 | 10q23.33 |
| 170 | SORD | sorbitol dehydrogenase | 15q15.3 |
| 171 | SRD5A3 | steroid 5 alpha-reductase 3 | 4q12 |
| 172 | ST8SIA2 | ST8 alpha-N-acetyl-neuraminide alpha-2,8-sialyltransferase 2 | 15q26 |
| 173 | ST8SIA6 | ST8 alpha-N-acetyl-neuraminide alpha-2,8-sialyltransferase 6 | 10p12.33 |
| 174 | TALDO1 | transaldolase 1 | 11p15.5-p15.4 |
| 175 | TKT | transketolase | 3p14.3 |
| 176 | TP53RK | TP53 regulating kinase | 20q13.2 |
| 177 | TPI1 | triosephosphate isomerase 1 | 12p13 |
| 178 | TSTA3 | tissue specific transplantation antigen P35B | 8q24.3 |
| 179 | UAP1 | UDP-N-acteylglucosamine pyrophosphorylase 1 | 1q23.3 |
| 180 | UGDH | UDP-glucose 6-dehydrogenase | 4p15.1 |
| 181 | UGP2 | UDP-glucose pyrophosphorylase 2 | 2p14-p13 |
| 182 | UST | uronyl-2-sulfotransferase | 6q25.1 |
| 183 | UXS1 | UDP-glucuronate decarboxylase 1 | 2q12.2 |
| 184 | XYLT1 | xylosyltransferase I | 16p12.3 |
| 185 | XYLT2 | xylosyltransferase II | 17q21.33 |

Table S3. The list of genes in lipid catabolic process (totally 218 genes).

| **ID** | **Symbol** | **Gene full name** | **Location** |
| --- | --- | --- | --- |
| 1 | AADAC | arylacetamide deacetylase (esterase) | 3q21.3-q25.2 |
| 2 | ABCD1 | ATP-binding cassette, sub-family D (ALD), member 1 | Xq28 |
| 3 | ABHD4 | abhydrolase domain containing 4 | 14q11.2 |
| 4 | ABHD5 | abhydrolase domain containing 5 | 3p21 |
| 5 | ACAA1 | acetyl-CoA acyltransferase 1 | 3p23-p22 |
| 6 | ACADL | acyl-CoA dehydrogenase, long chain | 2q34-q35 |
| 7 | ACADM | acyl-CoA dehydrogenase, C-4 to C-12 straight chain | 1p31 |
| 8 | ACADS | acyl-CoA dehydrogenase, C-2 to C-3 short chain | 12q24.31 |
| 9 | ACADVL | acyl-CoA dehydrogenase, very long chain | 17p13.1 |
| 10 | ACER1 | alkaline ceramidase 1 | 19p13.3 |
| 11 | ACOT7 | acyl-CoA thioesterase 7 | 1p36 |
| 12 | ACOT8 | acyl-CoA thioesterase 8 | 20q13.12 |
| 13 | ACOX1 | acyl-CoA oxidase 1, palmitoyl | 17q24-q25|17q25.1 |
| 14 | ACOX2 | acyl-CoA oxidase 2, branched chain | 3p14.3 |
| 15 | ACOX3 | acyl-CoA oxidase 3, pristanoyl | 4p15.3 |
| 16 | ACOXL | acyl-CoA oxidase-like | 2q13 |
| 17 | ACSL5 | acyl-CoA synthetase long-chain family member 5 | 10q25.1-q25.2 |
| 18 | ADIPOQ | adiponectin, C1Q and collagen domain containing | 3q27 |
| 19 | ADORA1 | adenosine A1 receptor | 1q32.1 |
| 20 | ADRA2A | adrenergic, alpha-2A-, receptor | 10q24-q26 |
| 21 | AKR1D1 | aldo-keto reductase family 1, member D1 (delta 4-3-ketosteroid-5-beta-reductase) | 7q32-q33 |
| 22 | AKT1 | v-akt murine thymoma viral oncogene homolog 1 | 14q32.32|14q32.32 |
| 23 | AKT2 | v-akt murine thymoma viral oncogene homolog 2 | 19q13.1-q13.2 |
| 24 | AMACR | alpha-methylacyl-CoA racemase | 5p13 |
| 25 | ANGPTL3 | angiopoietin-like 3 | 1p31.1-p22.3 |
| 26 | APOA2 | apolipoprotein A-II | 1q21-q23 |
| 27 | APOA4 | apolipoprotein A-IV | 11q23 |
| 28 | APOA5 | apolipoprotein A-V | 11q23 |
| 29 | APOB | apolipoprotein B (including Ag(x) antigen) | 2p24-p23 |
| 30 | APOC1 | apolipoprotein C-I | 19q13.2 |
| 31 | APOC2 | apolipoprotein C-II | 19q13.2 |
| 32 | APOC3 | apolipoprotein C-III | 11q23.1-q23.2 |
| 33 | APOE | apolipoprotein E | 19q13.2 |
| 34 | ASPG | asparaginase homolog (S. cerevisiae) | 14q32.33 |
| 35 | AZGP1 | alpha-2-glycoprotein 1, zinc-binding | 7q22.1 |
| 36 | BDH2 | 3-hydroxybutyrate dehydrogenase, type 2 | 4q24 |
| 37 | C6orf126 | chromosome 6 open reading frame 126 | 6p21.31 |
| 38 | C6orf127 | chromosome 6 open reading frame 127 | 6p21.31 |
| 39 | CEL | carboxyl ester lipase (bile salt-stimulated lipase) | 9q34.3 |
| 40 | CIDEA | cell death-inducing DFFA-like effector a | 18p11.21|18 |
| 41 | CLC | Charcot-Leyden crystal protein | 19q13.1 |
| 42 | CLPS | colipase, pancreatic | 6pter-p21.1 |
| 43 | CNR1 | cannabinoid receptor 1 (brain) | 6q14-q15 |
| 44 | CPS1 | carbamoyl-phosphate synthase 1, mitochondrial | 2q35 |
| 45 | CPT1A | carnitine palmitoyltransferase 1A (liver) | 11q13.2 |
| 46 | CPT1B | carnitine palmitoyltransferase 1B (muscle) | 22q13.33 |
| 47 | CPT2 | carnitine palmitoyltransferase 2 | 1p32 |
| 48 | CRAT | carnitine O-acetyltransferase | 9q34.1 |
| 49 | CROT | carnitine O-octanoyltransferase | 7q21.1 |
| 50 | CYP1A2 | cytochrome P450, family 1, subfamily A, polypeptide 2 | 15q24.1 |
| 51 | CYP24A1 | cytochrome P450, family 24, subfamily A, polypeptide 1 | 20q13 |
| 52 | CYP39A1 | cytochrome P450, family 39, subfamily A, polypeptide 1 | 6p21.1-p11.2 |
| 53 | CYP3A4 | cytochrome P450, family 3, subfamily A, polypeptide 4 | 7q21.1 |
| 54 | CYP46A1 | cytochrome P450, family 46, subfamily A, polypeptide 1 | 14q32.1 |
| 55 | CYP7A1 | cytochrome P450, family 7, subfamily A, polypeptide 1 | 8q11-q12 |
| 56 | DAGLA | diacylglycerol lipase, alpha | 11q12.2 |
| 57 | DAGLB | diacylglycerol lipase, beta | 7p22.1 |
| 58 | DCI | dodecenoyl-CoA isomerase | 16p13.3 |
| 59 | DDHD1 | DDHD domain containing 1 | 14q21 |
| 60 | DDHD2 | DDHD domain containing 2 | 8p11.23 |
| 61 | DECR1 | 2,4-dienoyl CoA reductase 1, mitochondrial | 8q21.3 |
| 62 | DRD1 | dopamine receptor D1 | 5q35.1 |
| 63 | ECH1 | enoyl CoA hydratase 1, peroxisomal | 19q13.1 |
| 64 | ECHS1 | enoyl CoA hydratase, short chain, 1, mitochondrial | 10q26.2-q26.3 |
| 65 | ENPP2 | ectonucleotide pyrophosphatase/phosphodiesterase 2 | 8q24.1 |
| 66 | ENPP6 | ectonucleotide pyrophosphatase/phosphodiesterase 6 | 4q35.1 |
| 67 | ETFDH | electron-transferring-flavoprotein dehydrogenase | 4q32-q35 |
| 68 | FAAH | fatty acid amide hydrolase | 1p35-p34 |
| 69 | FABP1 | fatty acid binding protein 1, liver | 2p11 |
| 70 | FABP4 | fatty acid binding protein 4, adipocyte | 8q21 |
| 71 | FGF23 | fibroblast growth factor 23 | 12p13.3 |
| 72 | GALC | galactosylceramidase | 14q31 |
| 73 | GBA2 | glucosidase, beta (bile acid) 2 | 9p13.3 |
| 74 | GBA3 | glucosidase, beta, acid 3 (cytosolic) | 4p15.2 |
| 75 | GIMAP5 | GTPase, IMAP family member 5 | 7q36.1 |
| 76 | GLA | galactosidase, alpha | Xq22 |
| 77 | GM2A | GM2 ganglioside activator | 5q33.1 |
| 78 | GPR109A | G protein-coupled receptor 109A | 12q24.31 |
| 79 | HACL1 | 2-hydroxyacyl-CoA lyase 1 | 3p25.1 |
| 80 | HADH | hydroxyacyl-CoA dehydrogenase | 4q22-q26 |
| 81 | HADHA | hydroxyacyl-CoA dehydrogenase/3-ketoacyl-CoA thiolase/enoyl-CoA hydratase (trifunctional protein), alpha subunit | 2p23 |
| 82 | HADHB | hydroxyacyl-CoA dehydrogenase/3-ketoacyl-CoA thiolase/enoyl-CoA hydratase (trifunctional protein), beta subunit | 2p23 |
| 83 | HAO1 | hydroxyacid oxidase (glycolate oxidase) 1 | 20p12 |
| 84 | HAO2 | hydroxyacid oxidase 2 (long chain) | 1p13.3-p13.1 |
| 85 | HEXA | hexosaminidase A (alpha polypeptide) | 15q24.1 |
| 86 | HEXB | hexosaminidase B (beta polypeptide) | 5q13 |
| 87 | HSD17B11 | hydroxysteroid (17-beta) dehydrogenase 11 | 4q22.1 |
| 88 | HSD17B14 | hydroxysteroid (17-beta) dehydrogenase 14 | 19q13.33 |
| 89 | HSD17B4 | hydroxysteroid (17-beta) dehydrogenase 4 | 5q21 |
| 90 | HSD17B6 | hydroxysteroid (17-beta) dehydrogenase 6 homolog (mouse) | 12q13 |
| 91 | IAH1 | isoamyl acetate-hydrolyzing esterase 1 homolog (S. cerevisiae) | 2p25.1 |
| 92 | IL1B | interleukin 1, beta | 2q14 |
| 93 | INS | insulin | 11p15.5 |
| 94 | IRS1 | insulin receptor substrate 1 | 2q36 |
| 95 | IRS2 | insulin receptor substrate 2 | 13q34 |
| 96 | LEP | leptin | 7q31.3 |
| 97 | LGALS13 | lectin, galactoside-binding, soluble, 13 | 19q13.1 |
| 98 | LIPA | lipase A, lysosomal acid, cholesterol esterase | 10q23.2-q23.3 |
| 99 | LIPC | lipase, hepatic | 15q21-q23 |
| 100 | LIPE | lipase, hormone-sensitive | 19q13.2 |
| 101 | LIPF | lipase, gastric | 10q23.31 |
| 102 | LIPG | lipase, endothelial | 18q21.1 |
| 103 | LIPH | lipase, member H | 3q27 |
| 104 | LIPI | lipase, member I | 21q11.2 |
| 105 | LIPJ | lipase, family member J | 10q23.31 |
| 106 | LIPK | lipase, family member K | 10q23.31 |
| 107 | LIPM | lipase, family member M | 10q23.31 |
| 108 | LIPN | lipase, family member N | 10q23.31 |
| 109 | LPIN1 | lipin 1 | 2p25.1 |
| 110 | LPL | lipoprotein lipase | 8p22 |
| 111 | MCEE | methylmalonyl CoA epimerase | 2p13.3 |
| 112 | MGLL | monoglyceride lipase | 3q21.3 |
| 113 | MLYCD | malonyl-CoA decarboxylase | 16q24 |
| 114 | MTOR | mechanistic target of rapamycin (serine/threonine kinase) | 1p36.2 |
| 115 | MUT | methylmalonyl CoA mutase | 6p12.3 |
| 116 | NAGA | N-acetylgalactosaminidase, alpha- | 22q13-qter|22q11 |
| 117 | NAPEPLD | N-acyl phosphatidylethanolamine phospholipase D | 7q22.1 |
| 118 | NCEH1 | neutral cholesterol ester hydrolase 1 | 3q26.31 |
| 119 | NEU3 | sialidase 3 (membrane sialidase) | 11q13.5 |
| 120 | OC90 | otoconin 90 | 8q24.22 |
| 121 | PAFAH1B1 | platelet-activating factor acetylhydrolase 1b, regulatory subunit 1 (45kDa) | 17p13.3 |
| 122 | PAFAH1B2 | platelet-activating factor acetylhydrolase 1b, catalytic subunit 2 (30kDa) | 11q23 |
| 123 | PAFAH1B3 | platelet-activating factor acetylhydrolase 1b, catalytic subunit 3 (29kDa) | 19q13.1 |
| 124 | PAFAH2 | platelet-activating factor acetylhydrolase 2, 40kDa | 1p36 |
| 125 | PCCA | propionyl CoA carboxylase, alpha polypeptide | 13q32 |
| 126 | PCCB | propionyl CoA carboxylase, beta polypeptide | 3q21-q22 |
| 127 | PDE3B | phosphodiesterase 3B, cGMP-inhibited | 11p15.1 |
| 128 | PEX13 | peroxisomal biogenesis factor 13 | 2p16.1 |
| 129 | PEX7 | peroxisomal biogenesis factor 7 | 6q23.3 |
| 130 | PHYH | phytanoyl-CoA 2-hydroxylase | 10p13 |
| 131 | PLA1A | phospholipase A1 member A | 3q13.13-q13.2 |
| 132 | PLA2G10 | phospholipase A2, group X | 16p13.1-p12 |
| 133 | PLA2G12A | phospholipase A2, group XIIA | 4q25 |
| 134 | PLA2G12B | phospholipase A2, group XIIB | 10q22.1 |
| 135 | PLA2G15 | phospholipase A2, group XV | 16q22.1 |
| 136 | PLA2G16 | phospholipase A2, group XVI | 11q12.3 |
| 137 | PLA2G1B | phospholipase A2, group IB (pancreas) | 12q23-q24.1 |
| 138 | PLA2G2A | phospholipase A2, group IIA (platelets, synovial fluid) | 1p35 |
| 139 | PLA2G2C | phospholipase A2, group IIC | 1p36.12 |
| 140 | PLA2G2D | phospholipase A2, group IID | 1p36.12 |
| 141 | PLA2G2E | phospholipase A2, group IIE | 1p36.13 |
| 142 | PLA2G2F | phospholipase A2, group IIF | 1p35 |
| 143 | PLA2G3 | phospholipase A2, group III | 22q12.2 |
| 144 | PLA2G4A | phospholipase A2, group IVA (cytosolic, calcium-dependent) | 1q25 |
| 145 | PLA2G4B | phospholipase A2, group IVB (cytosolic) | 15q11.2-q21.3 |
| 146 | PLA2G4C | phospholipase A2, group IVC (cytosolic, calcium-independent) | 19q13.3 |
| 147 | PLA2G4D | phospholipase A2, group IVD (cytosolic) | 15q15.1 |
| 148 | PLA2G4E | phospholipase A2, group IVE | 15q15.1 |
| 149 | PLA2G4F | phospholipase A2, group IVF | 15q15.1 |
| 150 | PLA2G5 | phospholipase A2, group V | 1p36-p34 |
| 151 | PLA2G6 | phospholipase A2, group VI (cytosolic, calcium-independent) | 22q13.1 |
| 152 | PLA2G7 | phospholipase A2, group VII (platelet-activating factor acetylhydrolase, plasma) | 6p21.2-p12 |
| 153 | PLB1 | phospholipase B1 | 2p23.2 |
| 154 | PLBD1 | phospholipase B domain containing 1 | 12p13.1 |
| 155 | PLBD2 | phospholipase B domain containing 2 | 12q24.13 |
| 156 | PLCB1 | phospholipase C, beta 1 (phosphoinositide-specific) | 20p12 |
| 157 | PLCB2 | phospholipase C, beta 2 | 15q15 |
| 158 | PLCB3 | phospholipase C, beta 3 (phosphatidylinositol-specific) | 11q13 |
| 159 | PLCB4 | phospholipase C, beta 4 | 20p12 |
| 160 | PLCD1 | phospholipase C, delta 1 | 3p22-p21.3 |
| 161 | PLCD3 | phospholipase C, delta 3 | 17q21.31 |
| 162 | PLCD4 | phospholipase C, delta 4 | 2q35 |
| 163 | PLCE1 | phospholipase C, epsilon 1 | 10q23 |
| 164 | PLCG1 | phospholipase C, gamma 1 | 20q12-q13.1 |
| 165 | PLCG2 | phospholipase C, gamma 2 (phosphatidylinositol-specific) | 16q24.1 |
| 166 | PLCH1 | phospholipase C, eta 1 | 3q25.31 |
| 167 | PLCH2 | phospholipase C, eta 2 | 1p36.32 |
| 168 | PLCXD2 | phosphatidylinositol-specific phospholipase C, X domain containing 2 | 3q13.2 |
| 169 | PLCXD3 | phosphatidylinositol-specific phospholipase C, X domain containing 3 | 5p13.1 |
| 170 | PLCZ1 | phospholipase C, zeta 1 | 12p12.3 |
| 171 | PLD1 | phospholipase D1, phosphatidylcholine-specific | 3q26 |
| 172 | PLD2 | phospholipase D2 | 17p13.1 |
| 173 | PLD3 | phospholipase D family, member 3 | 19q13.2 |
| 174 | PLD4 | phospholipase D family, member 4 | 14q32.33 |
| 175 | PLD6 | phospholipase D family, member 6 | 17p11.2 |
| 176 | PLIN1 | perilipin 1 | 15q26 |
| 177 | PNLIP | pancreatic lipase | 10q26.1 |
| 178 | PNLIPRP2 | pancreatic lipase-related protein 2 | 10q25.3 |
| 179 | PNLIPRP3 | pancreatic lipase-related protein 3 | 10q25.3 |
| 180 | PNPLA1 | patatin-like phospholipase domain containing 1 | 6p21.31 |
| 181 | PNPLA2 | patatin-like phospholipase domain containing 2 | 11p15.5 |
| 182 | PNPLA3 | patatin-like phospholipase domain containing 3 | 22q13.31 |
| 183 | PNPLA4 | patatin-like phospholipase domain containing 4 | Xp22.3 |
| 184 | PNPLA5 | patatin-like phospholipase domain containing 5 | 22q13.31 |
| 185 | PNPLA6 | patatin-like phospholipase domain containing 6 | 19p13.2 |
| 186 | PNPLA8 | patatin-like phospholipase domain containing 8 | 7q31 |
| 187 | PPARA | peroxisome proliferator-activated receptor alpha | 22q12-q13.1|22q13.31 |
| 188 | PPARD | peroxisome proliferator-activated receptor delta | 6p21.2 |
| 189 | PPP1CA | protein phosphatase 1, catalytic subunit, alpha isozyme | 11q13 |
| 190 | PPP1CB | protein phosphatase 1, catalytic subunit, beta isozyme | 2p23 |
| 191 | PPP1CC | protein phosphatase 1, catalytic subunit, gamma isozyme | 12q24.1-q24.2 |
| 192 | PPT1 | palmitoyl-protein thioesterase 1 | 1p32 |
| 193 | PRDX6 | peroxiredoxin 6 | 1q25.1 |
| 194 | PRKACA | protein kinase, cAMP-dependent, catalytic, alpha | 19p13.1 |
| 195 | PRKACB | protein kinase, cAMP-dependent, catalytic, beta | 1p36.1 |
| 196 | PRKACG | protein kinase, cAMP-dependent, catalytic, gamma | 9q13 |
| 197 | PROCA1 | protein interacting with cyclin A1 | 17q11.2 |
| 198 | SCARB1 | scavenger receptor class B, member 1 | 12q24.31 |
| 199 | SCARF1 | scavenger receptor class F, member 1 | 17p13.3 |
| 200 | SCP2 | sterol carrier protein 2 | 1p32 |
| 201 | SGPL1 | sphingosine-1-phosphate lyase 1 | 10q21 |
| 202 | SLC25A17 | solute carrier family 25 (mitochondrial carrier; peroxisomal membrane protein, 34kDa), member 17 | 22q13.2 |
| 203 | SLC27A2 | solute carrier family 27 (fatty acid transporter), member 2 | 15q21.2 |
| 204 | SMPD1 | sphingomyelin phosphodiesterase 1, acid lysosomal | 11p15.4-p15.1 |
| 205 | SMPD3 | sphingomyelin phosphodiesterase 3, neutral membrane (neutral sphingomyelinase II) | 16q22.1 |
| 206 | SMPD4 | sphingomyelin phosphodiesterase 4, neutral membrane (neutral sphingomyelinase-3) | 2q21.1 |
| 207 | SMPDL3A | sphingomyelin phosphodiesterase, acid-like 3A | 6q22.31 |
| 208 | SMPDL3B | sphingomyelin phosphodiesterase, acid-like 3B | 1p35.3 |
| 209 | SNX17 | sorting nexin 17 | 2p23-p22 |
| 210 | SPHK1 | sphingosine kinase 1 | 17q25.2 |
| 211 | SRD5A3 | steroid 5 alpha-reductase 3 | 4q12 |
| 212 | STS | steroid sulfatase (microsomal), isozyme S | Xp22.32 |
| 213 | SULT2A1 | sulfotransferase family, cytosolic, 2A, dehydroepiandrosterone (DHEA)-preferring, member 1 | 19q13.3 |
| 214 | THRA | thyroid hormone receptor, alpha (erythroblastic leukemia viral (v-erb-a) oncogene homolog, avian) | 17q11.2 |
| 215 | TNF | tumor necrosis factor | 6p21.3 |
| 216 | TRERF1 | transcriptional regulating factor 1 | 6p21.1-p12.1 |
| 217 | UGT2B4 | UDP glucuronosyltransferase 2 family, polypeptide B4 | 4q13 |
| 218 | YWHAH | tyrosine 3-monooxygenase/tryptophan 5-monooxygenase activation protein, eta polypeptide | 22q12.3 |

Table S4. The list of genes in lipid biosynthetic process (totally 424 genes).

| **ID** | **Symbol** | **Gene full name** | **Location** |
| --- | --- | --- | --- |
| 1 | A4GALT | alpha 1,4-galactosyltransferase | 22q13.2 |
| 2 | A8MWK0 | Fatty acid desaturase 2-like protein | 11q12.1 |
| 3 | ABCB11 | ATP-binding cassette, sub-family B (MDR/TAP), member 11 | 2q24 |
| 4 | ABCC1 | ATP-binding cassette, sub-family C (CFTR/MRP), member 1 | 16p13.1 |
| 5 | ABCG1 | ATP-binding cassette, sub-family G (WHITE), member 1 | 21q22.3 |
| 6 | ABHD5 | abhydrolase domain containing 5 | 3p21 |
| 7 | ACAA2 | acetyl-CoA acyltransferase 2 | 18q21.1 |
| 8 | ACACA | acetyl-CoA carboxylase alpha | 17q21 |
| 9 | ACACB | acetyl-CoA carboxylase beta | 12q24.11 |
| 10 | ACADL | acyl-CoA dehydrogenase, long chain | 2q34-q35 |
| 11 | ACADVL | acyl-CoA dehydrogenase, very long chain | 17p13.1 |
| 12 | ACAT1 | acetyl-CoA acetyltransferase 1 | 11q22.3 |
| 13 | ACBD3 | acyl-CoA binding domain containing 3 | 1q42.12 |
| 14 | ACER2 | alkaline ceramidase 2 | 9p22.1 |
| 15 | ACER3 | alkaline ceramidase 3 | 11q13.5 |
| 16 | ACLY | ATP citrate lyase | 17q21.2 |
| 17 | ACOT8 | acyl-CoA thioesterase 8 | 20q13.12 |
| 18 | ACOX2 | acyl-CoA oxidase 2, branched chain | 3p14.3 |
| 19 | ACSL1 | acyl-CoA synthetase long-chain family member 1 | 4q35 |
| 20 | ACSL3 | acyl-CoA synthetase long-chain family member 3 | 2q34-q35 |
| 21 | ACSL4 | acyl-CoA synthetase long-chain family member 4 | Xq22.3-q23 |
| 22 | ACSL5 | acyl-CoA synthetase long-chain family member 5 | 10q25.1-q25.2 |
| 23 | ACSL6 | acyl-CoA synthetase long-chain family member 6 | 5q31 |
| 24 | ACSM1 | acyl-CoA synthetase medium-chain family member 1 | 16p12.3 |
| 25 | ACSS2 | acyl-CoA synthetase short-chain family member 2 | 20q11.22 |
| 26 | ADM | adrenomedullin | 11p15.4 |
| 27 | ADORA2B | adenosine A2b receptor | 17p12 |
| 28 | AGK | acylglycerol kinase | 7q34 |
| 29 | AGMO | alkylglycerol monooxygenase | 7p21.2 |
| 30 | AGPAT1 | 1-acylglycerol-3-phosphate O-acyltransferase 1 (lysophosphatidic acid acyltransferase, alpha) | 6p21.3 |
| 31 | AGPAT2 | 1-acylglycerol-3-phosphate O-acyltransferase 2 (lysophosphatidic acid acyltransferase, beta) | 9q34.3 |
| 32 | AGPAT3 | 1-acylglycerol-3-phosphate O-acyltransferase 3 | 21q22.3 |
| 33 | AGPAT4 | 1-acylglycerol-3-phosphate O-acyltransferase 4 (lysophosphatidic acid acyltransferase, delta) | 6q26 |
| 34 | AGPAT5 | 1-acylglycerol-3-phosphate O-acyltransferase 5 (lysophosphatidic acid acyltransferase, epsilon) | 8p23.1 |
| 35 | AGPAT6 | 1-acylglycerol-3-phosphate O-acyltransferase 6 (lysophosphatidic acid acyltransferase, zeta) | 8p11.21 |
| 36 | AGPAT9 | 1-acylglycerol-3-phosphate O-acyltransferase 9 | 4q21.23 |
| 37 | AGPS | alkylglycerone phosphate synthase | 2q31.2 |
| 38 | AGT | angiotensinogen (serpin peptidase inhibitor, clade A, member 8) | 1q42-q43 |
| 39 | AKR1B1 | aldo-keto reductase family 1, member B1 (aldose reductase) | 7q35 |
| 40 | AKR1C4 | aldo-keto reductase family 1, member C4 (chlordecone reductase; 3-alpha hydroxysteroid dehydrogenase, type I; dihydrodiol dehydrogenase 4) | 10p15.1 |
| 41 | AKR1D1 | aldo-keto reductase family 1, member D1 (delta 4-3-ketosteroid-5-beta-reductase) | 7q32-q33 |
| 42 | AKT1 | v-akt murine thymoma viral oncogene homolog 1 | 14q32.32|14q32.32 |
| 43 | ALDH8A1 | aldehyde dehydrogenase 8 family, member A1 | 6q23.2 |
| 44 | ALG1 | asparagine-linked glycosylation 1, beta-1,4-mannosyltransferase homolog (S. cerevisiae) | 16p13.3 |
| 45 | ALG12 | asparagine-linked glycosylation 12, alpha-1,6-mannosyltransferase homolog (S. cerevisiae) | 22q13.33 |
| 46 | ALG9 | asparagine-linked glycosylation 9, alpha-1,2-mannosyltransferase homolog (S. cerevisiae) | 11q23 |
| 47 | ALOX12 | arachidonate 12-lipoxygenase | 17p13.1 |
| 48 | ALOX12B | arachidonate 12-lipoxygenase, 12R type | 17p13.1 |
| 49 | ALOX15 | arachidonate 15-lipoxygenase | 17p13.3 |
| 50 | ALOX15B | arachidonate 15-lipoxygenase, type B | 17p13.1 |
| 51 | ALOX5 | arachidonate 5-lipoxygenase | 10q11.2 |
| 52 | ALOX5AP | arachidonate 5-lipoxygenase-activating protein | 13q12 |
| 53 | ALOXE3 | arachidonate lipoxygenase 3 | 17p13.1 |
| 54 | AMACR | alpha-methylacyl-CoA racemase | 5p13 |
| 55 | ANG | angiogenin, ribonuclease, RNase A family, 5 | 14q11.1-q11.2 |
| 56 | AOPEP | chromosome 9 open reading frame 3 | 9q22.32 |
| 57 | APOA1 | apolipoprotein A-I | 11q23-q24 |
| 58 | APOA2 | apolipoprotein A-II | 1q21-q23 |
| 59 | APOA4 | apolipoprotein A-IV | 11q23 |
| 60 | APOA5 | apolipoprotein A-V | 11q23 |
| 61 | APOB | apolipoprotein B (including Ag(x) antigen) | 2p24-p23 |
| 62 | APOC1 | apolipoprotein C-I | 19q13.2 |
| 63 | APOC2 | apolipoprotein C-II | 19q13.2 |
| 64 | APOC3 | apolipoprotein C-III | 11q23.1-q23.2 |
| 65 | APOE | apolipoprotein E | 19q13.2 |
| 66 | AVP | arginine vasopressin | 20p13 |
| 67 | AVPR1A | arginine vasopressin receptor 1A | 12q14-q15 |
| 68 | AWAT1 | acyl-CoA wax alcohol acyltransferase 1 | Xq13.1 |
| 69 | AWAT2 | acyl-CoA wax alcohol acyltransferase 2 | Xq13.1 |
| 70 | B3GNT5 | UDP-GlcNAc:betaGal beta-1,3-N-acetylglucosaminyltransferase 5 | 3q28 |
| 71 | B4GALNT1 | beta-1,4-N-acetyl-galactosaminyl transferase 1 | 12q13.3 |
| 72 | BAAT | bile acid CoA: amino acid N-acyltransferase (glycine N-choloyltransferase) | 9q22.3 |
| 73 | BMP6 | bone morphogenetic protein 6 | 6p24-p23 |
| 74 | BRCA1 | breast cancer 1, early onset | 17q21 |
| 75 | C14orf1 | chromosome 14 open reading frame 1 | 14q24.3 |
| 76 | C1orf93 | chromosome 1 open reading frame 93 | 1p36.32 |
| 77 | C5orf4 | chromosome 5 open reading frame 4 | 5q31-q32 |
| 78 | CBR4 | carbonyl reductase 4 | 4q32.3 |
| 79 | CD74 | CD74 molecule, major histocompatibility complex, class II invariant chain | 5q32 |
| 80 | CD81 | CD81 molecule | 11p15.5 |
| 81 | CDIPT | CDP-diacylglycerol--inositol 3-phosphatidyltransferase | 16p11.2 |
| 82 | CDS1 | CDP-diacylglycerol synthase (phosphatidate cytidylyltransferase) 1 | 4q21.23 |
| 83 | CDS2 | CDP-diacylglycerol synthase (phosphatidate cytidylyltransferase) 2 | 20p13 |
| 84 | CEPT1 | choline/ethanolamine phosphotransferase 1 | 1p13.3 |
| 85 | CERCAM | cerebral endothelial cell adhesion molecule | 9q34.11 |
| 86 | CFTR | cystic fibrosis transmembrane conductance regulator (ATP-binding cassette sub-family C, member 7) | 7q31.2 |
| 87 | CH25H | cholesterol 25-hydroxylase | 10q23 |
| 88 | CHKA | choline kinase alpha | 11q13.2 |
| 89 | CHKB | choline kinase beta | 22q13.33 |
| 90 | CHPT1 | choline phosphotransferase 1 | 12q |
| 91 | CLN8 | ceroid-lipofuscinosis, neuronal 8 (epilepsy, progressive with mental retardation) | 8p23 |
| 92 | CMAS | cytidine monophosphate N-acetylneuraminic acid synthetase | 12p12.1 |
| 93 | CNBP | CCHC-type zinc finger, nucleic acid binding protein | 3q21 |
| 94 | COQ2 | coenzyme Q2 homolog, prenyltransferase (yeast) | 4q21.23 |
| 95 | CRH | corticotropin releasing hormone | 8q13 |
| 96 | CRLS1 | cardiolipin synthase 1 | 20p13-p12.3 |
| 97 | CWH43 | cell wall biogenesis 43 C-terminal homolog (S. cerevisiae) | 4p11 |
| 98 | CYB5R1 | cytochrome b5 reductase 1 | 1p36.13-q41 |
| 99 | CYB5R2 | cytochrome b5 reductase 2 | 11p15.4 |
| 100 | CYB5R3 | cytochrome b5 reductase 3 | 22q13.31-qter|22q13.2-q13.31 |
| 101 | CYP11A1 | cytochrome P450, family 11, subfamily A, polypeptide 1 | 15q23-q24 |
| 102 | CYP11B1 | cytochrome P450, family 11, subfamily B, polypeptide 1 | 8q21 |
| 103 | CYP11B2 | cytochrome P450, family 11, subfamily B, polypeptide 2 | 8q21-q22 |
| 104 | CYP17A1 | cytochrome P450, family 17, subfamily A, polypeptide 1 | 10q24.3 |
| 105 | CYP19A1 | cytochrome P450, family 19, subfamily A, polypeptide 1 | 15q21.1 |
| 106 | CYP21A2 | cytochrome P450, family 21, subfamily A, polypeptide 2 | 6p21.3 |
| 107 | CYP27A1 | cytochrome P450, family 27, subfamily A, polypeptide 1 | 2q33-qter |
| 108 | CYP27B1 | cytochrome P450, family 27, subfamily B, polypeptide 1 | 12q13.1-q13.3 |
| 109 | CYP39A1 | cytochrome P450, family 39, subfamily A, polypeptide 1 | 6p21.1-p11.2 |
| 110 | CYP46A1 | cytochrome P450, family 46, subfamily A, polypeptide 1 | 14q32.1 |
| 111 | CYP51A1 | cytochrome P450, family 51, subfamily A, polypeptide 1 | 7q21.2 |
| 112 | CYP7A1 | cytochrome P450, family 7, subfamily A, polypeptide 1 | 8q11-q12 |
| 113 | CYP7B1 | cytochrome P450, family 7, subfamily B, polypeptide 1 | 8q21.3 |
| 114 | CYP8B1 | cytochrome P450, family 8, subfamily B, polypeptide 1 | 3p22.1 |
| 115 | DEGS1 | degenerative spermatocyte homolog 1, lipid desaturase (Drosophila) | 1q42.11 |
| 116 | DEGS2 | degenerative spermatocyte homolog 2, lipid desaturase (Drosophila) | 14q32.2 |
| 117 | DGAT1 | diacylglycerol O-acyltransferase 1 | 8q24.3 |
| 118 | DGAT2 | diacylglycerol O-acyltransferase 2 | 11q13.5 |
| 119 | DGAT2L6 | diacylglycerol O-acyltransferase 2-like 6 | Xq13.1 |
| 120 | DGAT2L7 | - | 7q22.1 |
| 121 | DGKE | diacylglycerol kinase, epsilon 64kDa | 17q22 |
| 122 | DHCR24 | 24-dehydrocholesterol reductase | 1p32.3 |
| 123 | DHCR7 | 7-dehydrocholesterol reductase | 11q13.4 |
| 124 | DHH | desert hedgehog | 12q13.1 |
| 125 | DHRS9 | dehydrogenase/reductase (SDR family) member 9 | 2q31.1 |
| 126 | DKK3 | dickkopf homolog 3 (Xenopus laevis) | 11p15.2 |
| 127 | DOLK | dolichol kinase | 9q34.11 |
| 128 | DOLPP1 | dolichyl pyrophosphate phosphatase 1 | 9q34.1 |
| 129 | DPAGT1 | dolichyl-phosphate (UDP-N-acetylglucosamine) N-acetylglucosaminephosphotransferase 1 (GlcNAc-1-P transferase) | 11q23.3 |
| 130 | DPEP2 | dipeptidase 2 | 16q22.1 |
| 131 | DPM1 | dolichyl-phosphate mannosyltransferase polypeptide 1, catalytic subunit | 20q13.13 |
| 132 | DPM2 | dolichyl-phosphate mannosyltransferase polypeptide 2, regulatory subunit | 9q34.13 |
| 133 | DPM3 | dolichyl-phosphate mannosyltransferase polypeptide 3 | 1q22 |
| 134 | EBP | emopamil binding protein (sterol isomerase) | Xp11.23-p11.22 |
| 135 | EDN1 | endothelin 1 | 6p24.1 |
| 136 | EDN2 | endothelin 2 | 1p34 |
| 137 | ELOVL1 | elongation of very long chain fatty acids (FEN1/Elo2, SUR4/Elo3, yeast)-like 1 | 1p34.2 |
| 138 | ELOVL2 | elongation of very long chain fatty acids (FEN1/Elo2, SUR4/Elo3, yeast)-like 2 | 6p24.2 |
| 139 | ELOVL3 | elongation of very long chain fatty acids (FEN1/Elo2, SUR4/Elo3, yeast)-like 3 | 10q24.32 |
| 140 | ELOVL4 | elongation of very long chain fatty acids (FEN1/Elo2, SUR4/Elo3, yeast)-like 4 | 6q14 |
| 141 | ELOVL5 | ELOVL family member 5, elongation of long chain fatty acids (FEN1/Elo2, SUR4/Elo3-like, yeast) | 6p21.1-p12.1 |
| 142 | ELOVL6 | ELOVL family member 6, elongation of long chain fatty acids (FEN1/Elo2, SUR4/Elo3-like, yeast) | 4q25 |
| 143 | ELOVL7 | ELOVL family member 7, elongation of long chain fatty acids (yeast) | 5q12.1 |
| 144 | EPT1 | ethanolaminephosphotransferase 1 (CDP-ethanolamine-specific) | 2p23.3 |
| 145 | ETNK1 | ethanolamine kinase 1 | 12p12.1 |
| 146 | ETNK2 | ethanolamine kinase 2 | 1q32.1 |
| 147 | FA2H | fatty acid 2-hydroxylase | 16q23 |
| 148 | FABP3 | fatty acid binding protein 3, muscle and heart (mammary-derived growth inhibitor) | 1p33-p32 |
| 149 | FABP5 | fatty acid binding protein 5 (psoriasis-associated) | 8q21.13 |
| 150 | FABP5P3 | fatty acid binding protein 5 pseudogene 3 | 7q36.1 |
| 151 | FADS1 | fatty acid desaturase 1 | 11q12.2-q13.1 |
| 152 | FADS2 | fatty acid desaturase 2 | 11q12.2 |
| 153 | FADS3 | fatty acid desaturase 3 | 11q12-q13.1 |
| 154 | FADS6 | fatty acid desaturase domain family, member 6 | 17q25.1 |
| 155 | FAR1 | fatty acyl CoA reductase 1 | 11p15.2 |
| 156 | FAR2 | fatty acyl CoA reductase 2 | 12p11.22 |
| 157 | FASN | fatty acid synthase | 17q25 |
| 158 | FCER1A | Fc fragment of IgE, high affinity I, receptor for; alpha polypeptide | 1q23 |
| 159 | FDFT1 | farnesyl-diphosphate farnesyltransferase 1 | 8p23.1-p22 |
| 160 | FDPS | farnesyl diphosphate synthase | 1q22 |
| 161 | FDXR | ferredoxin reductase | 17q24-q25 |
| 162 | FGF1 | fibroblast growth factor 1 (acidic) | 5q31 |
| 163 | FGF19 | fibroblast growth factor 19 | 11q13.1 |
| 164 | FGF2 | fibroblast growth factor 2 (basic) | 4q26 |
| 165 | FGF7 | fibroblast growth factor 7 | 15q21.2 |
| 166 | FITM2 | fat storage-inducing transmembrane protein 2 | 20q13.12 |
| 167 | FSHB | follicle stimulating hormone, beta polypeptide | 11p13 |
| 168 | G6PD | glucose-6-phosphate dehydrogenase | Xq28 |
| 169 | GAL3ST1 | galactose-3-O-sulfotransferase 1 | 22q12.2 |
| 170 | GBGT1 | globoside alpha-1,3-N-acetylgalactosaminyltransferase 1 | 9q34.13-q34.3 |
| 171 | GFI1 | growth factor independent 1 transcription repressor | 1p22 |
| 172 | GGPS1 | geranylgeranyl diphosphate synthase 1 | 1q43 |
| 173 | GGT5 | gamma-glutamyltransferase 5 | 22q11.23 |
| 174 | GK | glycerol kinase | Xp21.3 |
| 175 | GLT25D1 | glycosyltransferase 25 domain containing 1 | 19p13.11 |
| 176 | GLT25D2 | glycosyltransferase 25 domain containing 2 | 1q25 |
| 177 | GNE | glucosamine (UDP-N-acetyl)-2-epimerase/N-acetylmannosamine kinase | 9p13.3 |
| 178 | GNPAT | glyceronephosphate O-acyltransferase | 1q42 |
| 179 | GPAA1 | glycosylphosphatidylinositol anchor attachment protein 1 homolog (yeast) | 8q24.3 |
| 180 | GPAM | glycerol-3-phosphate acyltransferase, mitochondrial | 10q25.2 |
| 181 | GPAT2 | glycerol-3-phosphate acyltransferase 2, mitochondrial | 2q11.1 |
| 182 | GPD1 | glycerol-3-phosphate dehydrogenase 1 (soluble) | 12q12-q13 |
| 183 | HEXB | hexosaminidase B (beta polypeptide) | 5q13 |
| 184 | HINT2 | histidine triad nucleotide binding protein 2 | 9p13.3 |
| 185 | HMGCL | 3-hydroxymethyl-3-methylglutaryl-CoA lyase | 1p36.1-p35 |
| 186 | HMGCR | 3-hydroxy-3-methylglutaryl-CoA reductase | 5q13.3-q14 |
| 187 | HMGCS1 | 3-hydroxy-3-methylglutaryl-CoA synthase 1 (soluble) | 5p14-p13 |
| 188 | HMGCS2 | 3-hydroxy-3-methylglutaryl-CoA synthase 2 (mitochondrial) | 1p13-p12 |
| 189 | HNF1A | HNF1 homeobox A | 12q22-qter|12q24.2 |
| 190 | HPGDS | hematopoietic prostaglandin D synthase | 4q22.3 |
| 191 | HRH1 | histamine receptor H1 | 3p25 |
| 192 | HSD11B1 | hydroxysteroid (11-beta) dehydrogenase 1 | 1q32-q41 |
| 193 | HSD11B2 | hydroxysteroid (11-beta) dehydrogenase 2 | 16q22 |
| 194 | HSD17B1 | hydroxysteroid (17-beta) dehydrogenase 1 | 17q11-q21 |
| 195 | HSD17B11 | hydroxysteroid (17-beta) dehydrogenase 11 | 4q22.1 |
| 196 | HSD17B12 | hydroxysteroid (17-beta) dehydrogenase 12 | 11p11.2 |
| 197 | HSD17B2 | hydroxysteroid (17-beta) dehydrogenase 2 | 16q24.1-q24.2 |
| 198 | HSD17B3 | hydroxysteroid (17-beta) dehydrogenase 3 | 9q22 |
| 199 | HSD17B4 | hydroxysteroid (17-beta) dehydrogenase 4 | 5q21 |
| 200 | HSD17B6 | hydroxysteroid (17-beta) dehydrogenase 6 homolog (mouse) | 12q13 |
| 201 | HSD17B7 | hydroxysteroid (17-beta) dehydrogenase 7 | 1q23 |
| 202 | HSD17B8 | hydroxysteroid (17-beta) dehydrogenase 8 | 6p21.3 |
| 203 | HSD3B1 | hydroxy-delta-5-steroid dehydrogenase, 3 beta- and steroid delta-isomerase 1 | 1p13.1 |
| 204 | HSD3B2 | hydroxy-delta-5-steroid dehydrogenase, 3 beta- and steroid delta-isomerase 2 | 1p13.1 |
| 205 | HSD3B7 | hydroxy-delta-5-steroid dehydrogenase, 3 beta- and steroid delta-isomerase 7 | 16p11.2 |
| 206 | HTR2A | 5-hydroxytryptamine (serotonin) receptor 2A | 13q14-q21 |
| 207 | HTR2B | 5-hydroxytryptamine (serotonin) receptor 2B | 2q36.3-q37.1 |
| 208 | HTR2C | 5-hydroxytryptamine (serotonin) receptor 2C | Xq24 |
| 209 | IDI1 | isopentenyl-diphosphate delta isomerase 1 | 10p15.3 |
| 210 | IDI2 | isopentenyl-diphosphate delta isomerase 2 | 10p15.3 |
| 211 | IFNG | interferon, gamma | 12q14 |
| 212 | IL1A | interleukin 1, alpha | 2q14 |
| 213 | IMPA1 | inositol(myo)-1(or 4)-monophosphatase 1 | 8q21.13-q21.3 |
| 214 | INS | insulin | 11p15.5 |
| 215 | INSIG1 | insulin induced gene 1 | 7q36 |
| 216 | INSIG2 | insulin induced gene 2 | 2q14.2 |
| 217 | ISPD | isoprenoid synthase domain containing | 7p21.2 |
| 218 | ISYNA1 | inositol-3-phosphate synthase 1 | 19p13.11 |
| 219 | KGFLP1 | - | 9p11.2 |
| 220 | KGFLP2 | - | 9p12 |
| 221 | LARGE | like-glycosyltransferase | 22q12.3 |
| 222 | LASS1 | LAG1 homolog, ceramide synthase 1 | 19p12 |
| 223 | LASS2 | LAG1 homolog, ceramide synthase 2 | 1q21.3 |
| 224 | LASS3 | LAG1 homolog, ceramide synthase 3 | 15q26.3 |
| 225 | LASS4 | LAG1 homolog, ceramide synthase 4 | 19p13.2 |
| 226 | LASS5 | LAG1 homolog, ceramide synthase 5 | 12q13.12 |
| 227 | LASS6 | LAG1 homolog, ceramide synthase 6 | 2q24.3 |
| 228 | LBR | lamin B receptor | 1q42.1 |
| 229 | LCAT | lecithin-cholesterol acyltransferase | 16q22.1 |
| 230 | LCLAT1 | lysocardiolipin acyltransferase 1 | 2p23.1 |
| 231 | LEP | leptin | 7q31.3 |
| 232 | LHB | luteinizing hormone beta polypeptide | 19q13.32 |
| 233 | LHCGR | luteinizing hormone/choriogonadotropin receptor | 2p21 |
| 234 | LIAS | lipoic acid synthetase | 4p14 |
| 235 | LIPC | lipase, hepatic | 15q21-q23 |
| 236 | LIPT2 | lipoyl(octanoyl) transferase 2 (putative) | 11q13.4 |
| 237 | LPCAT1 | lysophosphatidylcholine acyltransferase 1 | 5p15.33 |
| 238 | LPCAT2 | lysophosphatidylcholine acyltransferase 2 | 16q12.2 |
| 239 | LPCAT3 | lysophosphatidylcholine acyltransferase 3 | 12p13 |
| 240 | LPCAT4 | lysophosphatidylcholine acyltransferase 4 | 15q14 |
| 241 | LPGAT1 | lysophosphatidylglycerol acyltransferase 1 | 1q32 |
| 242 | LPIN1 | lipin 1 | 2p25.1 |
| 243 | LPIN2 | lipin 2 | 18p11.31 |
| 244 | LPL | lipoprotein lipase | 8p22 |
| 245 | LSS | lanosterol synthase (2,3-oxidosqualene-lanosterol cyclase) | 21q22.3 |
| 246 | LTA4H | leukotriene A4 hydrolase | 12q22 |
| 247 | LTC4S | leukotriene C4 synthase | 5q35 |
| 248 | MAPKAPK2 | mitogen-activated protein kinase-activated protein kinase 2 | 1q32 |
| 249 | MBOAT1 | membrane bound O-acyltransferase domain containing 1 | 6p22.3 |
| 250 | MBOAT2 | membrane bound O-acyltransferase domain containing 2 | 2p25.1 |
| 251 | MBOAT7 | membrane bound O-acyltransferase domain containing 7 | 19q13.4 |
| 252 | MCAT | malonyl CoA:ACP acyltransferase (mitochondrial) | 22q13.31 |
| 253 | MECR | mitochondrial trans-2-enoyl-CoA reductase | 1p36.1-p35.1 |
| 254 | MED1 | mediator complex subunit 1 | 17q12 |
| 255 | MGLL | monoglyceride lipase | 3q21.3 |
| 256 | MGST2 | microsomal glutathione S-transferase 2 | 4q28.3 |
| 257 | MID1IP1 | MID1 interacting protein 1 (gastrulation specific G12 homolog (zebrafish)) | Xp11.4 |
| 258 | MIF | macrophage migration inhibitory factor (glycosylation-inhibiting factor) | 22q11.23 |
| 259 | MLXIPL | MLX interacting protein-like | 7q11.23 |
| 260 | MLYCD | malonyl-CoA decarboxylase | 16q24 |
| 261 | MOGAT1 | monoacylglycerol O-acyltransferase 1 | 2q36.1 |
| 262 | MOGAT2 | monoacylglycerol O-acyltransferase 2 | 11q13.5 |
| 263 | MOGAT3 | monoacylglycerol O-acyltransferase 3 | 7q22.1 |
| 264 | MPPE1 | metallophosphoesterase 1 | 18p11.21 |
| 265 | MVD | mevalonate (diphospho) decarboxylase | 16q24.3 |
| 266 | MVK | mevalonate kinase | 12q24 |
| 267 | NANS | N-acetylneuraminic acid synthase | 9p24.1-p23 |
| 268 | NDUFAB1 | NADH dehydrogenase (ubiquinone) 1, alpha/beta subcomplex, 1, 8kDa | 16p12.2 |
| 269 | NFKB1 | nuclear factor of kappa light polypeptide gene enhancer in B-cells 1 | 4q24 |
| 270 | NPC1L1 | NPC1 (Niemann-Pick disease, type C1, gene)-like 1 | 7p13 |
| 271 | NR0B1 | nuclear receptor subfamily 0, group B, member 1 | Xp21.3 |
| 272 | NR1H2 | nuclear receptor subfamily 1, group H, member 2 | 19q13.3 |
| 273 | NR1H3 | nuclear receptor subfamily 1, group H, member 3 | 11p11.2 |
| 274 | NR3C1 | nuclear receptor subfamily 3, group C, member 1 (glucocorticoid receptor) | 5q31.3 |
| 275 | NR5A1 | nuclear receptor subfamily 5, group A, member 1 | 9q33 |
| 276 | NSDHL | NAD(P) dependent steroid dehydrogenase-like | Xq28 |
| 277 | OLAH | oleoyl-ACP hydrolase | 10p13 |
| 278 | OXSM | 3-oxoacyl-ACP synthase, mitochondrial | 3p24.2 |
| 279 | P2RX1 | purinergic receptor P2X, ligand-gated ion channel, 1 | 17p13.3 |
| 280 | P2RX7 | purinergic receptor P2X, ligand-gated ion channel, 7 | 12q24 |
| 281 | PBX1 | pre-B-cell leukemia homeobox 1 | 1q23 |
| 282 | PC | pyruvate carboxylase | 11q13.4-q13.5 |
| 283 | PCK1 | phosphoenolpyruvate carboxykinase 1 (soluble) | 20q13.31 |
| 284 | PCYT1A | phosphate cytidylyltransferase 1, choline, alpha | 3q29 |
| 285 | PCYT1B | phosphate cytidylyltransferase 1, choline, beta | Xp22.11 |
| 286 | PCYT2 | phosphate cytidylyltransferase 2, ethanolamine | 17q25.3 |
| 287 | PDGFA | platelet-derived growth factor alpha polypeptide | 7p22 |
| 288 | PDGFB | platelet-derived growth factor beta polypeptide (simian sarcoma viral (v-sis) oncogene homolog) | 22q12.3-q13.1|22q13.1 |
| 289 | PDSS1 | prenyl (decaprenyl) diphosphate synthase, subunit 1 | 10p12.1 |
| 290 | PDSS2 | prenyl (decaprenyl) diphosphate synthase, subunit 2 | 6q21 |
| 291 | PECR | peroxisomal trans-2-enoyl-CoA reductase | 2q35 |
| 292 | PEMT | phosphatidylethanolamine N-methyltransferase | 17p11.2 |
| 293 | PEX2 | peroxisomal biogenesis factor 2 | 8q21.1 |
| 294 | PEX7 | peroxisomal biogenesis factor 7 | 6q23.3 |
| 295 | PGAP1 | post-GPI attachment to proteins 1 | 2q33.1 |
| 296 | PGAP2 | post-GPI attachment to proteins 2 | 11p15.5 |
| 297 | PGAP3 | post-GPI attachment to proteins 3 | 17q12 |
| 298 | PGS1 | phosphatidylglycerophosphate synthase 1 | 17q25.3 |
| 299 | PI4K2A | phosphatidylinositol 4-kinase type 2 alpha | 10q24 |
| 300 | PI4KA | phosphatidylinositol 4-kinase, catalytic, alpha | 22q11.21 |
| 301 | PI4KB | phosphatidylinositol 4-kinase, catalytic, beta | 1q21 |
| 302 | PIGA | phosphatidylinositol glycan anchor biosynthesis, class A | Xp22.1 |
| 303 | PIGB | phosphatidylinositol glycan anchor biosynthesis, class B | 15q21-q22 |
| 304 | PIGC | phosphatidylinositol glycan anchor biosynthesis, class C | 1q23-q25 |
| 305 | PIGF | phosphatidylinositol glycan anchor biosynthesis, class F | 2p21-p16 |
| 306 | PIGG | phosphatidylinositol glycan anchor biosynthesis, class G | 4p16.3 |
| 307 | PIGH | phosphatidylinositol glycan anchor biosynthesis, class H | 14q24.1 |
| 308 | PIGK | phosphatidylinositol glycan anchor biosynthesis, class K | 1p31.1 |
| 309 | PIGL | phosphatidylinositol glycan anchor biosynthesis, class L | 17p12-p11.2 |
| 310 | PIGM | phosphatidylinositol glycan anchor biosynthesis, class M | 1q23.2 |
| 311 | PIGN | phosphatidylinositol glycan anchor biosynthesis, class N | 18q21.33 |
| 312 | PIGO | phosphatidylinositol glycan anchor biosynthesis, class O | 9p13.3 |
| 313 | PIGP | phosphatidylinositol glycan anchor biosynthesis, class P | 21q22.2 |
| 314 | PIGQ | phosphatidylinositol glycan anchor biosynthesis, class Q | 16p13.3 |
| 315 | PIGS | phosphatidylinositol glycan anchor biosynthesis, class S | 17p13.2 |
| 316 | PIGT | phosphatidylinositol glycan anchor biosynthesis, class T | 20q12-q13.12 |
| 317 | PIGU | phosphatidylinositol glycan anchor biosynthesis, class U | 20q11.22 |
| 318 | PIGV | phosphatidylinositol glycan anchor biosynthesis, class V | 1p36.11 |
| 319 | PIGW | phosphatidylinositol glycan anchor biosynthesis, class W | 17q12 |
| 320 | PIGX | phosphatidylinositol glycan anchor biosynthesis, class X | 3q29 |
| 321 | PIGY | phosphatidylinositol glycan anchor biosynthesis, class Y | 4q22.1 |
| 322 | PIGZ | phosphatidylinositol glycan anchor biosynthesis, class Z | 3q29 |
| 323 | PIK3C2A | phosphoinositide-3-kinase, class 2, alpha polypeptide | 11p15.5-p14 |
| 324 | PIP5K1A | phosphatidylinositol-4-phosphate 5-kinase, type I, alpha | 1q21.3 |
| 325 | PISD | phosphatidylserine decarboxylase | 22q12.2 |
| 326 | PLA2G1B | phospholipase A2, group IB (pancreas) | 12q23-q24.1 |
| 327 | PLA2G4A | phospholipase A2, group IVA (cytosolic, calcium-dependent) | 1q25 |
| 328 | PLA2G5 | phospholipase A2, group V | 1p36-p34 |
| 329 | PLA2G6 | phospholipase A2, group VI (cytosolic, calcium-independent) | 22q13.1 |
| 330 | PLAUR | plasminogen activator, urokinase receptor | 19q13 |
| 331 | PLCE1 | phospholipase C, epsilon 1 | 10q23 |
| 332 | PLD1 | phospholipase D1, phosphatidylcholine-specific | 3q26 |
| 333 | PLP1 | proteolipid protein 1 | Xq22 |
| 334 | PMVK | phosphomevalonate kinase | 1q22 |
| 335 | PNPLA3 | patatin-like phospholipase domain containing 3 | 22q13.31 |
| 336 | PPARD | peroxisome proliferator-activated receptor delta | 6p21.2 |
| 337 | PREY | phosphatidylinositol glycan anchor biosynthesis, class Y | 4q22.1 |
| 338 | PRG3 | proteoglycan 3 | 11q12 |
| 339 | PRKAA1 | protein kinase, AMP-activated, alpha 1 catalytic subunit | 5p12 |
| 340 | PRKAA2 | protein kinase, AMP-activated, alpha 2 catalytic subunit | 1p31 |
| 341 | PRKAB1 | protein kinase, AMP-activated, beta 1 non-catalytic subunit | 12q24.1 |
| 342 | PRKAB2 | protein kinase, AMP-activated, beta 2 non-catalytic subunit | 1q21.1 |
| 343 | PRKAG1 | protein kinase, AMP-activated, gamma 1 non-catalytic subunit | 12q12-q14 |
| 344 | PRKAG2 | protein kinase, AMP-activated, gamma 2 non-catalytic subunit | 7q36.1 |
| 345 | PRKAG3 | protein kinase, AMP-activated, gamma 3 non-catalytic subunit | 2q35 |
| 346 | PRLR | prolactin receptor | 5p13.2 |
| 347 | PROX1 | prospero homeobox 1 | 1q41 |
| 348 | PRPF19 | PRP19/PSO4 pre-mRNA processing factor 19 homolog (S. cerevisiae) | 11q12.2 |
| 349 | PTDSS1 | phosphatidylserine synthase 1 | 8q22 |
| 350 | PTDSS2 | phosphatidylserine synthase 2 | 11p15.5 |
| 351 | PTGDS | prostaglandin D2 synthase 21kDa (brain) | 9q34.2-q34.3 |
| 352 | PTGES | prostaglandin E synthase | 9q34.3 |
| 353 | PTGES2 | prostaglandin E synthase 2 | 9q34.11 |
| 354 | PTGES3 | prostaglandin E synthase 3 (cytosolic) | 12q13.3|12 |
| 355 | PTGIS | prostaglandin I2 (prostacyclin) synthase | 20q13.13 |
| 356 | PTGS1 | prostaglandin-endoperoxide synthase 1 (prostaglandin G/H synthase and cyclooxygenase) | 9q32-q33.3 |
| 357 | PTGS2 | prostaglandin-endoperoxide synthase 2 (prostaglandin G/H synthase and cyclooxygenase) | 1q25.2-q25.3 |
| 358 | PTPLA | protein tyrosine phosphatase-like (proline instead of catalytic arginine), member A | 10p14-p13 |
| 359 | PTPLAD1 | protein tyrosine phosphatase-like A domain containing 1 | 15q22.2 |
| 360 | PTPLAD2 | protein tyrosine phosphatase-like A domain containing 2 | 9p21.3 |
| 361 | PTPLB | protein tyrosine phosphatase-like (proline instead of catalytic arginine), member b | 3q21.1 |
| 362 | RDH8 | retinol dehydrogenase 8 (all-trans) | 19p13.2 |
| 363 | RNPEP | arginyl aminopeptidase (aminopeptidase B) | 1q32 |
| 364 | RNPEPL1 | arginyl aminopeptidase (aminopeptidase B)-like 1 | 2q37.3 |
| 365 | SAMD8 | sterile alpha motif domain containing 8 | 10q22.2 |
| 366 | SC4MOL | sterol-C4-methyl oxidase-like | 4q32-q34 |
| 367 | SC5DL | sterol-C5-desaturase (ERG3 delta-5-desaturase homolog, S. cerevisiae)-like | 11q23.3 |
| 368 | SCAP | SREBF chaperone | 3p21.31 |
| 369 | SCARB1 | scavenger receptor class B, member 1 | 12q24.31 |
| 370 | SCD | stearoyl-CoA desaturase (delta-9-desaturase) | 10q24.31 |
| 371 | SCD5 | stearoyl-CoA desaturase 5 | 4q21.22 |
| 372 | SCP2 | sterol carrier protein 2 | 1p32 |
| 373 | SDR42E1 | short chain dehydrogenase/reductase family 42E, member 1 | 16q23.3 |
| 374 | SDR42E2 | short chain dehydrogenase/reductase family 42E, member 2 | 16p12.1 |
| 375 | SEC14L2 | SEC14-like 2 (S. cerevisiae) | 22q12.2 |
| 376 | SERINC1 | serine incorporator 1 | 6q22.31 |
| 377 | SERINC4 | serine incorporator 4 | 15q15.3 |
| 378 | SERINC5 | serine incorporator 5 | 5q14.1 |
| 379 | SGMS1 | sphingomyelin synthase 1 | 10q11.2 |
| 380 | SGMS2 | sphingomyelin synthase 2 | 4q25 |
| 381 | SIGMAR1 | sigma non-opioid intracellular receptor 1 | 9p13.3 |
| 382 | SLC25A1 | solute carrier family 25 (mitochondrial carrier; citrate transporter), member 1 | 22q11.21 |
| 383 | SLC27A1 | solute carrier family 27 (fatty acid transporter), member 1 | 19p13.11 |
| 384 | SLC27A2 | solute carrier family 27 (fatty acid transporter), member 2 | 15q21.2 |
| 385 | SLC27A5 | solute carrier family 27 (fatty acid transporter), member 5 | 19q13.43 |
| 386 | SMPD2 | sphingomyelin phosphodiesterase 2, neutral membrane (neutral sphingomyelinase) | 6q21 |
| 387 | SOD1 | superoxide dismutase 1, soluble | 21q22.1|21q22.11 |
| 388 | SORBS1 | sorbin and SH3 domain containing 1 | 10q23.33 |
| 389 | SPHK2 | sphingosine kinase 2 | 19q13.2 |
| 390 | SPTLC1 | serine palmitoyltransferase, long chain base subunit 1 | 9q22.2 |
| 391 | SPTLC2 | serine palmitoyltransferase, long chain base subunit 2 | 14q24.3 |
| 392 | SPTLC3 | serine palmitoyltransferase, long chain base subunit 3 | 20p12.1 |
| 393 | SQLE | squalene epoxidase | 8q24.1 |
| 394 | SRD5A1 | steroid-5-alpha-reductase, alpha polypeptide 1 (3-oxo-5 alpha-steroid delta 4-dehydrogenase alpha 1) | 5p15 |
| 395 | SRD5A2 | steroid-5-alpha-reductase, alpha polypeptide 2 (3-oxo-5 alpha-steroid delta 4-dehydrogenase alpha 2) | 2p23 |
| 396 | SRD5A3 | steroid 5 alpha-reductase 3 | 4q12 |
| 397 | SSSPTA | serine palmitoyltransferase, small subunit A | 14q13.1 |
| 398 | SSSPTB | serine palmitoyltransferase, small subunit B | 3q26.1 |
| 399 | ST3GAL5 | ST3 beta-galactoside alpha-2,3-sialyltransferase 5 | 2p11.2 |
| 400 | ST6GALNAC6 | ST6 (alpha-N-acetyl-neuraminyl-2,3-beta-galactosyl-1,3)-N-acetylgalactosaminide alpha-2,6-sialyltransferase 6 | 9q34.11 |
| 401 | ST8SIA1 | ST8 alpha-N-acetyl-neuraminide alpha-2,8-sialyltransferase 1 | 12p12.1-p11.2 |
| 402 | ST8SIA3 | ST8 alpha-N-acetyl-neuraminide alpha-2,8-sialyltransferase 3 | 18q21.31 |
| 403 | ST8SIA5 | ST8 alpha-N-acetyl-neuraminide alpha-2,8-sialyltransferase 5 | 18q21.1 |
| 404 | ST8SIA6 | ST8 alpha-N-acetyl-neuraminide alpha-2,8-sialyltransferase 6 | 10p12.33 |
| 405 | STAR | steroidogenic acute regulatory protein | 8p11.2 |
| 406 | STARD3 | StAR-related lipid transfer (START) domain containing 3 | 17q11-q12 |
| 407 | STARD5 | StAR-related lipid transfer (START) domain containing 5 | 15q26 |
| 408 | STK11 | serine/threonine kinase 11 | 19p13.3 |
| 409 | SYK | spleen tyrosine kinase | 9q22 |
| 410 | TAZ | tafazzin | Xq28 |
| 411 | TBXAS1 | thromboxane A synthase 1 (platelet) | 7q34-q35 |
| 412 | TECR | trans-2,3-enoyl-CoA reductase | 19p13.12 |
| 413 | TFCP2L1 | transcription factor CP2-like 1 | 2q14 |
| 414 | THRSP | thyroid hormone responsive | 11q13.5 |
| 415 | TM7SF2 | transmembrane 7 superfamily member 2 | 11q13 |
| 416 | TNF | tumor necrosis factor | 6p21.3 |
| 417 | TP53RK | TP53 regulating kinase | 20q13.2 |
| 418 | TPI1 | triosephosphate isomerase 1 | 12p13 |
| 419 | TRERF1 | transcriptional regulating factor 1 | 6p21.1-p12.1 |
| 420 | TRIB3 | tribbles homolog 3 (Drosophila) | 20p13-p12.2 |
| 421 | TSPO | translocator protein (18kDa) | 22q13.31 |
| 422 | UGCG | UDP-glucose ceramide glucosyltransferase | 9q31 |
| 423 | WNT4 | wingless-type MMTV integration site family, member 4 | 1p36.23-p35.1 |
| 424 | ZP3 | zona pellucida glycoprotein 3 (sperm receptor) | 7q11.23 |

Table S5. The list of genes in amino acid catabolic process (totally 96 genes).

| **ID** | **Symbol** | **Gene full name** | **Location** |
| --- | --- | --- | --- |
| 1 | A2LD1 | AIG2-like domain 1 | 13q32.3 |
| 2 | AADAT | aminoadipate aminotransferase | 4q33 |
| 3 | AASS | aminoadipate-semialdehyde synthase | 7q31.3 |
| 4 | ABAT | 4-aminobutyrate aminotransferase | 16p13.2 |
| 5 | ACAD8 | acyl-CoA dehydrogenase family, member 8 | 11q25 |
| 6 | ACADL | acyl-CoA dehydrogenase, long chain | 2q34-q35 |
| 7 | ACADSB | acyl-CoA dehydrogenase, short/branched chain | 10q26.13 |
| 8 | ACAT1 | acetyl-CoA acetyltransferase 1 | 11q22.3 |
| 9 | ACHE | acetylcholinesterase | 7q22 |
| 10 | ACMSD | aminocarboxymuconate semialdehyde decarboxylase | 2q21.3 |
| 11 | AFMID | arylformamidase | 17q25.3 |
| 12 | ALDH4A1 | aldehyde dehydrogenase 4 family, member A1 | 1p36 |
| 13 | ALDH5A1 | aldehyde dehydrogenase 5 family, member A1 | 6p22 |
| 14 | ALDH6A1 | aldehyde dehydrogenase 6 family, member A1 | 14q24.3 |
| 15 | ALDH7A1 | aldehyde dehydrogenase 7 family, member A1 | 5q31 |
| 16 | AMDHD1 | amidohydrolase domain containing 1 | 12q23.1 |
| 17 | AMT | aminomethyltransferase | 3p21.2-p21.1 |
| 18 | ARG1 | arginase, liver | 6q23 |
| 19 | ASL | argininosuccinate lyase | 7cen-q11.2 |
| 20 | ASPA | aspartoacylase | 17p13.3 |
| 21 | ASRGL1 | asparaginase like 1 | 11q12.3 |
| 22 | AUH | AU RNA binding protein/enoyl-CoA hydratase | 9q22.31 |
| 23 | BCAT1 | branched chain amino-acid transaminase 1, cytosolic | 12p12.1 |
| 24 | BCAT2 | branched chain amino-acid transaminase 2, mitochondrial | 19q13 |
| 25 | BCKDHA | branched chain keto acid dehydrogenase E1, alpha polypeptide | 19q13.1-q13.2 |
| 26 | BCKDHB | branched chain keto acid dehydrogenase E1, beta polypeptide | 6q14.1 |
| 27 | BCKDK | branched chain ketoacid dehydrogenase kinase | 16p11.2 |
| 28 | C14orf126 | chromosome 14 open reading frame 126 | 14q12 |
| 29 | C1orf69 | chromosome 1 open reading frame 69 | 1q42.13 |
| 30 | C9orf6 | chromosome 9 open reading frame 6 | 9q31.3 |
| 31 | CBS | cystathionine-beta-synthase | 21q22.3 |
| 32 | CCBL1 | cysteine conjugate-beta lyase, cytoplasmic | 9q34.11 |
| 33 | CCBL2 | cysteine conjugate-beta lyase 2 | 1p22.2 |
| 34 | CDO1 | cysteine dioxygenase, type I | 5q23.2 |
| 35 | COLQ | collagen-like tail subunit (single strand of homotrimer) of asymmetric acetylcholinesterase | 3p25 |
| 36 | COMT | catechol-O-methyltransferase | 22q11.21-q11.23|22q11.21 |
| 37 | DBH | dopamine beta-hydroxylase (dopamine beta-monooxygenase) | 9q34 |
| 38 | DBT | dihydrolipoamide branched chain transacylase E2 | 1p31 |
| 39 | DDAH1 | dimethylarginine dimethylaminohydrolase 1 | 1p22 |
| 40 | DDAH2 | dimethylarginine dimethylaminohydrolase 2 | 6p21.3 |
| 41 | DDO | D-aspartate oxidase | 6q21 |
| 42 | DHPS | deoxyhypusine synthase | 19p13 |
| 43 | DLD | dihydrolipoamide dehydrogenase | 7q31-q32 |
| 44 | DLST | dihydrolipoamide S-succinyltransferase (E2 component of 2-oxo-glutarate complex) | 14q24.3 |
| 45 | DMGDH | dimethylglycine dehydrogenase | 5q14.1 |
| 46 | DTD1 | D-tyrosyl-tRNA deacylase 1 homolog (S. cerevisiae) | 20p11.23 |
| 47 | ENOSF1 | enolase superfamily member 1 | 18p11.32 |
| 48 | FAH | fumarylacetoacetate hydrolase (fumarylacetoacetase) | 15q23-q25 |
| 49 | FTCD | formiminotransferase cyclodeaminase | 21q22.3 |
| 50 | GAD1 | glutamate decarboxylase 1 (brain, 67kDa) | 2q31 |
| 51 | GAD2 | glutamate decarboxylase 2 (pancreatic islets and brain, 65kDa) | 10p11.23 |
| 52 | GCDH | glutaryl-CoA dehydrogenase | 19p13.2 |
| 53 | GCSH | glycine cleavage system protein H (aminomethyl carrier) | 16q23.2 |
| 54 | GLDC | glycine dehydrogenase (decarboxylating) | 9p22 |
| 55 | GLS | glutaminase | 2q32-q34 |
| 56 | GLUD1 | glutamate dehydrogenase 1 | 10q23.3 |
| 57 | GLUD2 | glutamate dehydrogenase 2 | Xq24-q25 |
| 58 | GOT1 | glutamic-oxaloacetic transaminase 1, soluble (aspartate aminotransferase 1) | 10q24.1-q25.1 |
| 59 | GOT2 | glutamic-oxaloacetic transaminase 2, mitochondrial (aspartate aminotransferase 2) | 16q21 |
| 60 | GSTZ1 | glutathione transferase zeta 1 | 14q24.3 |
| 61 | HAAO | 3-hydroxyanthranilate 3,4-dioxygenase | 2p21 |
| 62 | HAL | histidine ammonia-lyase | 12q22-q24.1 |
| 63 | HGD | homogentisate 1,2-dioxygenase | 3q13.33 |
| 64 | HIBADH | 3-hydroxyisobutyrate dehydrogenase | 7p15.2 |
| 65 | HIBCH | 3-hydroxyisobutyryl-CoA hydrolase | 2q32.2 |
| 66 | HPD | 4-hydroxyphenylpyruvate dioxygenase | 12q24-qter |
| 67 | HSD17B10 | hydroxysteroid (17-beta) dehydrogenase 10 | Xp11.2 |
| 68 | IDO1 | indoleamine 2,3-dioxygenase 1 | 8p12-p11 |
| 69 | IDO2 | indoleamine 2,3-dioxygenase 2 | 8p11.21 |
| 70 | IVD | isovaleryl-CoA dehydrogenase | 15q14-q15 |
| 71 | KMO | kynurenine 3-monooxygenase (kynurenine 3-hydroxylase) | 1q42-q44 |
| 72 | KYNU | kynureninase (L-kynurenine hydrolase) | 2q22.2 |
| 73 | LRTOMT | leucine rich transmembrane and 0-methyltransferase domain containing | 11q13.4 |
| 74 | MAOA | monoamine oxidase A | Xp11.3 |
| 75 | MCCC1 | methylcrotonoyl-CoA carboxylase 1 (alpha) | 3q27 |
| 76 | MCCC2 | methylcrotonoyl-CoA carboxylase 2 (beta) | 5q12-q13 |
| 77 | NOS1 | nitric oxide synthase 1 (neuronal) | 12q24.2-q24.31 |
| 78 | NOS2 | nitric oxide synthase 2, inducible | 17q11.2-q12 |
| 79 | NOS3 | nitric oxide synthase 3 (endothelial cell) | 7q36 |
| 80 | OGDH | oxoglutarate (alpha-ketoglutarate) dehydrogenase (lipoamide) | 7p14-p13 |
| 81 | PAH | phenylalanine hydroxylase | 12q22-q24.2 |
| 82 | PCBD1 | pterin-4 alpha-carbinolamine dehydratase/dimerization cofactor of hepatocyte nuclear factor 1 alpha | 10q22 |
| 83 | PCYOX1 | prenylcysteine oxidase 1 | 2p13.3 |
| 84 | PCYOX1L | prenylcysteine oxidase 1 like | 5q32 |
| 85 | PDPR | pyruvate dehydrogenase phosphatase regulatory subunit | 16q22.1 |
| 86 | PRODH | proline dehydrogenase (oxidase) 1 | 22q11.21 |
| 87 | PRODH2 | proline dehydrogenase (oxidase) 2 | 19q13.1 |
| 88 | QDPR | quinoid dihydropteridine reductase | 4p15.31 |
| 89 | SARDH | sarcosine dehydrogenase | 9q33-q34 |
| 90 | SDS | serine dehydratase | 12q24.13 |
| 91 | SHMT1 | serine hydroxymethyltransferase 1 (soluble) | 17p11.2 |
| 92 | SLC25A21 | solute carrier family 25 (mitochondrial oxodicarboxylate carrier), member 21 | 14q11.2 |
| 93 | SMOX | spermine oxidase | 20p13 |
| 94 | TAT | tyrosine aminotransferase | 16q22.1 |
| 95 | TDO2 | tryptophan 2,3-dioxygenase | 4q31-q32 |
| 96 | UROC1 | urocanase domain containing 1 | 3q21.3 |

Table S6. The list of genes in amino acid biosynthetic process (totally 426 genes).

| **ID** | **Symbol** | **Gene full name** | **Location** |
| --- | --- | --- | --- |
| 1 | AARS | alanyl-tRNA synthetase | 16q22 |
| 2 | AARS2 | alanyl-tRNA synthetase 2, mitochondrial (putative) | 6p21.1 |
| 3 | AARSD1 | alanyl-tRNA synthetase domain containing 1 | 17q21.31 |
| 4 | ABCF1 | ATP-binding cassette, sub-family F (GCN20), member 1 | 6p21.33 |
| 5 | ACO1 | aconitase 1, soluble | 9p22-q32|9p21.1 |
| 6 | AIMP1 | aminoacyl tRNA synthetase complex-interacting multifunctional protein 1 | 4q24 |
| 7 | AIMP2 | aminoacyl tRNA synthetase complex-interacting multifunctional protein 2 | 7p22 |
| 8 | AKT1 | v-akt murine thymoma viral oncogene homolog 1 | 14q32.32|14q32.32 |
| 9 | ANG | angiogenin, ribonuclease, RNase A family, 5 | 14q11.1-q11.2 |
| 10 | APP | amyloid beta (A4) precursor protein | 21q21.2|21q21.3 |
| 11 | ASMT | acetylserotonin O-methyltransferase | Xp22.3 or Yp11.3 |
| 12 | ATXN2 | ataxin 2 | 12q24.1 |
| 13 | BARHL2 | BarH-like homeobox 2 | 1p22.2 |
| 14 | BCL3 | B-cell CLL/lymphoma 3 | 19q13.1-q13.2 |
| 15 | BOLL | bol, boule-like (Drosophila) | 2q33 |
| 16 | BRIX1 | BRX1, biogenesis of ribosomes, homolog (S. cerevisiae) | 5p13.2 |
| 17 | C12orf65 | chromosome 12 open reading frame 65 | 12q24.31 |
| 18 | C2orf79 | chromosome 2 open reading frame 79 | 2p23.3 |
| 19 | CALR | calreticulin | 19p13.3-p13.2 |
| 20 | CAPRIN1 | cell cycle associated protein 1 | 11p13 |
| 21 | CAPRIN2 | caprin family member 2 | 12p11 |
| 22 | CARS | cysteinyl-tRNA synthetase | 11p15.5 |
| 23 | CARS2 | cysteinyl-tRNA synthetase 2, mitochondrial (putative) | 13q34 |
| 24 | CASC3 | cancer susceptibility candidate 3 | 17q11-q21.3 |
| 25 | CCL5 | chemokine (C-C motif) ligand 5 | 17q11.2-q12 |
| 26 | CD28 | CD28 molecule | 2q33 |
| 27 | CIRBP | cold inducible RNA binding protein | 19p13.3 |
| 28 | COPS5 | COP9 constitutive photomorphogenic homolog subunit 5 (Arabidopsis) | 8q13.1 |
| 29 | CPEB1 | cytoplasmic polyadenylation element binding protein 1 | 15q25.2 |
| 30 | CPEB2 | cytoplasmic polyadenylation element binding protein 2 | 4p15.33 |
| 31 | CTIF | CBP80/20-dependent translation initiation factor | 18q21.1 |
| 32 | CYLD | cylindromatosis (turban tumor syndrome) | 16q12.1 |
| 33 | DALRD3 | DALR anticodon binding domain containing 3 | 3p21.31 |
| 34 | DARS | aspartyl-tRNA synthetase | 2q21.3 |
| 35 | DARS2 | aspartyl-tRNA synthetase 2, mitochondrial | 1q25.1 |
| 36 | DAZ1 | deleted in azoospermia 1 | Yq11.223 |
| 37 | DAZL | deleted in azoospermia-like | 3p24.3 |
| 38 | DDX1 | DEAD (Asp-Glu-Ala-Asp) box polypeptide 1 | 2p24 |
| 39 | DDX25 | DEAD (Asp-Glu-Ala-Asp) box polypeptide 25 | 11q24 |
| 40 | DHPS | deoxyhypusine synthase | 19p13 |
| 41 | DIO2 | deiodinase, iodothyronine, type II | 14q24.2-q24.3 |
| 42 | DNAJC1 | DnaJ (Hsp40) homolog, subfamily C, member 1 | 10p12.31 |
| 43 | DPH1 | DPH1 homolog (S. cerevisiae) | 17p13.3 |
| 44 | EARS2 | glutamyl-tRNA synthetase 2, mitochondrial (putative) | 16p12.2 |
| 45 | EEF1A1 | eukaryotic translation elongation factor 1 alpha 1 | 6q14.1 |
| 46 | EEF1A2 | eukaryotic translation elongation factor 1 alpha 2 | 20q13.3 |
| 47 | EEF1B2 | eukaryotic translation elongation factor 1 beta 2 | 2q33.3 |
| 48 | EEF1D | eukaryotic translation elongation factor 1 delta (guanine nucleotide exchange protein) | 8q24.3 |
| 49 | EEF1E1 | eukaryotic translation elongation factor 1 epsilon 1 | 6p24.3 |
| 50 | EEF1G | eukaryotic translation elongation factor 1 gamma | 11q12.3 |
| 51 | EEF2 | eukaryotic translation elongation factor 2 | 19pter-q12 |
| 52 | EEF2K | eukaryotic elongation factor-2 kinase | 16p12.2 |
| 53 | EEFSEC | eukaryotic elongation factor, selenocysteine-tRNA-specific | 3q21.3 |
| 54 | EIF1 | eukaryotic translation initiation factor 1 | 17q21.2 |
| 55 | EIF1AX | eukaryotic translation initiation factor 1A, X-linked | Xp22.12 |
| 56 | EIF1B | eukaryotic translation initiation factor 1B | 3p22.1 |
| 57 | EIF2A | eukaryotic translation initiation factor 2A, 65kDa | 3q25.1 |
| 58 | EIF2AK1 | eukaryotic translation initiation factor 2-alpha kinase 1 | 7p22 |
| 59 | EIF2AK3 | eukaryotic translation initiation factor 2-alpha kinase 3 | 2p12 |
| 60 | EIF2AK4 | eukaryotic translation initiation factor 2 alpha kinase 4 | 15q15.1 |
| 61 | EIF2B1 | eukaryotic translation initiation factor 2B, subunit 1 alpha, 26kDa | 12q24.31 |
| 62 | EIF2B2 | eukaryotic translation initiation factor 2B, subunit 2 beta, 39kDa | 14q24.3 |
| 63 | EIF2B3 | eukaryotic translation initiation factor 2B, subunit 3 gamma, 58kDa | 1p34.1 |
| 64 | EIF2B4 | eukaryotic translation initiation factor 2B, subunit 4 delta, 67kDa | 2p23.3 |
| 65 | EIF2B5 | eukaryotic translation initiation factor 2B, subunit 5 epsilon, 82kDa | 3q27.1 |
| 66 | EIF2C1 | eukaryotic translation initiation factor 2C, 1 | 1p34.3 |
| 67 | EIF2C2 | eukaryotic translation initiation factor 2C, 2 | 8q24 |
| 68 | EIF2C3 | eukaryotic translation initiation factor 2C, 3 | 1p34.3 |
| 69 | EIF2C4 | eukaryotic translation initiation factor 2C, 4 | 1p34.3 |
| 70 | EIF2D | eukaryotic translation initiation factor 2D | 1q32.1 |
| 71 | EIF2S1 | eukaryotic translation initiation factor 2, subunit 1 alpha, 35kDa | 14q23.3 |
| 72 | EIF2S2 | eukaryotic translation initiation factor 2, subunit 2 beta, 38kDa | 20pter-q12 |
| 73 | EIF2S3 | eukaryotic translation initiation factor 2, subunit 3 gamma, 52kDa | Xp22.2-p22.1 |
| 74 | EIF3A | eukaryotic translation initiation factor 3, subunit A | 10q26 |
| 75 | EIF3B | eukaryotic translation initiation factor 3, subunit B | 7p22.3 |
| 76 | EIF3C | eukaryotic translation initiation factor 3, subunit C | 16p11.2 |
| 77 | EIF3D | eukaryotic translation initiation factor 3, subunit D | 22q13.1 |
| 78 | EIF3E | eukaryotic translation initiation factor 3, subunit E | 8q22-q23 |
| 79 | EIF3F | eukaryotic translation initiation factor 3, subunit F | 11p15.4 |
| 80 | EIF3G | eukaryotic translation initiation factor 3, subunit G | 19p13.2 |
| 81 | EIF3H | eukaryotic translation initiation factor 3, subunit H | 8q24.11 |
| 82 | EIF3I | eukaryotic translation initiation factor 3, subunit I | 1p34.1 |
| 83 | EIF3J | eukaryotic translation initiation factor 3, subunit J | 15q21.1 |
| 84 | EIF3K | eukaryotic translation initiation factor 3, subunit K | 19q13.2 |
| 85 | EIF3L | eukaryotic translation initiation factor 3, subunit L | 22q |
| 86 | EIF3M | eukaryotic translation initiation factor 3, subunit M | 11p13 |
| 87 | EIF4A1 | eukaryotic translation initiation factor 4A1 | 17p13 |
| 88 | EIF4A2 | eukaryotic translation initiation factor 4A2 | 3q28 |
| 89 | EIF4A3 | eukaryotic translation initiation factor 4A3 | 17q25.3 |
| 90 | EIF4B | eukaryotic translation initiation factor 4B | 12q13.13 |
| 91 | EIF4E | eukaryotic translation initiation factor 4E | 4q21-q25 |
| 92 | EIF4E1B | eukaryotic translation initiation factor 4E family member 1B | 5q35.2 |
| 93 | EIF4E2 | eukaryotic translation initiation factor 4E family member 2 | 2q37.1 |
| 94 | EIF4E3 | eukaryotic translation initiation factor 4E family member 3 | 3p14 |
| 95 | EIF4EBP1 | eukaryotic translation initiation factor 4E binding protein 1 | 8p12 |
| 96 | EIF4EBP2 | eukaryotic translation initiation factor 4E binding protein 2 | 10q21-q22 |
| 97 | EIF4EBP3 | eukaryotic translation initiation factor 4E binding protein 3 | 5q31.3 |
| 98 | EIF4G1 | eukaryotic translation initiation factor 4 gamma, 1 | 3q27-qter |
| 99 | EIF4G2 | eukaryotic translation initiation factor 4 gamma, 2 | 11p15 |
| 100 | EIF4G3 | eukaryotic translation initiation factor 4 gamma, 3 | 1p36.12 |
| 101 | EIF4H | eukaryotic translation initiation factor 4H | 7q11.23 |
| 102 | EIF5 | eukaryotic translation initiation factor 5 | 14q32.32 |
| 103 | EIF5A | eukaryotic translation initiation factor 5A | 17p13-p12 |
| 104 | EIF5A2 | eukaryotic translation initiation factor 5A2 | 3q26.2 |
| 105 | EIF5AL1 | eukaryotic translation initiation factor 5A-like 1 | 10q22.3 |
| 106 | EIF5B | eukaryotic translation initiation factor 5B | 2q11.2 |
| 107 | EP300 | E1A binding protein p300 | 22q13.2 |
| 108 | EPRS | glutamyl-prolyl-tRNA synthetase | 1q41 |
| 109 | ETF1 | eukaryotic translation termination factor 1 | 5q31.1 |
| 110 | FAM129A | family with sequence similarity 129, member A | 1q25 |
| 111 | FARS2 | phenylalanyl-tRNA synthetase 2, mitochondrial | 6p25.1 |
| 112 | FARSA | phenylalanyl-tRNA synthetase, alpha subunit | 19p13.2 |
| 113 | FARSB | phenylalanyl-tRNA synthetase, beta subunit | 2q36.1 |
| 114 | FAU | Finkel-Biskis-Reilly murine sarcoma virus (FBR-MuSV) ubiquitously expressed | 11q13 |
| 115 | FDXACB1 | ferredoxin-fold anticodon binding domain containing 1 | 11q23.1 |
| 116 | FMR1 | fragile X mental retardation 1 | Xq27.3 |
| 117 | GARS | glycyl-tRNA synthetase | 7p15 |
| 118 | GATC | glutamyl-tRNA(Gln) amidotransferase, subunit C homolog (bacterial) | 12q24.31 |
| 119 | GCN1L1 | GCN1 general control of amino-acid synthesis 1-like 1 (yeast) | 12q24.2 |
| 120 | GFM1 | G elongation factor, mitochondrial 1 | 3q25 |
| 121 | GFM2 | G elongation factor, mitochondrial 2 | 5q13 |
| 122 | GSPT1 | G1 to S phase transition 1 | 16p13.1 |
| 123 | GSPT2 | G1 to S phase transition 2 | Xp11.22 |
| 124 | GTF2H2 | general transcription factor IIH, polypeptide 2, 44kDa | 5q13.2 |
| 125 | GTF2H3 | general transcription factor IIH, polypeptide 3, 34kDa | 12q24.31 |
| 126 | GUF1 | GUF1 GTPase homolog (S. cerevisiae) | 4p12 |
| 127 | HARS | histidyl-tRNA synthetase | 5q31.3 |
| 128 | HARS2 | histidyl-tRNA synthetase 2, mitochondrial (putative) | 5q31.3 |
| 129 | HBS1L | HBS1-like (S. cerevisiae) | 6q23.3 |
| 130 | HRSP12 | heat-responsive protein 12 | 8q22 |
| 131 | HSPB1 | heat shock 27kDa protein 1 | 7q11.23 |
| 132 | IARS | isoleucyl-tRNA synthetase | 9q21 |
| 133 | IARS2 | isoleucyl-tRNA synthetase 2, mitochondrial | 1q41 |
| 134 | ICT1 | immature colon carcinoma transcript 1 | 17q25.1 |
| 135 | IGF2BP1 | insulin-like growth factor 2 mRNA binding protein 1 | 17q21.32 |
| 136 | IGF2BP2 | insulin-like growth factor 2 mRNA binding protein 2 | 3q27.2 |
| 137 | IGF2BP3 | insulin-like growth factor 2 mRNA binding protein 3 | 7p11 |
| 138 | IGFBP5 | insulin-like growth factor binding protein 5 | 2q33-q36 |
| 139 | IGHMBP2 | immunoglobulin mu binding protein 2 | 11q13.3 |
| 140 | IL6 | interleukin 6 (interferon, beta 2) | 7p21 |
| 141 | IMP4 | IMP4, U3 small nucleolar ribonucleoprotein, homolog (yeast) | 2q21.1 |
| 142 | IMPACT | Impact homolog (mouse) | 18q11.2-q12.1 |
| 143 | IREB2 | iron-responsive element binding protein 2 | 15q25.1 |
| 144 | KARS | lysyl-tRNA synthetase | 16q23.1 |
| 145 | KRT7 | keratin 7 | 12q12-q13 |
| 146 | LARS | leucyl-tRNA synthetase | 5q32 |
| 147 | LARS2 | leucyl-tRNA synthetase 2, mitochondrial | 3p21.3 |
| 148 | LIN28A | lin-28 homolog A (C. elegans) | 1p36.11 |
| 149 | LRRC47 | leucine rich repeat containing 47 | 1p36.32 |
| 150 | LSM14A | LSM14A, SCD6 homolog A (S. cerevisiae) | 19q13.11 |
| 151 | LSM14B | LSM14B, SCD6 homolog B (S. cerevisiae) | 20q13.33 |
| 152 | MAGOH | mago-nashi homolog, proliferation-associated (Drosophila) | 1p32.3 |
| 153 | MAPK1 | mitogen-activated protein kinase 1 | 22q11.2|22q11.21 |
| 154 | MARS | methionyl-tRNA synthetase | 12q13.2 |
| 155 | MARS2 | methionyl-tRNA synthetase 2, mitochondrial | 2q33.1 |
| 156 | METAP1 | methionyl aminopeptidase 1 | 4q23 |
| 157 | METT11D1 | methyltransferase 11 domain containing 1 | 14q11.2 |
| 158 | MIF4GD | MIF4G domain containing | 17q25.1 |
| 159 | MKNK1 | MAP kinase interacting serine/threonine kinase 1 | 1p33 |
| 160 | MKNK2 | MAP kinase interacting serine/threonine kinase 2 | 19p13.3 |
| 161 | MRP63 | mitochondrial ribosomal protein 63 | 13q12.11 |
| 162 | MRPL1 | mitochondrial ribosomal protein L1 | 4q21.1 |
| 163 | MRPL10 | mitochondrial ribosomal protein L10 | 17q21.32 |
| 164 | MRPL11 | mitochondrial ribosomal protein L11 | 11q13.3 |
| 165 | MRPL12 | mitochondrial ribosomal protein L12 | 17q25 |
| 166 | MRPL13 | mitochondrial ribosomal protein L13 | 8q22.1-q22.3 |
| 167 | MRPL14 | mitochondrial ribosomal protein L14 | 6p21.3 |
| 168 | MRPL15 | mitochondrial ribosomal protein L15 | 8q11.2-q13 |
| 169 | MRPL16 | mitochondrial ribosomal protein L16 | 11q12.1 |
| 170 | MRPL17 | mitochondrial ribosomal protein L17 | 11p15.5-p15.4 |
| 171 | MRPL18 | mitochondrial ribosomal protein L18 | 6q25.3 |
| 172 | MRPL19 | mitochondrial ribosomal protein L19 | 2q11.1-q11.2 |
| 173 | MRPL2 | mitochondrial ribosomal protein L2 | 6p21.3 |
| 174 | MRPL20 | mitochondrial ribosomal protein L20 | 1p36.3-p36.2 |
| 175 | MRPL21 | mitochondrial ribosomal protein L21 | 11q13.3 |
| 176 | MRPL22 | mitochondrial ribosomal protein L22 | 5q33.2 |
| 177 | MRPL23 | mitochondrial ribosomal protein L23 | 11p15.5 |
| 178 | MRPL24 | mitochondrial ribosomal protein L24 | 1q21-q22 |
| 179 | MRPL27 | mitochondrial ribosomal protein L27 | 17q21.3-q22 |
| 180 | MRPL28 | mitochondrial ribosomal protein L28 | 16p13.3 |
| 181 | MRPL3 | mitochondrial ribosomal protein L3 | 3q21-q23 |
| 182 | MRPL30 | mitochondrial ribosomal protein L30 | 2q11.2 |
| 183 | MRPL32 | mitochondrial ribosomal protein L32 | 7p14 |
| 184 | MRPL33 | mitochondrial ribosomal protein L33 | 2p21 |
| 185 | MRPL34 | mitochondrial ribosomal protein L34 | 19p13.1 |
| 186 | MRPL35 | mitochondrial ribosomal protein L35 | 2p11.2 |
| 187 | MRPL36 | mitochondrial ribosomal protein L36 | 5p15.3 |
| 188 | MRPL37 | mitochondrial ribosomal protein L37 | 1p32.1 |
| 189 | MRPL4 | mitochondrial ribosomal protein L4 | 19p13.2 |
| 190 | MRPL41 | mitochondrial ribosomal protein L41 | 9q34.3 |
| 191 | MRPL42 | mitochondrial ribosomal protein L42 | 12q22 |
| 192 | MRPL43 | mitochondrial ribosomal protein L43 | 10q24.31 |
| 193 | MRPL45 | mitochondrial ribosomal protein L45 | 17q21.2 |
| 194 | MRPL47 | mitochondrial ribosomal protein L47 | 3q26.33 |
| 195 | MRPL48 | mitochondrial ribosomal protein L48 | 11q13.4 |
| 196 | MRPL49 | mitochondrial ribosomal protein L49 | 11q13 |
| 197 | MRPL51 | mitochondrial ribosomal protein L51 | 12p13.3-p13.1 |
| 198 | MRPL52 | mitochondrial ribosomal protein L52 | 14q11.2 |
| 199 | MRPL55 | mitochondrial ribosomal protein L55 | 1q42.13 |
| 200 | MRPL9 | mitochondrial ribosomal protein L9 | 1q21 |
| 201 | MRPS10 | mitochondrial ribosomal protein S10 | 6p21.1 |
| 202 | MRPS11 | mitochondrial ribosomal protein S11 | 15q25 |
| 203 | MRPS12 | mitochondrial ribosomal protein S12 | 19q13.1-q13.2 |
| 204 | MRPS14 | mitochondrial ribosomal protein S14 | 1q25.1 |
| 205 | MRPS15 | mitochondrial ribosomal protein S15 | 1p34.3 |
| 206 | MRPS16 | mitochondrial ribosomal protein S16 | 10q22.1 |
| 207 | MRPS17 | mitochondrial ribosomal protein S17 | 7p11 |
| 208 | MRPS18A | mitochondrial ribosomal protein S18A | 6p21.3 |
| 209 | MRPS18B | mitochondrial ribosomal protein S18B | 6p21.3 |
| 210 | MRPS18C | mitochondrial ribosomal protein S18C | 4q21.23 |
| 211 | MRPS2 | mitochondrial ribosomal protein S2 | 9q34 |
| 212 | MRPS21 | mitochondrial ribosomal protein S21 | 1q21 |
| 213 | MRPS23 | mitochondrial ribosomal protein S23 | 17q22-q23 |
| 214 | MRPS24 | mitochondrial ribosomal protein S24 | 7p14 |
| 215 | MRPS25 | mitochondrial ribosomal protein S25 | 3p25 |
| 216 | MRPS30 | mitochondrial ribosomal protein S30 | 5q11 |
| 217 | MRPS33 | mitochondrial ribosomal protein S33 | 7q34 |
| 218 | MRPS36 | mitochondrial ribosomal protein S36 | 5q13.2 |
| 219 | MRPS5 | mitochondrial ribosomal protein S5 | 2p11.2-q11.2 |
| 220 | MRPS6 | mitochondrial ribosomal protein S6 | 21q22.11 |
| 221 | MRPS7 | mitochondrial ribosomal protein S7 | 17q25 |
| 222 | MRPS9 | mitochondrial ribosomal protein S9 | 2q12.1 |
| 223 | MRRF | mitochondrial ribosome recycling factor | 9q33.2 |
| 224 | MTIF2 | mitochondrial translational initiation factor 2 | 2p16.1 |
| 225 | MTIF3 | mitochondrial translational initiation factor 3 | 13q12.2 |
| 226 | MTOR | mechanistic target of rapamycin (serine/threonine kinase) | 1p36.2 |
| 227 | MTPN | myotrophin | 7q33 |
| 228 | MTRF1 | mitochondrial translational release factor 1 | 13q14.1-q14.3 |
| 229 | MTRF1L | mitochondrial translational release factor 1-like | 6q25-q26 |
| 230 | NACA | nascent polypeptide-associated complex alpha subunit | 12q23-q24.1 |
| 231 | NANOS1 | nanos homolog 1 (Drosophila) | 10q26.11 |
| 232 | NANOS2 | nanos homolog 2 (Drosophila) | 19q13.32 |
| 233 | NANOS3 | nanos homolog 3 (Drosophila) | 19p13.13 |
| 234 | NARS | asparaginyl-tRNA synthetase | 18q21.31 |
| 235 | NARS2 | asparaginyl-tRNA synthetase 2, mitochondrial (putative) | 11q14.1 |
| 236 | NCBP1 | nuclear cap binding protein subunit 1, 80kDa | 9q34.1 |
| 237 | NCBP2 | nuclear cap binding protein subunit 2, 20kDa | 3q29 |
| 238 | NCK1 | NCK adaptor protein 1 | 3q21 |
| 239 | NCK2 | NCK adaptor protein 2 | 2q12 |
| 240 | NCOA5 | nuclear receptor coactivator 5 | 20q12-q13.12 |
| 241 | NDUFA13 | NADH dehydrogenase (ubiquinone) 1 alpha subcomplex, 13 | 19p13.2 |
| 242 | NGDN | neuroguidin, EIF4E binding protein | 14q11.2 |
| 243 | P0C6E6 | ribosomal protein L36 pseudogene 14 | 9q31.3 |
| 244 | PA2G4 | proliferation-associated 2G4, 38kDa | 12q13.2 |
| 245 | PABPC1 | poly(A) binding protein, cytoplasmic 1 | 8q22.2-q23 |
| 246 | PABPC4 | poly(A) binding protein, cytoplasmic 4 (inducible form) | 1p34.2 |
| 247 | PAIP1 | poly(A) binding protein interacting protein 1 | 5p12 |
| 248 | PAIP2 | poly(A) binding protein interacting protein 2 | 5q31.2 |
| 249 | PAIP2B | poly(A) binding protein interacting protein 2B | 2p13.3 |
| 250 | PARS2 | prolyl-tRNA synthetase 2, mitochondrial (putative) | 1p32.2 |
| 251 | PATL2 | protein associated with topoisomerase II homolog 2 (yeast) | 15q21.1 |
| 252 | PDF | peptide deformylase (mitochondrial) | 16q22.1 |
| 253 | PELO | pelota homolog (Drosophila) | 5q11.2 |
| 254 | PET112L | PET112-like (yeast) | 4q27-q28 |
| 255 | PIWIL1 | piwi-like 1 (Drosophila) | 12q24.33 |
| 256 | PIWIL2 | piwi-like 2 (Drosophila) | 8p21.3 |
| 257 | PIWIL3 | piwi-like 3 (Drosophila) | 22q11.23 |
| 258 | PIWIL4 | piwi-like 4 (Drosophila) | 11q21 |
| 259 | PML | promyelocytic leukemia | 15q22 |
| 260 | POLDIP3 | polymerase (DNA-directed), delta interacting protein 3 | 22q13.2 |
| 261 | POLG2 | polymerase (DNA directed), gamma 2, accessory subunit | 17q |
| 262 | PPA1 | pyrophosphatase (inorganic) 1 | 10q11.1-q24 |
| 263 | PPA2 | pyrophosphatase (inorganic) 2 | 4q25 |
| 264 | PPP1R15A | protein phosphatase 1, regulatory (inhibitor) subunit 15A | 19q13.2 |
| 265 | PPP1R15B | protein phosphatase 1, regulatory (inhibitor) subunit 15B | 1q32.1 |
| 266 | PRG3 | proteoglycan 3 | 11q12 |
| 267 | PRKCA | protein kinase C, alpha | 17q22-q23.2 |
| 268 | PSTK | phosphoseryl-tRNA kinase | 10q26.13 |
| 269 | PTK2B | PTK2B protein tyrosine kinase 2 beta | 8p21.1 |
| 270 | PTRH1 | peptidyl-tRNA hydrolase 1 homolog (S. cerevisiae) | 9q34.11 |
| 271 | PTRH2 | peptidyl-tRNA hydrolase 2 | 17q23.1 |
| 272 | PUM1 | pumilio homolog 1 (Drosophila) | 1p35.2 |
| 273 | PUM2 | pumilio homolog 2 (Drosophila) | 2p22-p21 |
| 274 | Q6NVV1 | ribosomal protein L13a pseudogene 3 | 14q22.3 |
| 275 | QARS | glutaminyl-tRNA synthetase | 3p21.31 |
| 276 | QKI | quaking homolog, KH domain RNA binding (mouse) | 6q26 |
| 277 | QRSL1 | glutaminyl-tRNA synthase (glutamine-hydrolyzing)-like 1 | 6q21 |
| 278 | RARS | arginyl-tRNA synthetase | 5q35.1 |
| 279 | RARS2 | arginyl-tRNA synthetase 2, mitochondrial | 6q16.1 |
| 280 | RBM3 | RNA binding motif (RNP1, RRM) protein 3 | Xp11.2 |
| 281 | RBM8A | RNA binding motif protein 8A | 1q12 |
| 282 | RHOC | ras homolog gene family, member C | 1p13.1 |
| 283 | RPF1 | ribosome production factor 1 homolog (S. cerevisiae) | 1p22.3 |
| 284 | RPL10 | ribosomal protein L10 | Xq28 |
| 285 | RPL10A | ribosomal protein L10a | 6p21.31 |
| 286 | RPL10L | ribosomal protein L10-like | 14q13-q21 |
| 287 | RPL11 | ribosomal protein L11 | 1p36.1-p35 |
| 288 | RPL12 | ribosomal protein L12 | 9q34 |
| 289 | RPL13 | ribosomal protein L13 | 16q24.3|17p11.2 |
| 290 | RPL13A | ribosomal protein L13a | 19q13.3 |
| 291 | RPL14 | ribosomal protein L14 | 3p22-p21.2 |
| 292 | RPL15 | ribosomal protein L15 | 3p24.2 |
| 293 | RPL17 | ribosomal protein L17 | 18q21 |
| 294 | RPL18 | ribosomal protein L18 | 19q13 |
| 295 | RPL18A | ribosomal protein L18a | 19p13 |
| 296 | RPL19 | ribosomal protein L19 | 17q11.2-q12 |
| 297 | RPL21 | ribosomal protein L21 | 13q12.2 |
| 298 | RPL22 | ribosomal protein L22 | 1p36.31 |
| 299 | RPL22L1 | ribosomal protein L22-like 1 | 3q26.2 |
| 300 | RPL23 | ribosomal protein L23 | 17q |
| 301 | RPL23A | ribosomal protein L23a | 17q11 |
| 302 | RPL24 | ribosomal protein L24 | 3q12 |
| 303 | RPL26 | ribosomal protein L26 | 17p13 |
| 304 | RPL26L1 | ribosomal protein L26-like 1 | 5q35.1 |
| 305 | RPL27 | ribosomal protein L27 | 17q21.1-q21.2 |
| 306 | RPL27A | ribosomal protein L27a | 11p15 |
| 307 | RPL28 | ribosomal protein L28 | 19q13.4 |
| 308 | RPL29 | ribosomal protein L29 | 3p21.3-p21.2 |
| 309 | RPL3 | ribosomal protein L3 | 22q13 |
| 310 | RPL30 | ribosomal protein L30 | 8q22 |
| 311 | RPL31 | ribosomal protein L31 | 2q11.2 |
| 312 | RPL32 | ribosomal protein L32 | 3p25-p24 |
| 313 | RPL34 | ribosomal protein L34 | 4q25 |
| 314 | RPL35 | ribosomal protein L35 | 9q34.1 |
| 315 | RPL35A | ribosomal protein L35a | 3q29-qter |
| 316 | RPL36 | ribosomal protein L36 | 19p13.3 |
| 317 | RPL36A | ribosomal protein L36a | Xq22.1 |
| 318 | RPL36AL | ribosomal protein L36a-like | 14q21 |
| 319 | RPL37 | ribosomal protein L37 | 5p13 |
| 320 | RPL37A | ribosomal protein L37a | 2q35 |
| 321 | RPL37L | ribosomal protein L37 | 5p13 |
| 322 | RPL38 | ribosomal protein L38 | 17q25.1 |
| 323 | RPL39 | ribosomal protein L39 | Xq24 |
| 324 | RPL39L | ribosomal protein L39-like | 3q27 |
| 325 | RPL39P5 | ribosomal protein L39 pseudogene 5 | 3q22.1 |
| 326 | RPL3L | ribosomal protein L3-like | 16p13.3 |
| 327 | RPL4 | ribosomal protein L4 | 15q22 |
| 328 | RPL41 | ribosomal protein L41 | 12q13 |
| 329 | RPL5 | ribosomal protein L5 | 1p22.1 |
| 330 | RPL6 | ribosomal protein L6 | 12q24.1 |
| 331 | RPL7 | ribosomal protein L7 | 8q21.11 |
| 332 | RPL7A | ribosomal protein L7a | 9q34 |
| 333 | RPL7L1 | ribosomal protein L7-like 1 | 6p21.1 |
| 334 | RPL8 | ribosomal protein L8 | 8q24.3 |
| 335 | RPL9P9 | ribosomal protein L9 pseudogene 9 | 15q25.2 |
| 336 | RPLP0 | ribosomal protein, large, P0 | 12q24.2 |
| 337 | RPLP0P6 | ribosomal protein, large, P0 pseudogene 6 | 2p22.1 |
| 338 | RPLP1 | ribosomal protein, large, P1 | 15q22 |
| 339 | RPLP2 | ribosomal protein, large, P2 | 11p15.5 |
| 340 | RPS10 | ribosomal protein S10 | 6p21.31 |
| 341 | RPS11 | ribosomal protein S11 | 19q13.3 |
| 342 | RPS12 | ribosomal protein S12 | 6q23.2 |
| 343 | RPS13 | ribosomal protein S13 | 11p15 |
| 344 | RPS14 | ribosomal protein S14 | 5q31-q33 |
| 345 | RPS15 | ribosomal protein S15 | 19p13.3 |
| 346 | RPS15A | ribosomal protein S15a | 16p |
| 347 | RPS16 | ribosomal protein S16 | 19q13.1 |
| 348 | RPS17 | ribosomal protein S17 | 15q |
| 349 | RPS18 | ribosomal protein S18 | 6p21.3 |
| 350 | RPS19 | ribosomal protein S19 | 19q13.2 |
| 351 | RPS2 | ribosomal protein S2 | 16p13.3 |
| 352 | RPS20 | ribosomal protein S20 | 8q12 |
| 353 | RPS21 | ribosomal protein S21 | 20q13.3 |
| 354 | RPS23 | ribosomal protein S23 | 5q14.2 |
| 355 | RPS24 | ribosomal protein S24 | 10q22 |
| 356 | RPS25 | ribosomal protein S25 | 11q23.3 |
| 357 | RPS26 | ribosomal protein S26 | 12q13 |
| 358 | RPS26P11 | ribosomal protein S26 pseudogene 11 | Xq13.1 |
| 359 | RPS27 | ribosomal protein S27 | 1q21 |
| 360 | RPS27A | ribosomal protein S27a | 2p16 |
| 361 | RPS27L | ribosomal protein S27-like | 15q22.2 |
| 362 | RPS28 | ribosomal protein S28 | 19p13.2 |
| 363 | RPS29 | ribosomal protein S29 | 14q |
| 364 | RPS3 | ribosomal protein S3 | 11q13.3-q13.5 |
| 365 | RPS3A | ribosomal protein S3A | 4q31.2-q31.3 |
| 366 | RPS4X | ribosomal protein S4, X-linked | Xq13.1 |
| 367 | RPS4Y1 | ribosomal protein S4, Y-linked 1 | Yp11.3 |
| 368 | RPS4Y2 | ribosomal protein S4, Y-linked 2 | Yq11.223 |
| 369 | RPS5 | ribosomal protein S5 | 19q13.4 |
| 370 | RPS6 | ribosomal protein S6 | 9p21 |
| 371 | RPS6KB1 | ribosomal protein S6 kinase, 70kDa, polypeptide 1 | 17q23.1 |
| 372 | RPS6KB2 | ribosomal protein S6 kinase, 70kDa, polypeptide 2 | 11q13.2 |
| 373 | RPS7 | ribosomal protein S7 | 2p25 |
| 374 | RPS8 | ribosomal protein S8 | 1p34.1-p32 |
| 375 | RPS9 | ribosomal protein S9 | 19q13.4 |
| 376 | RPSA | ribosomal protein SA | 3p22.2 |
| 377 | RRBP1 | ribosome binding protein 1 homolog 180kDa (dog) | 20p12 |
| 378 | RSL1D1 | ribosomal L1 domain containing 1 | 16p13.13 |
| 379 | RSL24D1 | ribosomal L24 domain containing 1 | 15q21 |
| 380 | SAMD4A | sterile alpha motif domain containing 4A | 14q22.2 |
| 381 | SARNP | SAP domain containing ribonucleoprotein | 12q13.2 |
| 382 | SARS | seryl-tRNA synthetase | 1p13.3 |
| 383 | SARS2 | seryl-tRNA synthetase 2, mitochondrial | 19q13.2 |
| 384 | SBDS | Shwachman-Bodian-Diamond syndrome | 7q11.21 |
| 385 | SECISBP2 | SECIS binding protein 2 | 9q22.2 |
| 386 | SELT | - | 3q25.1 |
| 387 | SEPSECS | Sep (O-phosphoserine) tRNA:Sec (selenocysteine) tRNA synthase | 4p15.2 |
| 388 | SERP1 | stress-associated endoplasmic reticulum protein 1 | 3q25.1 |
| 389 | SOX11 | SRY (sex determining region Y)-box 11 | 2p25 |
| 390 | SRP14 | signal recognition particle 14kDa (homologous Alu RNA binding protein) | 15q22 |
| 391 | SRP9 | signal recognition particle 9kDa | 1q42.12 |
| 392 | TACO1 | translational activator of mitochondrially encoded cytochrome c oxidase I | 17q23.3 |
| 393 | TARBP2 | TAR (HIV-1) RNA binding protein 2 | 12q12-q13 |
| 394 | TARS | threonyl-tRNA synthetase | 5p13.2 |
| 395 | TARS2 | threonyl-tRNA synthetase 2, mitochondrial (putative) | 1q21.3 |
| 396 | TARSL2 | threonyl-tRNA synthetase-like 2 | 15q26.3 |
| 397 | THBS1 | thrombospondin 1 | 15q15 |
| 398 | TIA1 | TIA1 cytotoxic granule-associated RNA binding protein | 2p13 |
| 399 | TICRR | chromosome 15 open reading frame 42 | 15q26.1 |
| 400 | TNF | tumor necrosis factor | 6p21.3 |
| 401 | TNIP1 | TNFAIP3 interacting protein 1 | 5q32-q33.1 |
| 402 | TNRC6A | trinucleotide repeat containing 6A | 16p11.2 |
| 403 | TNRC6B | trinucleotide repeat containing 6B | 22q13.1 |
| 404 | TNRC6C | trinucleotide repeat containing 6C | 17q25.3 |
| 405 | TPR | translocated promoter region (to activated MET oncogene) | 1q25 |
| 406 | TRMT6 | tRNA methyltransferase 6 homolog (S. cerevisiae) | 20p12.3 |
| 407 | TRNAU1AP | tRNA selenocysteine 1 associated protein 1 | 1p35.3 |
| 408 | TSC1 | tuberous sclerosis 1 | 9q34 |
| 409 | TSFM | Ts translation elongation factor, mitochondrial | 12q14.1 |
| 410 | TUFM | Tu translation elongation factor, mitochondrial | 16p11.2 |
| 411 | UBA52 | ubiquitin A-52 residue ribosomal protein fusion product 1 | 19p13.1-p12 |
| 412 | UCN | urocortin | 2p23-p21 |
| 413 | UHMK1 | U2AF homology motif (UHM) kinase 1 | 1q23.3 |
| 414 | UPF1 | UPF1 regulator of nonsense transcripts homolog (yeast) | 19p13.2-p13.11 |
| 415 | UPF3A | UPF3 regulator of nonsense transcripts homolog A (yeast) | 13q34 |
| 416 | UPF3B | UPF3 regulator of nonsense transcripts homolog B (yeast) | Xq25-q26 |
| 417 | VARS | valyl-tRNA synthetase | 6p21.3 |
| 418 | VARS2 | valyl-tRNA synthetase 2, mitochondrial (putative) | 6p21.33 |
| 419 | WARS | tryptophanyl-tRNA synthetase | 14q32.31 |
| 420 | WARS2 | tryptophanyl tRNA synthetase 2, mitochondrial | 1p12 |
| 421 | WIBG | within bgcn homolog (Drosophila) | 12q13.2 |
| 422 | WT1 | Wilms tumor 1 | 11p13 |
| 423 | YARS | tyrosyl-tRNA synthetase | 1p35.1 |
| 424 | YARS2 | tyrosyl-tRNA synthetase 2, mitochondrial | 12p11.21 |
| 425 | YBX2 | Y box binding protein 2 | 17p13.1 |
| 426 | ZFP36L1 | zinc finger protein 36, C3H type-like 1 | 14q22-q24 |
