# Supplementary material for: Significantly fewer protein functional changing variants for lipid metabolism in Africans than in Europeans
Source: J Transl Med. 2013 Mar 20;11:67. doi: 10.1186/1479-5876-11-67 (PMC3610238; doi:10.1186/1479-5876-11-67)
Supplement: Additional file 2 — Distribution of R’ [the difference of mean R between two populations] in permutation test and the observed and background genetic risk (Num) for Hapmap dataset in carbohydrate, lipid and amino acid metabolic (catabolic and biosynthetic) processes. [file 1479-5876-11-67-S2.doc]

Significantly fewer protein changing variants for lipid metabolism in Africans than in Europeans

Cheng Xue^*, **^, Xiaoming Liu^*^, Yun Gong^*^, Yuhai Zhao^§^ and Yun-Xin Fu^*,§§^

^*^ Human Genetics Center, University of Texas Health Science Center at Houston, Houston, Texas, USA

^**^ GuangDong Institute for Monitoring Laboratory Animals, China

^§^ Medical School, University of Texas Health Science Center at Houston, Houston, Texas, USA

^§§^ Laboratory for Conservation and Utilization of Bio-resources, Yunnan University, China

Supplementary information – Figures S1-S15


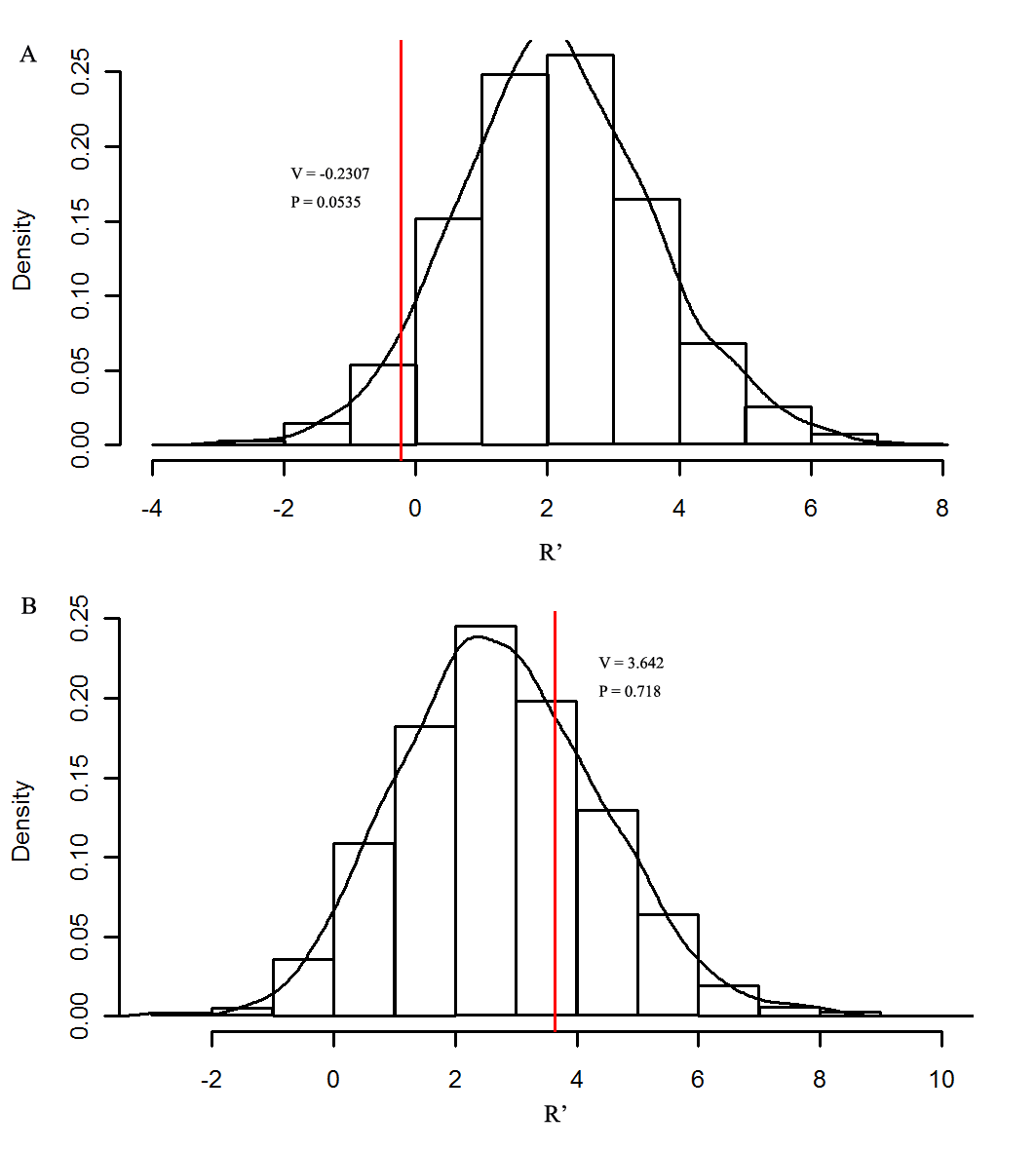


Figure S1. The distribution of R’ [the difference of mean R between Africans (YRI) and Europeans(CEU)] in permutation test and the difference of mean R for carbohydrate metabolism between African and European (V = R’_African-European_ = mean R_African_ – mean R_European_, is shown as the red line in figure) in 1000 Genome data. In permutation test, the re-sampling process repeats 2000 times. In subplot A are results in the catabolic process; and in subplot B are results in the biosynthetic process.


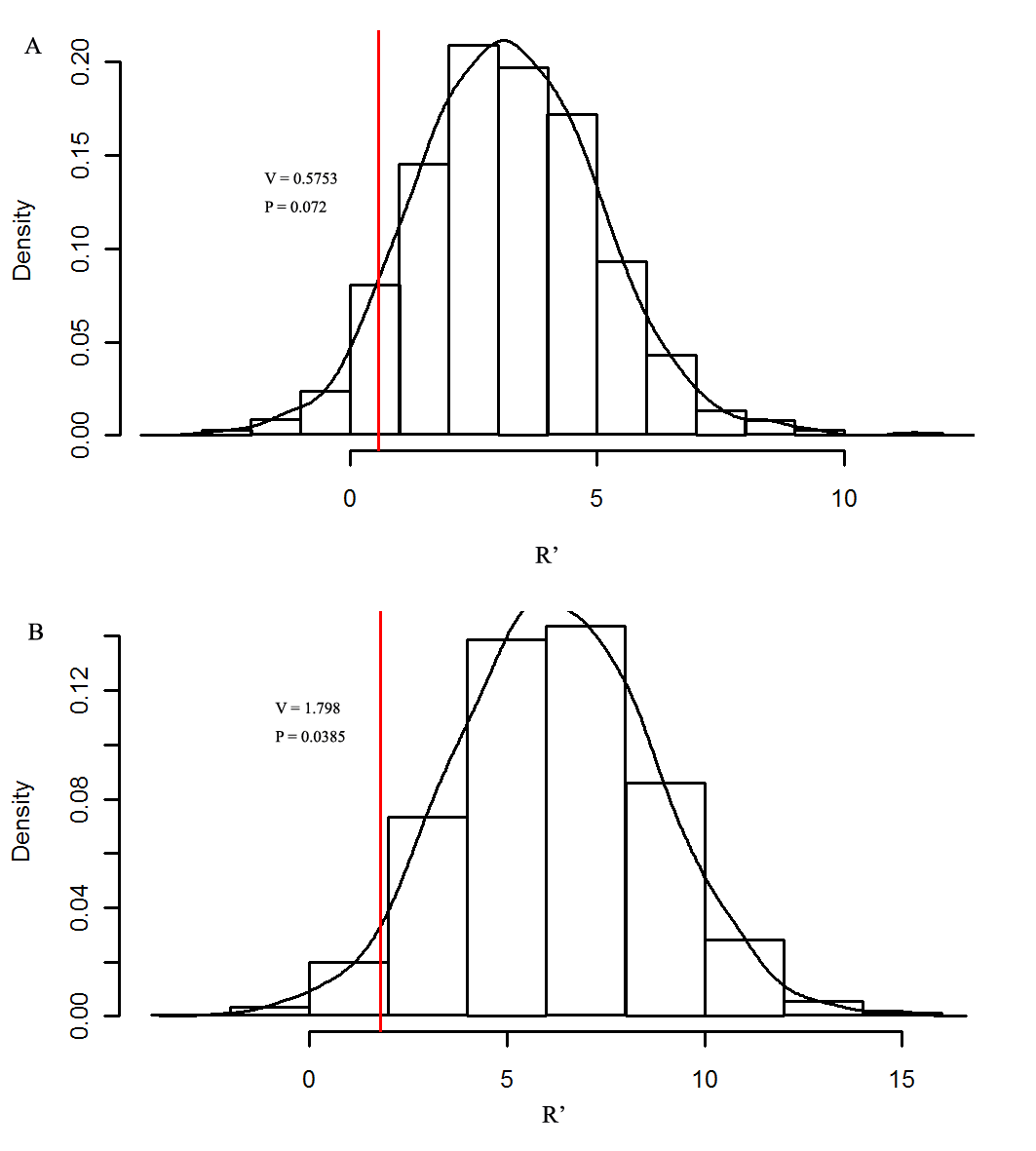


Figure S2. The distribution of R’ [the difference of mean R between Africans (YRI) and Europeans(CEU)] in permutation test and the difference of mean R for lipid metabolism between African and European (V = R’_African-European_ = mean R_African_ – mean R_European_, is shown as the red line in figure) in 1000 Genome data. In permutation test, the re-sampling process repeats 2000 times. In subplot A are results in the catabolic process; and in subplot B are results in the biosynthetic process.


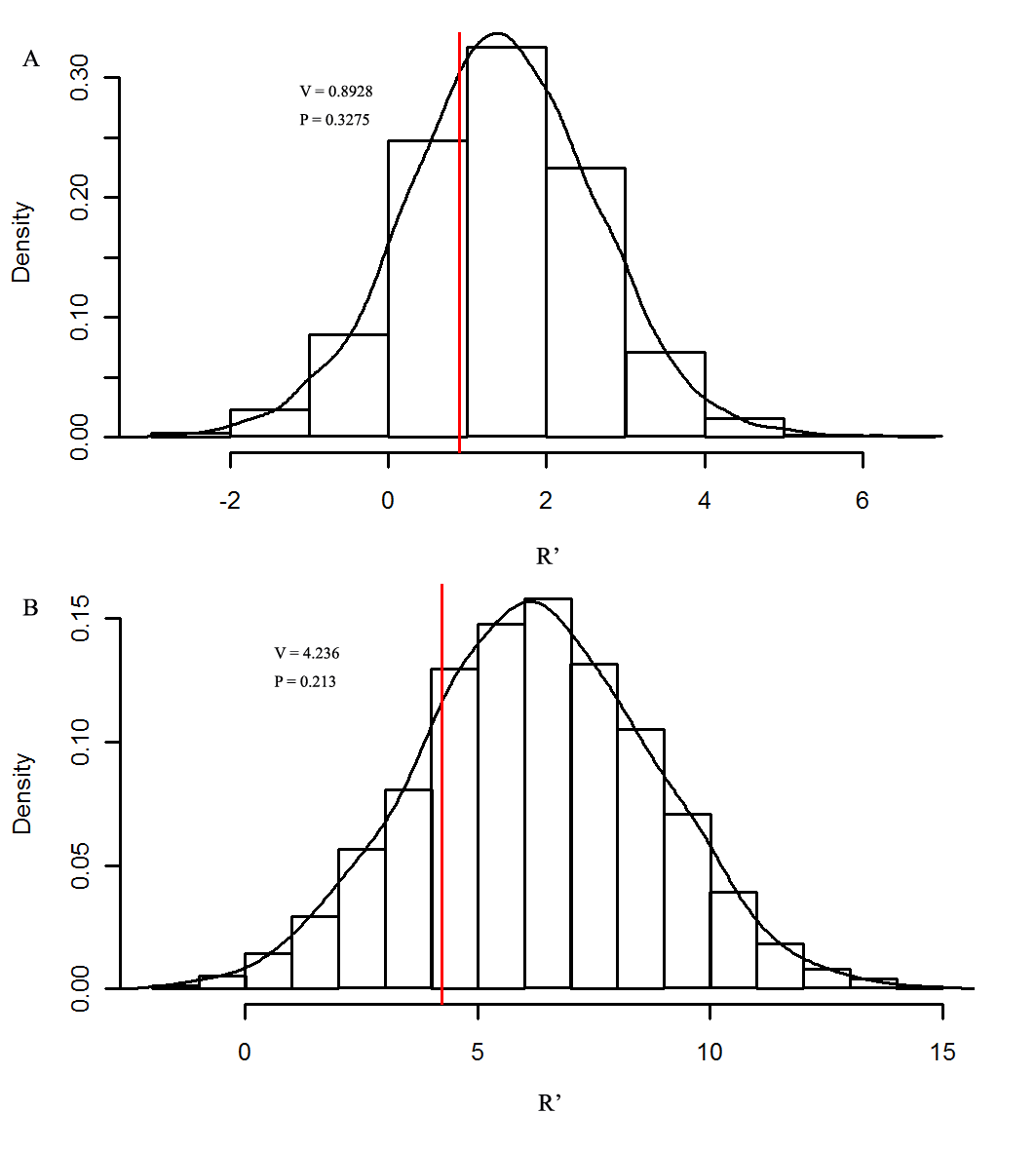


Figure S3. The distribution of R’ [the difference of mean R between Africans (YRI) and Europeans(CEU)] in permutation test and the difference of mean R for amino acid metabolism between African and European (V = R’_African-European_ = mean R_African_ – mean R_European_, is shown as the red line in figure) in 1000 Genome data. In permutation test, the re-sampling process repeats 2000 times. In subplot A are results in the catabolic process; and in subplot B are results in the biosynthetic process.


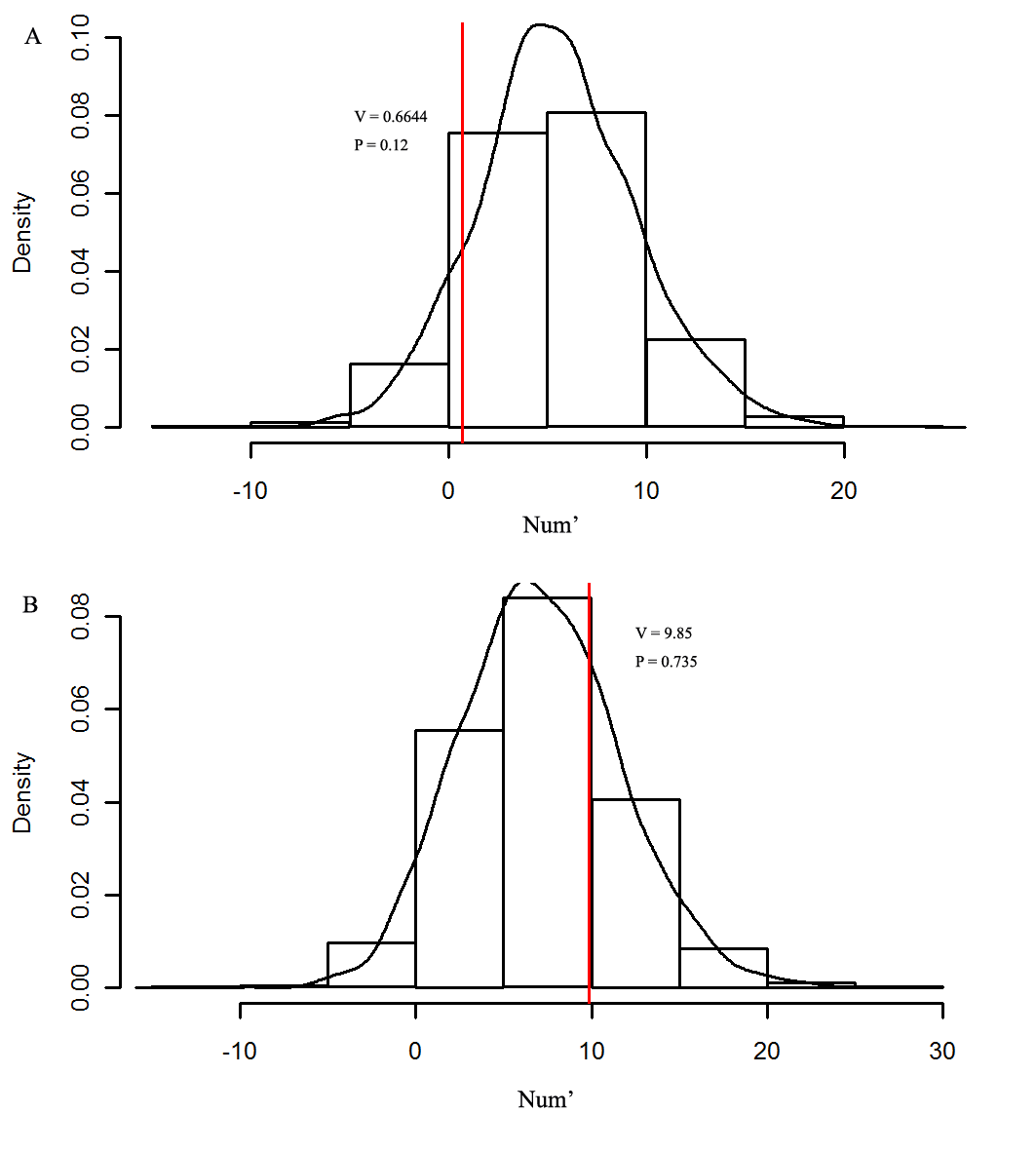


Figure S4. The distribution of Num’ [the difference of mean Num between Africans (YRI) and Europeans(CEU)] in permutation test and the difference of mean Num for carbohydrate metabolism between African and European (V = Num’_African-European_ = mean Num_African_ – mean Num_European_, is shown as the red line in figure) in 1000 Genome data. In permutation test, the re-sampling process repeats 2000 times. In subplot A are results in the catabolic process; and in subplot B are results in the biosynthetic process.


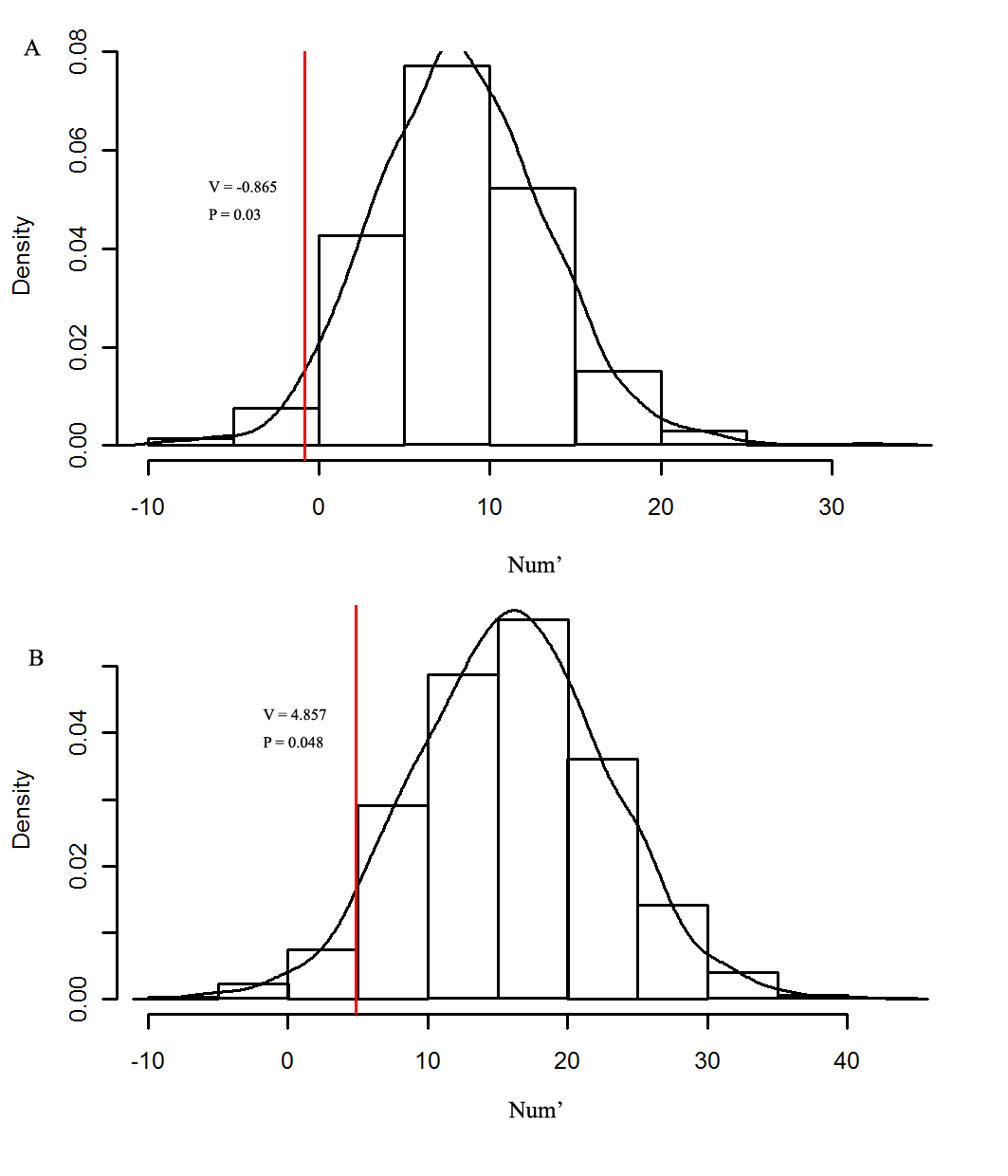


Figure S5. The distribution of Num’ [the difference of mean Num between Africans (YRI) and Europeans(CEU)] in permutation test and the difference of mean Num for lipid metabolism between African and European (V = Num’_African-European_ = mean Num_African_ – mean Num_European_, is shown as the red line in figure) in 1000 Genome data. In permutation test, the re-sampling process repeats 2000 times. In subplot A are results in the catabolic process; and in subplot B are results in the biosynthetic process.


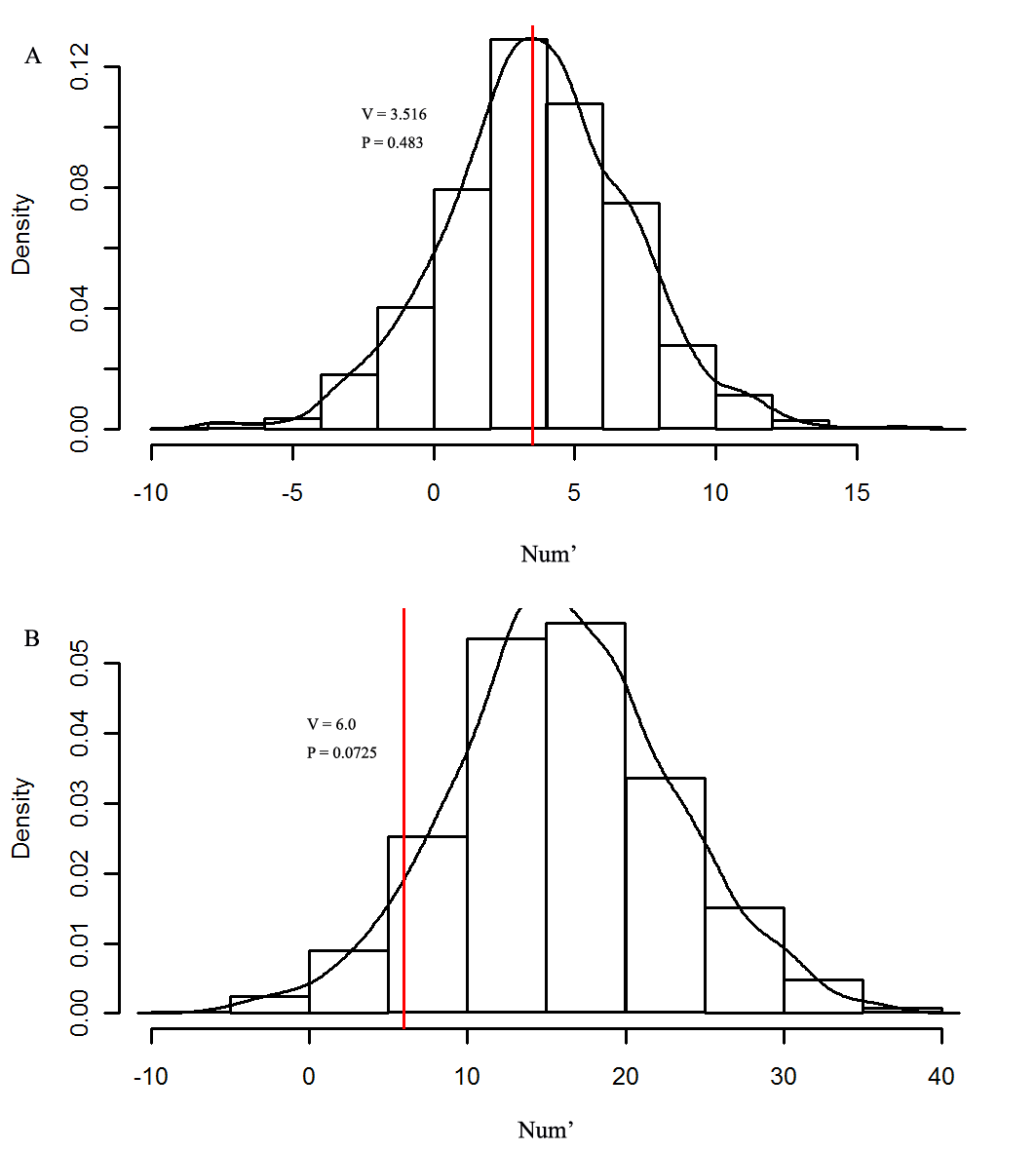


Figure S6. The distribution of Num’ [the difference of mean Num between Africans (YRI) and Europeans(CEU)] in permutation test and the difference of mean Num for amino acid metabolism between African and European (V = Num’_African-European_ = mean Num_African_ – mean Num_European_, is shown as the red line in figure) in 1000 Genome data. In permutation test, the re-sampling process repeats 2000 times. In subplot A are results in the catabolic process; and in subplot B are results in the biosynthetic process.


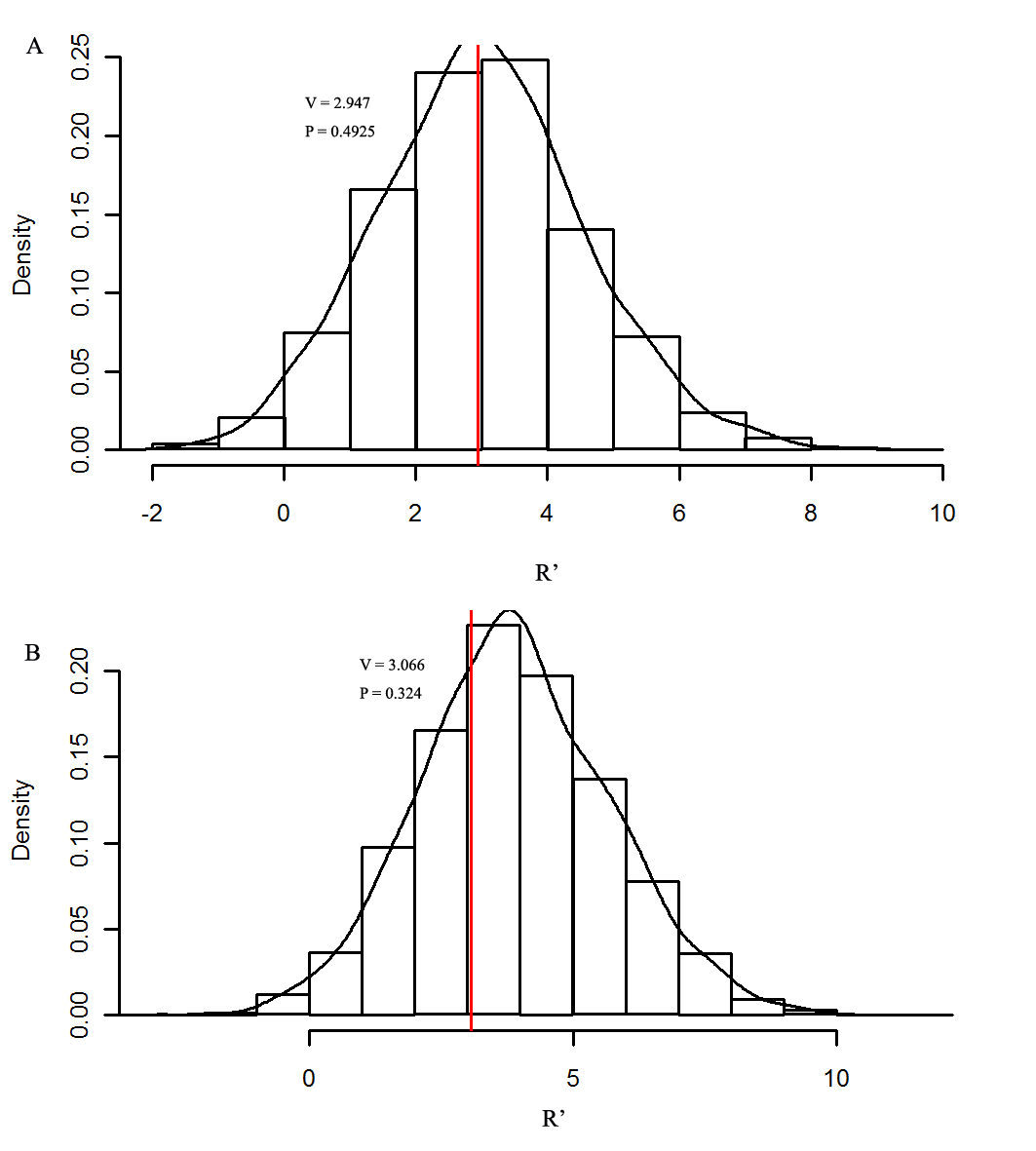


Figure S7. The distribution of R’ [the difference of mean R between Africans (YRI) and Asians(CHBJPT)] in permutation test and the difference of mean R for carbohydrate metabolism between African and Asian (V = R’_African-Asian_ = mean R_African_ – mean R_Asian_, is shown as the red line in figure) in 1000 Genome data. In permutation test, the re-sampling process repeats 2000 times. In subplot A are results in the catabolic process; and in subplot B are results in the biosynthetic process.


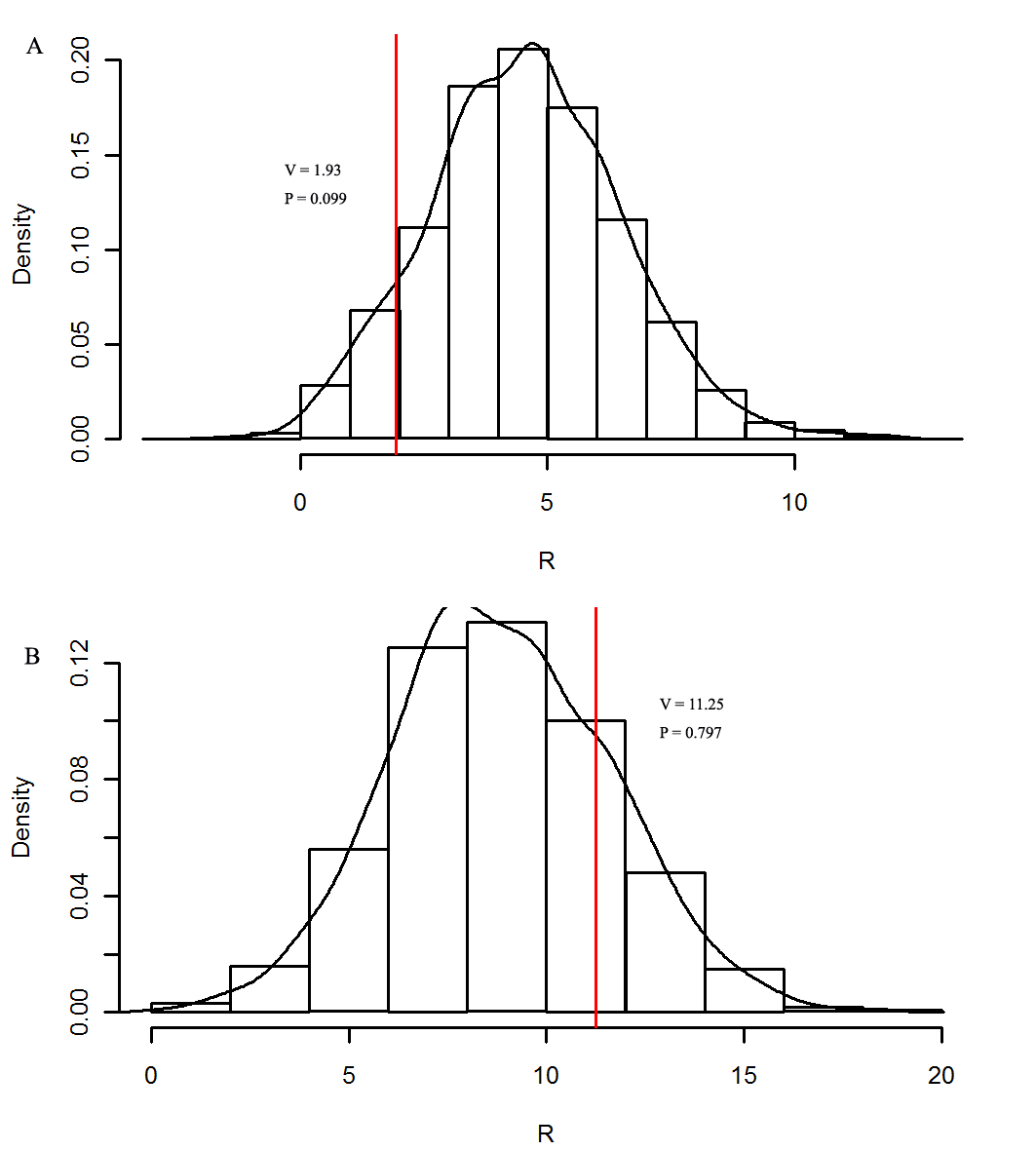


Figure S8. The distribution of R’ [the difference of mean R between Africans (YRI) and Asians(CHBJPT)] in permutation test and the difference of mean R for lipid metabolism between African and Asian (V = R’_African-Asian_ = mean R_African_ – mean R_Asian_, is shown as the red line in figure) in 1000 Genome data. In permutation test, the re-sampling process repeats 2000 times. In subplot A are results in the catabolic process; and in subplot B are results in the biosynthetic process.


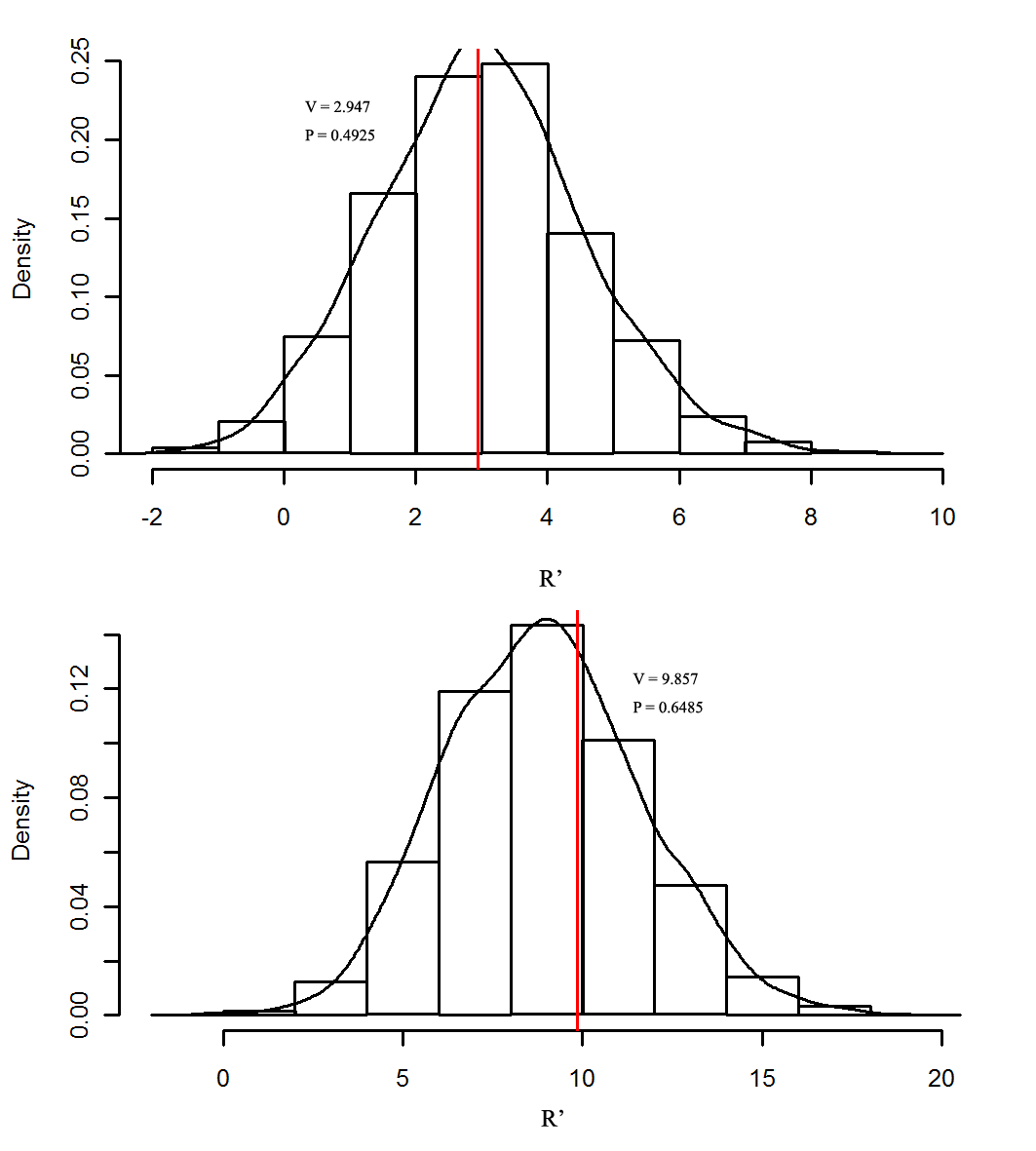


Figure S9. The distribution of R’ [the difference of mean R between Africans (YRI) and Asians(CHBJPT)] in permutation test and the difference of mean R for amino acid metabolism between African and Asian (V = R’_African-Asian_ = mean R_African_ – mean R_Asian_, is shown as the red line in figure) in 1000 Genome data. In permutation test, the re-sampling process repeats 2000 times. In subplot A are results in the catabolic process; and in subplot B are results in the biosynthetic process.


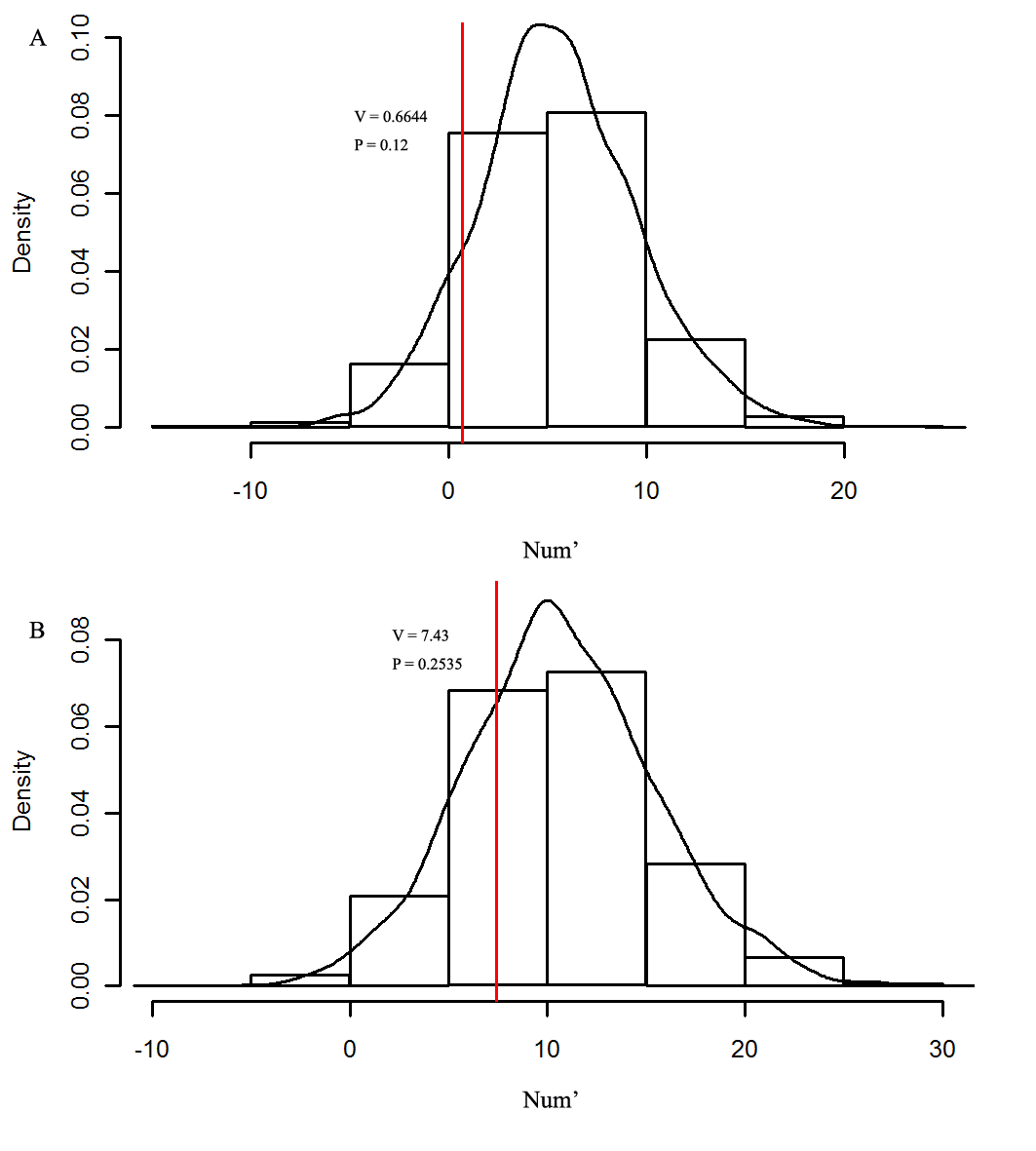


Figure S10. The distribution of Num’ [the difference of mean Num between Africans (YRI) and Asians(CHBJPT)] in permutation test and the difference of mean Num for carbohydrate metabolism between African and Asian (V = Num’_African-Asian_ = mean Num_African_ – mean Num_Asian_, is shown as the red line in figure) in 1000 Genome data. In permutation test, the re-sampling process repeats 2000 times. In subplot A are results in the catabolic process; and in subplot B are results in the biosynthetic process.


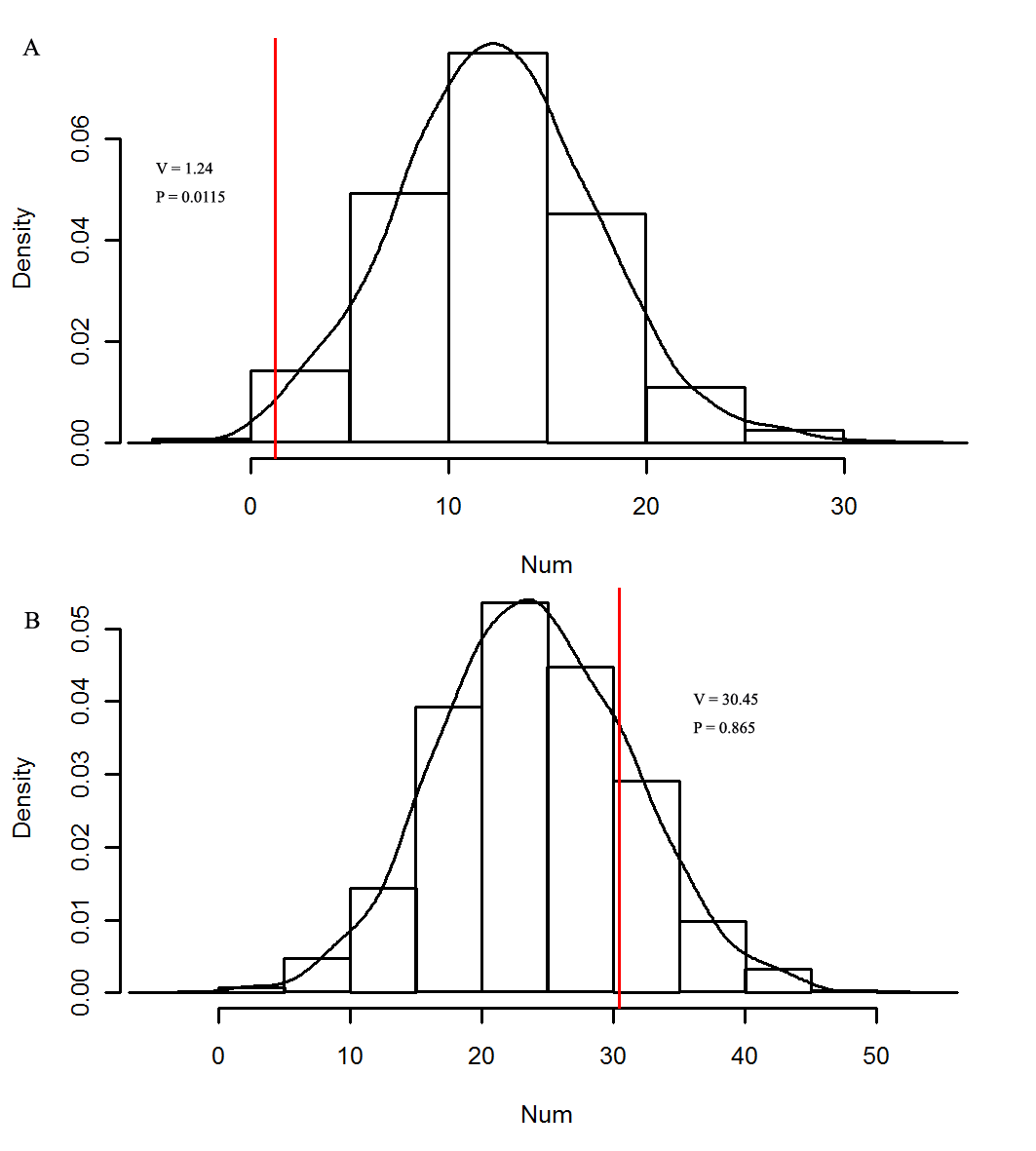


Figure S11. The distribution of Num’ [the difference of mean Num between Africans (YRI) and Asians(CHBJPT)] in permutation test and the difference of mean Num for lipid metabolism between African and Asian (V = Num’_African-Asian_ = mean Num_African_ – mean Num_Asian_, is shown as the red line in figure) in 1000 Genome data. In permutation test, the re-sampling process repeats 2000 times. In subplot A are results in the catabolic process; and in subplot B are results in the biosynthetic process.


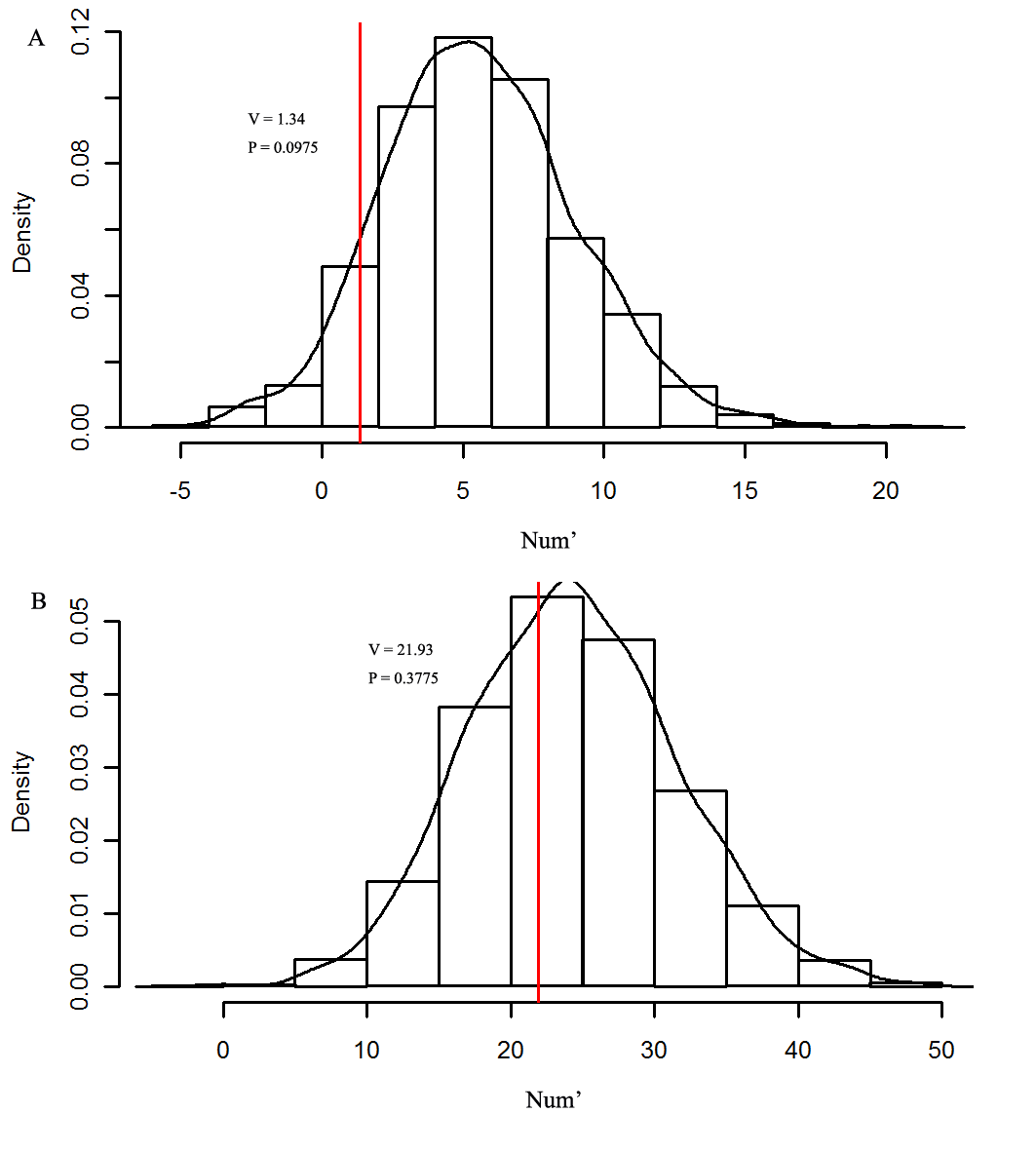


Figure S12. The distribution of Num’ [the difference of mean Num between Africans (YRI) and Asians(CHBJPT)] in permutation test and the difference of mean Num for amino acid metabolism between African and Asian (V = Num’_African-Asian_ = mean Num_African_ – mean Num_Asian_, is shown as the red line in figure) in 1000 Genome data. In permutation test, the re-sampling process repeats 2000 times. In subplot A are results in the catabolic process; and in subplot B are results in the biosynthetic process.


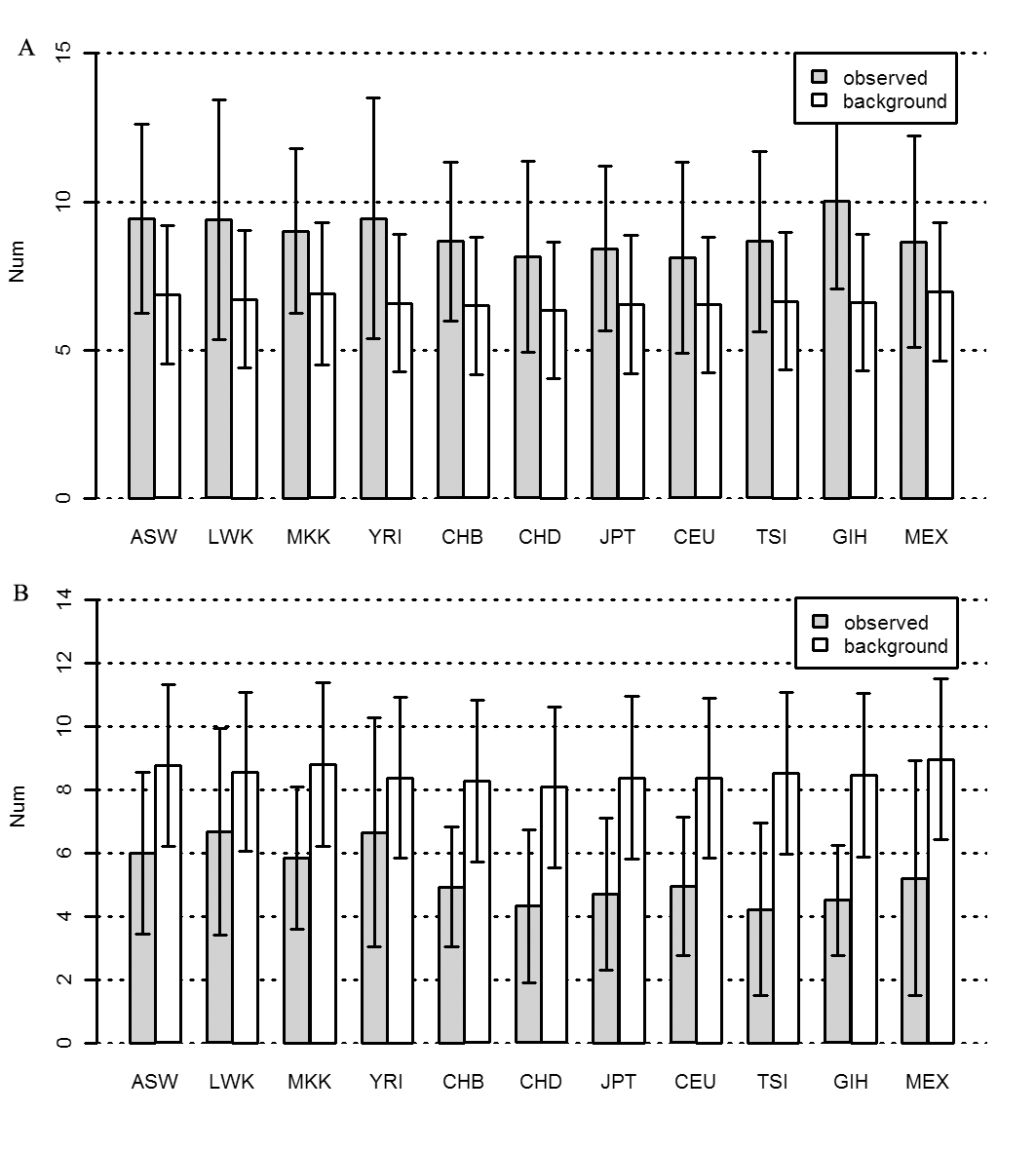


Figure S13. The observed (grey bar) and background (white bar) genetic risk (Num) for Hapmap dataset in the carbohydrate catabolic and biosynthetic processes. (The background values were obtained using permutation test).

A: Results for the carbohydrate catabolic process

B: Results for the carbohydrate biosynthetic process


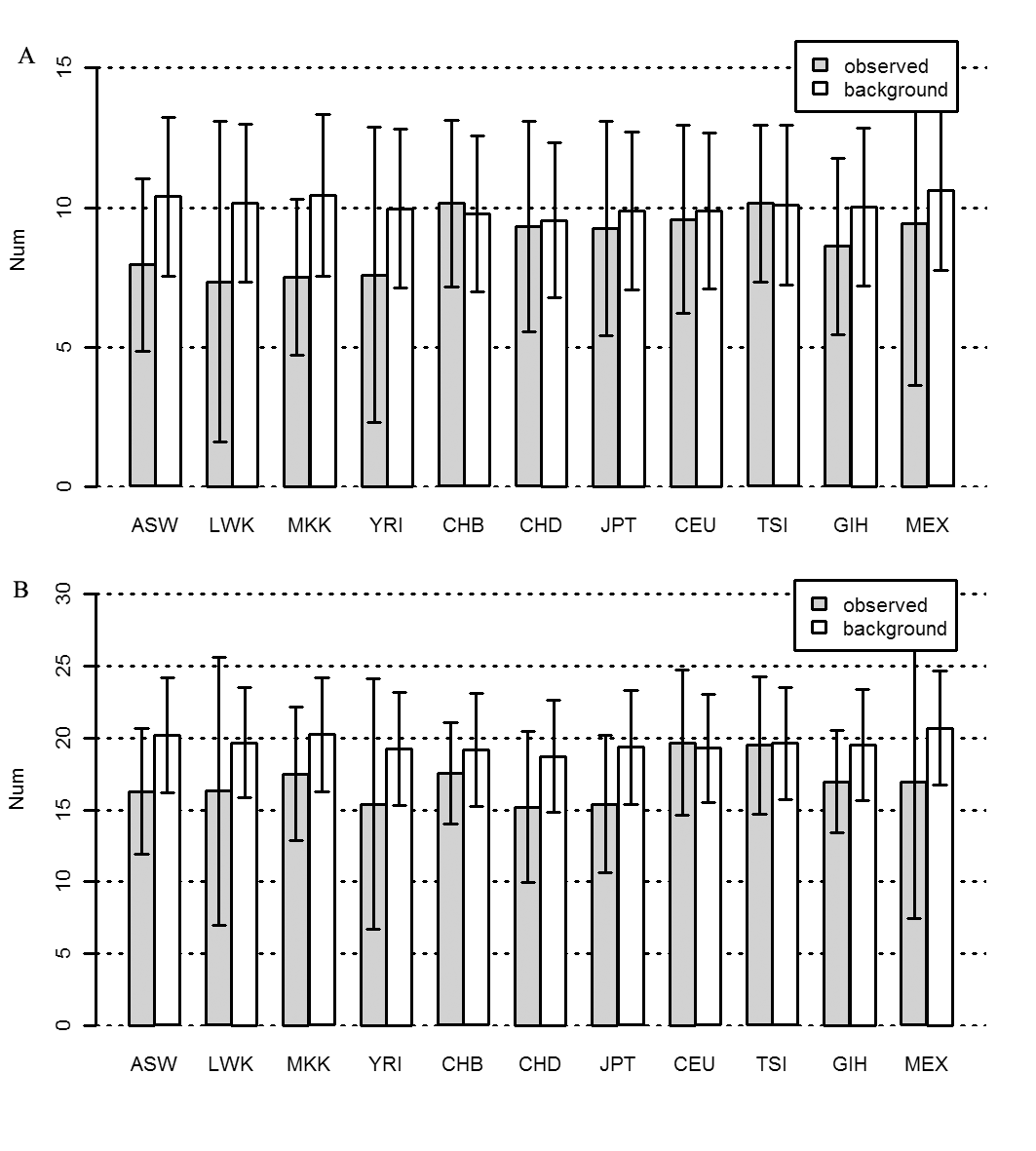


Figure S14. The observed (grey bar) and background (white bar) genetic risk (Num) for Hapmap dataset in the lipid catabolic and biosynthetic processes. (The background values were obtained using permutation test).

A: Results for the lipid catabolic process

B: Results for the lipid biosynthetic process


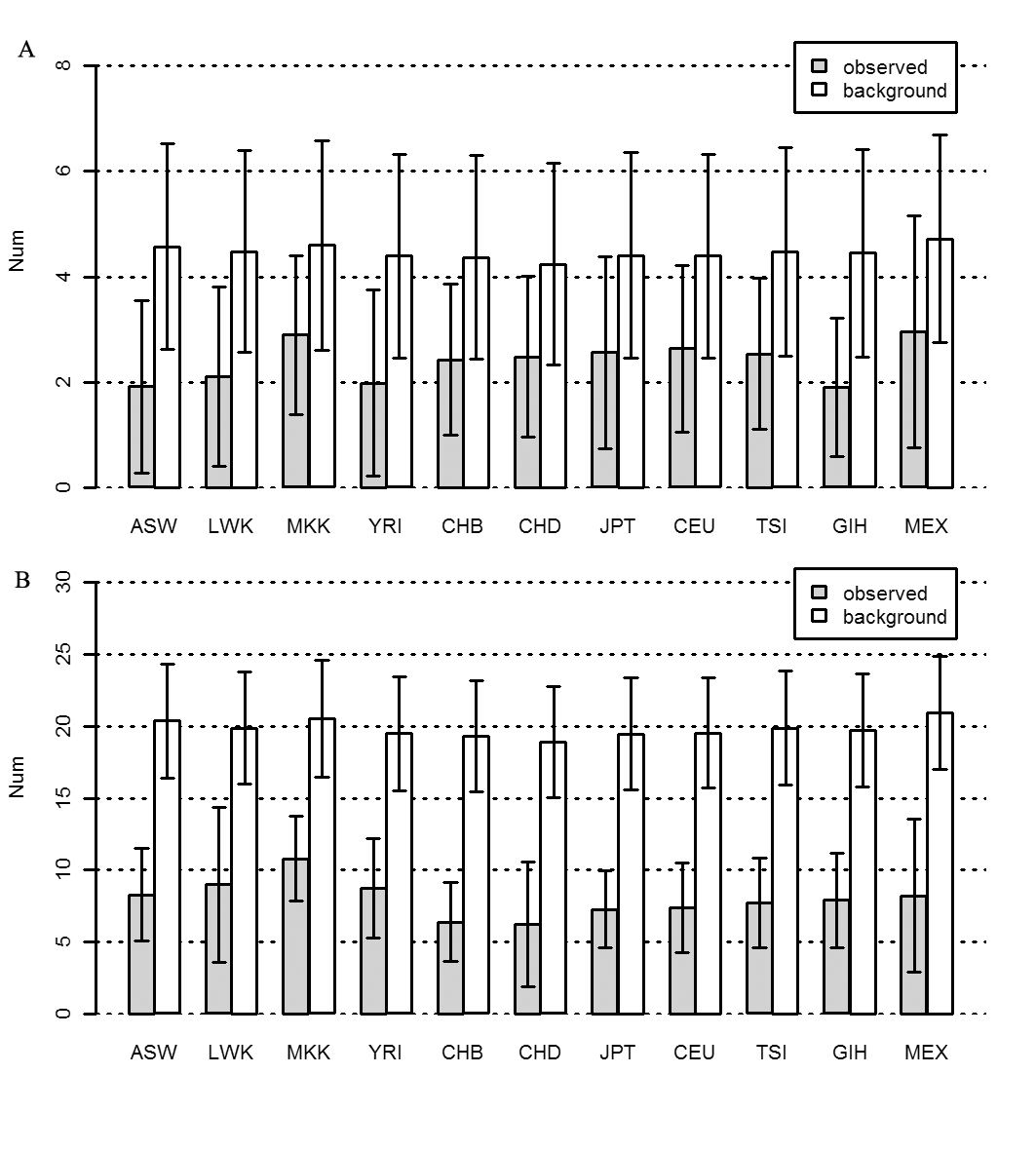


Figure S15. The observed (grey bar) and background (white bar) genetic risk (Num) for Hapmap dataset in the amino acid catabolic and biosynthetic processes. (The background values were obtained using permutation test).

A: Results for the amino acid catabolic process

B: Results for the amino acid biosynthetic process
